# Unraveling the role of proteins in dementia: insights from two UK cohorts with causal evidence

**DOI:** 10.1093/braincomms/fcaf097

**Published:** 2025-03-03

**Authors:** Jessica Gong, Dylan M Williams, Shaun Scholes, Sarah Assaad, Feifei Bu, Shabina Hayat, Paola Zaninotto, Andrew Steptoe

**Affiliations:** Department of Epidemiology and Public Health, University College London, London WC1E 7HB, UK; George Institute for Global Health, Imperial College London, London W12 7RZ, UK; MRC Unit for Lifelong Health & Ageing, University College London, London WC1E 7HB, UK; Department of Epidemiology and Public Health, University College London, London WC1E 7HB, UK; Department of Epidemiology and Public Health, University College London, London WC1E 7HB, UK; Department of Behavioural Science and Health, University College London, London WC1E 7HB, UK; Department of Epidemiology and Public Health, University College London, London WC1E 7HB, UK; Department of Epidemiology and Public Health, University College London, London WC1E 7HB, UK; Department of Behavioural Science and Health, University College London, London WC1E 7HB, UK

**Keywords:** dementia, proteomics, Mendelian randomization, ELSA, UK biobank

## Abstract

Population-based proteomics offers a groundbreaking avenue to predict future disease risks, enhance our understanding of disease mechanisms, and discover novel therapeutic targets and biomarkers. The role of plasma proteins in dementia, however, requires further exploration. This study investigated 276 protein-dementia associations in 229 incident all-cause dementia, 89 Alzheimer’s disease, and 41 vascular dementia among 3249 participants (55% women, 97.2% white ethnicity) from the English Longitudinal Study of Ageing (ELSA) over a median 9.8-year follow-up. We used Cox proportional hazard regression for the analysis. Receiver operating characteristic analyses were conducted to assess the precision of the identified proteins from the fully adjusted Cox regression models in predicting incident all-cause dementia, both individually and in combination with demographic predictors, *APOE* genotype, and memory score, to estimate the area under the curve. Additionally, the eXtreme Gradient Boosting machine learning algorithm was used to identify the most important features predictive of future all-cause dementia onset. These associations were then validated in 1506 incident all-cause dementia, 732 Alzheimer’s disease, 281 vascular dementia, and 111 frontotemporal dementia cases among 52 745 individuals (53.9% women, 93.3% White ethnicity) from the UK Biobank over a median 13.7-year follow-up. Two-sample bi-directional Mendelian randomization and drug target Mendelian randomization were further employed to determine the causal direction between protein concentration and dementia. NEFL (hazard ratio [HR] [95% confidence intervals (CIs)]: 1.54 [1.29, 1.84]) and RPS6KB1 (HR [95% CI]: 1.33 [1.16, 1.52]) were robustly associated with incident all-cause dementia; MMP12 (HR [95% CI]: 2.06 [1.41, 2.99]) was associated with vascular dementia in ELSA, after correcting for multiple testing. Additional markers EDA2R and KIM1 were identified from subgroup and sensitivity analyses. Combining NEFL and RPS6KB1 with other predictors yielded high predictive accuracy (area under the curve = 0.871) for incident all-cause dementia. The eXtreme Gradient Boosting machine learning algorithm also identified RPS6KB1, NEFL, and KIM1 as the most important protein features for predicting future all-cause dementia. Sex difference was evident for the association between RPS6KB1 and all-cause dementia, with stronger association in men (*P* for interaction = 0.037). Replication in the UK Biobank confirmed the associations between the identified proteins and various dementia subtypes. The results from Mendelian randomization in the reverse direction indicated that several proteins serve as early markers for dementia, rather than being direct causes of the disease. These findings provide insights into putative mechanisms for dementia. Future studies are needed to validate the findings on RPS6KB1 in relation to dementia risk.

## Teaser

Gong *et al*. reported NEFL and RPS6KB1 as robustly associated with all-cause dementia, MMP12 with vascular dementia in the English Longitudinal Study of Ageing. Validation in UK Biobank confirmed these findings. Combining biomarkers with predictors achieved high predictive accuracy. Machine learning and Mendelian randomization highlight potential early markers and mechanisms.

## Graphical Abstract

**Graphical Abstract:**
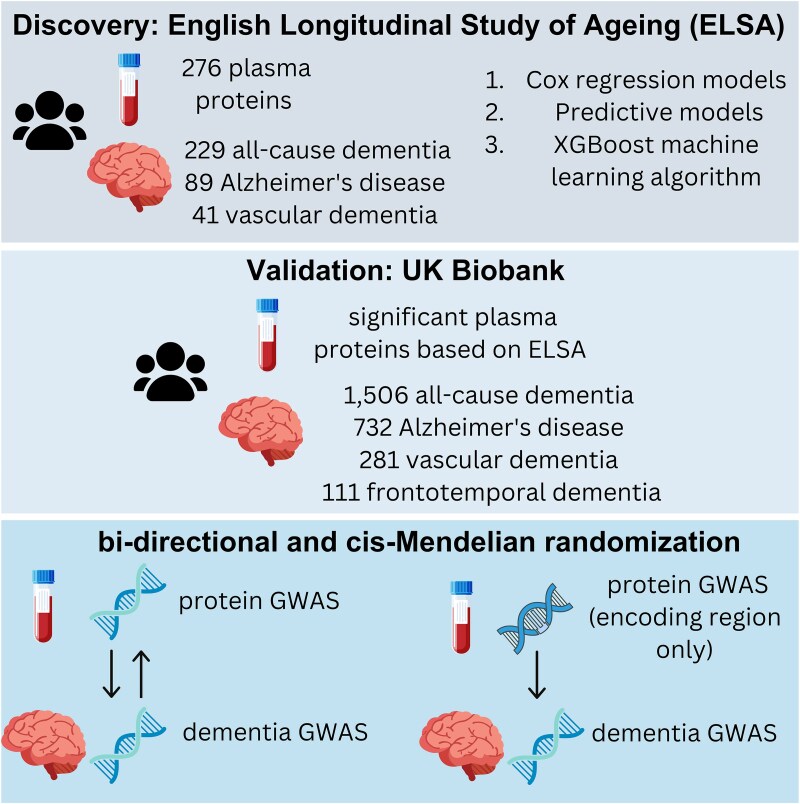

## Introduction

The understanding of Alzheimer’s disease and related dementia (ADRD) is increasingly shifting towards a systemic and multifactorial perspective.^1,2^ Circulating proteins, as pivotal agents in biological processes, offer direct insights into disease mechanisms and can serve as early indicators, regulators, and effectors in disease pathways. This renders their studies indispensable in both drug discovery and diagnostics development.^3,4^

Mounting evidence supports the significance of proteomics in exploring pathways involved in ADRD.^3,5-7^ At the molecular level, deviations in protein function or expression play a role in the pathogenesis of prodromal dementia,^3,8,9^ while protein biomarkers can forecast disease onset several years before symptoms manifest.^3,8-10^ Remarkably, approximately 96% of currently approved drugs target proteins,^4,11^ underscoring the substantial value of proteomics in ADRD drug discovery.

Integrating large-scale proteomics data into population studies represents a recent development,^3,12^ enabling cost-effective simultaneous measurement of multiple proteins on many samples.^3,13,14^ This has led to the identification of distinct protein signatures relevant to ADRD susceptibility.^4,9,10,15-18^ Longitudinal analyses in the UK Whitehall II study spanning across two decades, demonstrated associations between 15 non-amyloid/non-tau-related proteins and cognitive decline and dementia.^4^ Similarly, the Atherosclerosis Risk in Communities (ARIC) study in the US identified significant protein signatures for dementia, including immune and proteostasis/autophagy pathways.^10^ Intriguingly, some of these associations were independent of known Alzheimer’s disease risk factors, suggesting novel potential targets for intervention.^4,9,10^ Recent analyses from the UK Biobank (UKB) identified few known proteins associated with incident all-cause dementia (ACD), Alzheimer’s disease, and vascular dementia (VAD).^16,17^ However, previous studies utilized the aptamer-based SomaScan platform in ARIC and Whitehall II,^4,9,10^ and the SomaScan platform is deemed to have lower specificity compared with the Olink platform, which employs multiplexed antibody-based immunoassays proximity extension assay technology.^19^ Moreover, the UKB studies faced limitations such as a lack of external validation cohorts and confirming relationships via causal inference such as Mendelian Randomization (MR).^16,17^

In this current study, we employed the large-scale Olink proteomics platform and a robust dementia algorithm to assess the proteomic signature of dementia risk in over 3000 older adults using data from the English Longitudinal Study of Ageing (ELSA) as the discovery cohort. We validated these findings using Olink proteomics data from over 50 000 participants from the UKB.^20^ Two-sample bi-directional MR and drug target MR were utilized to infer causality between protein concentration and dementia outcomes, leveraging summary statistics from large genome-wide association study (GWAS) consortia.

## Materials and methods

### Consent statements

Ethical consent has been obtained for all waves and components of ELSA, according to the ethical approval system in operation at the time and in accordance with the Declaration of Helsinki.

UK Biobank has approval from the Northwest Multi-center Research Ethics Committee as a Research Tissue Bank approval.

### ELSA discovery cohort study population

ELSA is a nationally representative sample of men and women aged 50 years and over living in England. Data collection started in 2002–2003, with participants interviewed every two years. Details of study design are available elsewhere.^21^ Blood sample collection in ELSA took place for the first time in wave 2 nurse visit in 2004–2005 and subsequently in every 4-year interval. The blood collected from wave 4 nurse visits in 2008–2009 were used for the proteomics profiling (thus forming the baseline sample of this study), thereby affording a temporal perspective that allows for the exploration of the relationship between protein concentrations and ADRD over 10 years. The following exclusion criteria were applied: (i) participants who died within 2 years since the wave 4 nurse visit or (ii) participants lost to follow-up (missing at ≥2 waves). A total of 3305 available plasma samples from wave 4 were retrieved for the proteomics profiling.

The proteomics dataset in ELSA was curated with a focus on investigating the underlying biological processes associated with ADRD and cognitive decline. The assays encompassed an extensive array of cardiovascular and inflammatory markers, in addition to markers integral to neurological processes such as axon guidance, neurogenesis, and synapse assembly. These analyses were conducted utilizing the Olink proteomics platform, the antibody based Olink proximity extension assay technology.^22^ We thereby used Olink Target 96 Neurology, Cardiovascular II and Neurology Exploratory arrays in these analyses. Frozen samples were shipped to Olink for aliquoting, plating, and assays. These assays include a built-in quality control based on four internal controls that are spiked into all samples, and external controls. Following stringent data quality control (Supplementary Methods), proteins were measured across three panels containing 276 proteins. Proteins were presented as NPX values, the arbitrary unit on log^2^ scale from Olink. After excluding those who did not pass quality control, a combined dataset comprising of 3262 samples were included.

### Covariates assessment in ELSA

Baseline (at wave 4, 2008–2009) sociodemographic and socioeconomic covariates including age (in years), biological sex (male versus female) and ethnicity (white versus other ethnic groups) were self-reported. The age that participants left formal education was coded as follows: none, age 14 or under, 15, 16, 17, 18, 19 or over. Smoking status was self-reported and was categorized as never, former and current smoker. Physician-diagnosed cardiovascular disease (heart attack, angina or heart failure) was self-reported. Depression was also self-reported. Body mass index was calculated using participant’s height and weight measured during the nurse visit. Three measurements were taken of systolic blood pressure on the respondent’s right arm while they were seated, and the average of the three measurements was used. Low-density lipoprotein cholesterol was assayed using the blood sample collected by the nurse. The APOE genotype was derived from the analysis of two specific SNPs, namely rs7412 and rs429358. To determine these genotypes, two TaqMan assays from Assay-On-Demand, a product of Applied Biosystems and Gene service Ltd in Cambridge, UK, were employed. These assays were conducted on a 7900HT analyzer, manufactured by Applied Biosystems, and the genotypes were determined using the Sequence Detection Software (version 2.0), also from Applied Biosystems. The quality control of genome-wide genotyping has been described elsewhere.^23^ Episodic memory was assessed at wave 4, evaluated through the immediate and delayed recall tasks of the Consortium to Establish a Registry for Alzheimer’s disease.^24^ Participants were presented with a ten-word list and tasked with recalling it both immediately and after a delay. The scores from these tests were aggregated to compute a memory score.

### Dementia algorithm in ELSA

The standardized algorithm for identifying dementia cases relied on five primary data sources: (i) coded information extracted from interviews across all waves using participant self-reported physician diagnosis of Alzheimer’s disease and dementia; (ii) caregivers who completed a modified short-form Informant Questionnaire on Cognitive Decline in the Elderly; (iii) medication data collected during nurse visits (wave 6, 8, and 9); (iv) linked data from hospital admissions (NHS Hospital Episode Statistics); and (v) mortality records (Office for National Statistics Mortality Statistics). All data sources were integrated into algorithm development (see Supplementary Table 1A for ICD-10 codes used for ascertaining ACD).

Dementia subtypes, specifically Alzheimer’s disease and VAD, were also derived (Supplementary Table 1B).

The computation of time-to-event for dementia cases depended on the data source where dementia was first recorded. Further details on the dementia algorithm can be found in the Supplementary Methods.

Based on the algorithm, baseline prevalent dementia was excluded (*N*  *=* 13), which yielded a final analytical sample of 3249.

### UKB for validation

UKB is a large population-based cohort from the UK with over half a million participants aged 40–69 years, recruited between 2006 and 2010.^20^ Proteomics profiling was conducted in 54 219 participants at study baseline, with 2923 unique proteins assayed using the antibody based Olink Explore 3072 proximity extension assay, across eight Olink panels (Cardiometabolic I, Cardiometabolic II, Inflammation I, Inflammation II, Neurology I, Neurology II, and Oncology I, Oncology II). Consortium members opted for samples enriched in specific diseases of interest, while the remaining population was randomly sampled using a stratified approach based on age, sex, and recruitment center.^20^ The current analysis excluded those with baseline dementia, yielding a total sample of 52 745 individuals. The significant proteins identified ELSA were subsequently validated using UKB, if assayed.

We have attempted, where possible, to derive similar variables for ELSA and the UKB cohorts with consideration of the level of missingness, to maximize comparability. Participant’s age was derived based on date of birth and date of attending an initial assessment center. Participant’s biological sex was acquired from central registry at recruitment and contains a mixture of the sex recorded by the NHS and self-reported sex. Ethnicity was self-reported and categorized into White, Mixed, Asian, or Asian British, and Black or Black British. Highest qualification was determined by the answers provided to the question: ‘Which of the following qualifications do you have?’, with options included: College or University degree; NVQ (National Vocational Qualification) or HND (Higher National Diploma) or HNC (Higher National Certificate) or equivalent; other professional qualifications e.g.: nursing; A levels/AS levels; O levels/GCSEs (General Certificate of Secondary Education) or equivalent; CSEs (General Certificate of Secondary Education) or equivalent; or none of the above. Smoking status was self-reported and categorized as never, former, and current smoker. Self-reported medical conditions were solicited through the touchscreen questionnaire as well as during verbal interview conducted by a trained nurse, and the presence of cardiovascular disease (heart attack, angina, or heart failure) were defined if the participant reported any of these conditions. Depression was affirmatory if the participant confirmed to have any of probable recurrent major depression (severe), probable recurrent major depression (moderate), or single probable major depression episode, if reported on the questionnaire or nurse-administered verbal interview. Body mass index was constructed from height and weight measured during the initial assessment center visit using an Omron device. Two automatic readings of blood pressure were taken a few moments apart, using an Omron HEM-7015IT digital blood pressure monitor, and the average of the readings were used. low-density lipoprotein cholesterol was measured by enzymatic protective selection analysis on a Beckman Coulter AU5800 from the blood sample collected at recruitment.

Incident ACD, Alzheimer’s disease, VAD and FTD were defined by the UKB dementia algorithm.^25^ The last date of censoring was 31 December 2022 (last date of linkage to death and inpatient records). Baseline dementia was removed.

### Statistical analysis

#### Analysis 1: protein-dementia associations in the ELSA discovery cohort

We used Cox proportional hazards regression models to evaluate the associations between each plasma protein NPX value and incident dementia using the ‘survival’ R package.

All proteins had ≤6% missing, with missingness largely due to failure in passing internal quality control. Rank-based inverse normal transformation was first applied to the protein levels given that imputation is done using Euclidean distances and scaled to have a mean of 0 and standard deviation of 1 prior to all analyses. Missing protein measurements were imputed using the K-nearest neighbor (*k* = 57) imputation using the ‘impute’ R package,^26^ which works by identifying the nearest 57 individuals defined using Euclidean distances and imputing with their medians, with k calculated from the square root of the total sample size (*N* = 3249). Missingness was imputed for proteomics data using K-nearest neighbor, together with clinical data using the Multiple Imputation by Chained Equations procedure (‘mice’ R package).^27^ A total of 30 imputed datasets were generated, with the imputation procedure iterating 10 times to ensure stable and reliable estimates. Additionally, as part of the sensitivity analysis, a Multiple Random Forest Regression Imputation approach was employed to impute missing data from the full dataset with scaled protein levels. This was done using 30 imputed datasets and 10 iterations for the main analysis in ELSA, allowing for consideration of non-linear relationships within the data and minimizing potential biases introduced by missingness.^28^

Based on the imputed datasets, we first assessed the protein-dementia associations without any model adjustments by pooling the estimates from all 30 imputed datasets. The models were further adjusted for age, sex, and ethnicity for the minimally-adjusted model; and adjusted for age, sex, ethnicity, education, smoking status, depression, cardiovascular disease, body mass index, systolic blood pressure, and low-density lipoprotein cholesterol for the fully-adjusted models for each protein NPX value associated with the risk of incident dementia, with false discovery rate (FDR)-corrected *P*-value (denoted as P_FDR_) set at a cut-off of 0.05, this translates to an uncorrected *P*-value of 0.00018. All *P*-values were two-sided. P_FDR_ were reported and displayed using volcano plot, accompanying the HR.

To evaluate the potential interactions in the Cox regression models, we incorporated interaction terms between protein levels and key demographic variables. The following interactions were explored: (i) protein × sex; (ii) protein × age; (iii) protein × age squared (age²); and (iv) protein × age² × sex.

The Cox proportional hazard regression analyses were repeated for Alzheimer’s disease and VAD separately.

A series of sensitivity analyses were conducted using the same adjustment methods as the main Cox regression for incident ACD, by excluding the following: (1) dementia cases that occurred during the first year of follow-up to reduce the possibility of reverse causation bias; (2) other ethnic groups other than white ethnicity; (3) APOE ε4 carriers; and (4) participants < 60 years of age. We also conducted competing risk of death using Fine-Gray regression models^29^ with the same covariate adjustments, given that death may preclude dementia from occurring. The competing risk models, which estimated sub-distribution HRs, were conducted for incident ACD accounting for all-cause mortality as a competing risk, incorporating time-to-event data.^29,30^

Next, receiver operating characteristic analyses were conducted to assess the precision of the identified proteins from the fully adjusted Cox regression models in predicting incident ACD. These analyses were performed independently and in combination with additional factors including age, sex, ethnicity, education, APOE ε4 status and memory score. To evaluate the performance of the Cox models, bootstrapping was performed to assess the stability of the AUC estimates. A total of 2000 bootstrap resamples were generated. Bootstrapping involves resampling with replacement from the original dataset to estimate the sampling distribution of a statistic. We used the bootstrapped samples to compute 95% CI for the AUC of each model, utilizing the R packages ‘caret’,^31^ ‘boot’^32^ and ‘pROC’.^33^ The mean AUC value across all bootstrap resamples was calculated to estimate the overall predictive performance of the survival model.

We employed eXtreme Gradient Boosting (XGBoost), a powerful machine learning algorithm based on decision-tree ensembles within a gradient boosting framework, to identify the most important features predictive of future ACD, Alzheimer’s disease and VAD onset. To ensure a thorough and reliable analysis, we included all available protein, demographic and clinical predictors in our models, using imputed data to address missing values. The data were partitioned into two sets: 80% of the data were used as the training set to build and tune the XGBoost model, while the remaining 20% was reserved as the test set to evaluate the model's predictive performance. Feature importance scores generated by the XGBoost model were used to identify key predictors of ACD onset. To enhance the interpretability of these feature importance rankings, we employed SHapley Additive exPlanations (SHAP) values, which provide a consistent and theoretically grounded approach for explaining the contribution of individual features to the model’s predictions. SHAP values enabled us to gain a clearer understanding of how each predictor influenced the model’s outcomes. In addition, this process of evidence triangulation using XGBoost and SHAP values was conducted to complement and validate the findings from our Cox regression models. By comparing results from both the machine learning-based XGBoost approach and the statistical Cox regression models, we aimed to improve the robustness and reliability of our predictive models for future dementia onset.

#### Analysis 2: protein–dementia associations in the UKB validation cohort

The significant proteins from the main and sensitivity analyses based on ELSA were then validated using the UKB proteomics data, if assayed. The reason for choosing ELSA as the discovery cohort and UKB as the validation cohort is that ELSA’s target panels were specifically designed to investigate proteomic signatures related to cognitive decline and dementia, resulting in a more focused selection of proteins. In contrast, UKB offers a broader protein selection, allowing for effective validation.

The UKB proteomics samples underwent sample selection, processing, and quality control procedures.^20^ Missing protein measurements for the remaining individuals were imputed using K-nearest neighbor imputation (*k* = 230), the protein levels were normalized and scaled akin to the analyses in ELSA proteomics data described above.

Data were processed and analyzed using the R Studio Workbench on the UKB Research Analysis Platform, under application No.71702.

#### Analysis 3: two-sample bi-directional MR

We then assessed the potential causal relationships between the circulating protein concentration, in relation to dementia outcomes, using two-sample bi-directional MR. Summary statistics for genetic variants associated with the circulating protein levels, protein quantitative trait locus, which is also associated with dementia in GWAS were used to infer causality.

Selection of instruments to proxy for altered protein abundance were derived using protein quantitative trait locus mapping proteins that identifies genetic associations in participants of European ancestry from the UK Biobank based on Olink data (https://doi.org/10.7303/syn51364943), and genome-wide association meta-analysis based on 12 participating Olink cohorts from the SCALLOP Consortium for RPS6KB1 since it was not assayed in the UKB.^20,34^ The effects of protein protein quantitative trait locus were standardized to align with the same effect allele.

Methods for the MR analyses were detailed in the Supplementary Methods.

#### Analysis 4: two sample drug target MR (cis-MR)

Next, we used a two-sample MR study design, based predominantly on genetic variants located in or near genes that encode the relevant drug targets, to infer causality from protein concentration → dementia (cis-MR).^35-38^ Cis-MR is considered to be less susceptible to pleiotropy, and the potential effect of a drug by analyzing the genomic locus encoding protein targets, which may be informative for drug trial design.^36,38^

Methods for conducting drug target MR were further described in the Supplementary Methods.

#### Analysis 5: enrichment analysis

Enrichment analysis was conducted by searching open-source databases to further characterize the identified proteins from the Cox regressions. We employed the Enrichr,^39^ which is a computational method infers knowledge about an input gene set by comparing it to annotated gene sets that represent existing biological knowledge. It determines if the input set of genes significantly overlaps with these annotated gene sets. We used the full set of ELSA proteins as the background gene set, to glean a deeper biological understanding. We searched the following bioinformatics databases: Gene Ontology (GO): GO Molecular Function, GO Biological Process, and GO Cellular Component,^40^ Kyoto Encyclopedia of Genes and Genomes (KEGG),^41^ Reactome Pathway Database (REACTOME),^42^ Illuminating the Druggable Genome (IDG),^43^ Proteomics Drug Atlas (PDA),^44^ and Genotype-Tissue Expression (GTEx).^45^ Statistical significance was indicated if *P_FDR_* < 0.05.

Furthermore, we utilized the Open Targets platform (https://www.opentargets.org/) for the systematic identification of potential therapeutic drug targets among the identified proteins.^46^

All analyses were done using R Studio (version 4.4.1).

## Results

### Analysis 1: protein-dementia associations in the ELSA discovery cohort

The participant selection for the proteomics assay in ELSA is depicted in Supplementary Fig. 1. In 3262 samples with proteomics assayed, based on the dementia algorithm, prevalent dementia cases were excluded (*N* = 13), resulting in a final sample of 3249 in the analysis.

The mean age was 63.4 years (SD = 9.2), 55% were women, and 97.2% were of white ethnicity (Supplementary Table 2). A total of 229 incident ACD, 89 Ad and 41 VAD cases were documented over a median follow-up of 9.8 years (min–max: 0.4–10.9 years). Specific details on the data sources where these cases were extracted from in the first instance are included in Supplementary Fig. 2. The normalized protein expression levels for participants with no dementia, incident dementia and prevalent dementia are presented as box plots for each protein. *P*-values were calculated using the Kruskal–Wallis method, with adjustments for multiple comparisons using the FDR. These results are shown in Supplementary Figs 3–5 for each Olink Target 96 panel.

We initially assessed the relationship between the normalized protein expression value of 276 plasma proteins and ACD risk in the ELSA cohort, using cox proportional hazard regression models. Unadjusted analyses revealed that 95 measured proteins were significantly associated with ACD (Supplementary Fig. 6). Among these, NEFL exhibited the strongest association with ACD [*P_FDR_* = 8.66 × 10^−37^, hazard ratio; 95% confidence intervals (CIs): 3.01 (2.63, 3.44)], followed by EDA2R, SCARF2, LAYN, PGF, DCN, GFR-alpha-1, BNP, UNC5C, Dkk-4, KIM1 (also known as HAVCR1), TNFRSF12A, CADM3, TRAIL-R2, VWC2, and MMP12.

In the minimally adjusted models (adjusted for age, sex and ethnicity), NEFL [*P_FDR_* = 0.0002; HR (95% CI): 1.55 (1.30, 1.85)], RPS6KB1 [*P_FDR_* = 0.003; HR (95% CI): 1.34 (1.18, 1.53)], EDA2R [*P_FDR_* = 0.046; HR (95% CI): 1.43 (1.19, 1.72)] and KIM1 [*P_FDR_* = 0.049; HR (95% CI): 1.31 (1.14, 1.50)] were significantly associated with ACD (Supplementary Fig. 7).

**Figure 1:**
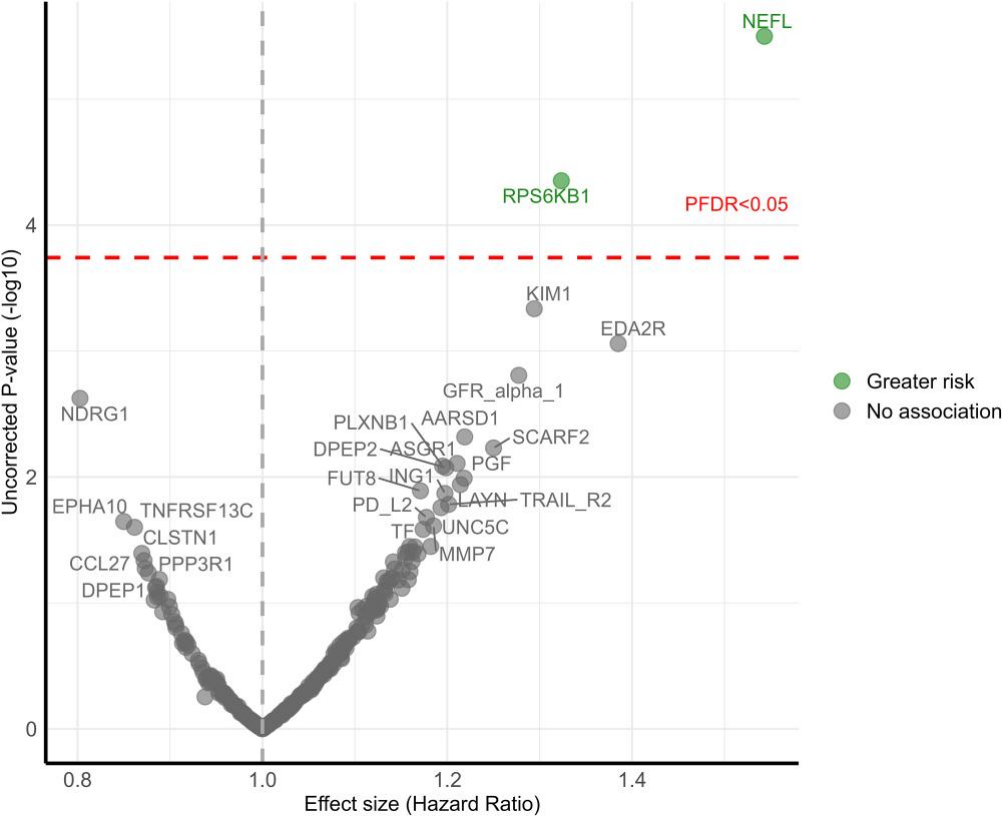
**Volcano plot shows the hazard ratio (*x*-axis) and two-sided *P* values (*y*-axis) for the association of protein concentration with incident all-cause dementia using imputed data.** X-axis displays the hazard ratios from Cox proportional hazard regression models adjusted for age, sex, education, ethnicity, smoking status, depression, cardiovascular disease, body mass index, systolic blood pressure, low-density lipoprotein (LDL) cholesterol, in a sample size of 3249. Y-axis displays the nominal uncorrected *P*-value (−log_10_). Proteins above the horizontal dotted red line were significantly associated with incident all-cause dementia FDR-corrected *P*-value < 0.05.

In the fully adjusted models (adjusted for age, sex, ethnicity, education, smoking status, depression, presence of cardiovascular diseases, body mass index, systolic blood pressure, low-density lipoprotein cholesterol), NEFL [*P_FDR_* = 0.0008; HR (95% CI): 1.54 (1.29, 1.84)] and RPS6KB1 [*P_FDR_* = 0.01; HR (95% CI): 1.33 (1.16, 1.52)] remained significantly associated with ACD (Fig. 1).

Using multiple random forest regression as an alternative data imputation method produced broadly similar results, with RPS6KB1 [*P_FDR_* = 0.01; HR (95% CI): 1.28 (1.14, 1.44)], NEFL [*P_FDR_* = 0.02; HR (95% CI): 1.31 (1.15, 1.49)], and KIM1 [*P_FDR_* = 0.039; HR (95% CI): 1.28 (1.13, 1.45)] demonstrating robust associations with ACD (Supplementary Fig. 8).

There was evidence supporting a sex difference in RPS6KB1 (*P* for interaction = 0.037), with higher level of RPS6KB1 was associated with a greater risk of dementia in men [HR (95% CI): 1.54 (1.24, 1.91)], but the effect was smaller in women [HR (95% CI): 1.21 (1.02, 1.43)]. There was no evidence of interaction effects by age, age^2^, and between age^2^ and sex.

Sensitivity analyses demonstrated the robustness of the association between NEFL and ACD, with significance persisting after excluding participants in other ethnic groups [*P_FDR_* = 0.001; HR (95% CI): 1.53 (1.28, 1.83); Supplementary Fig. 9), APOE ε4 carriers [*P_FDR_* = 0.009; HR (95% CI): 1.61 (1.29, 2.01); Supplementary Fig. 10], cases of dementia occurring within the first year of follow-up [*P_FDR_* = 0.005; HR (95% CI): 1.50 (1.25, 1.80); Supplementary Fig. 11), participants aged <60 years [*P_FDR_* = 0.011; HR([95% CI): 1.47 (1.22, 1.78); Supplementary Fig. 12], and when death was considered as a competing risk in Fine-Gray regression models [*P_FDR_* = 0.002; HR (95% CI): 1.50 (1.26, 1.80); Supplementary Fig. 13]. Similarly, RPS6KB1 exhibited a robust association with ACD, which remained significant after excluding other ethnic groups [*P_FDR_* = 0.004; HR (95% CI): 1.34 (1.18, 1.53)], cases of dementia occurring within the first year of follow-up [*P_FDR_* = 0.025; HR (95% CI): 1.31 (1.15, 1.50)], participants aged <60 years [*P_FDR_* = 0.031; HR (95% CI): 1.31 (1.14, 1.50)], and it was significantly associated with ACD in the competing risk model [*P_FDR_* = 0.014; HR (95% CI): 1.33 (1.16, 1.52)]. However, the significance in association between RPS6KB1 and ACD based on *P*_FDR_ attenuated after excluding APOE ε4 carriers [*P_FDR_* = 0.961; HR (95% CI): 1.28 (1.08, 1.50)]. After excluding other ethnic groups, KIM1 was additionally associated with ACD [*P_FDR_* = 0.027; HR (95% CI): 1.32 (1.15, 1.53)]. When assessed by dementia subtypes, after full adjustment, no protein was found to be significantly associated with Alzheimer’s disease indicated by *P_FDR_* < 0.05 (Supplementary Fig. 14). MMP12 was found to be associated with VAD [*P_FDR_* = 0.046; HR (95% CI): 2.06 (1.41, 2.99); Supplementary Fig. 15). Albeit being non-significant after FDR correction, of the proteins significantly associated with ACD, based on uncorrected nominal statistical significance (denoted as *P_uncorrected_* < 0.05), RPS6KB1 was associated with Alzheimer’s disease [*P_uncorrected_* = 0.006; HR (95% CI): 1.29 (1.07, 1.55)]; and NEFL was associated with VAD [*P_uncorrected_* = 0.001; HR (95% CI): 1.98 (1.31, 2.99)].

**Figure 2:**
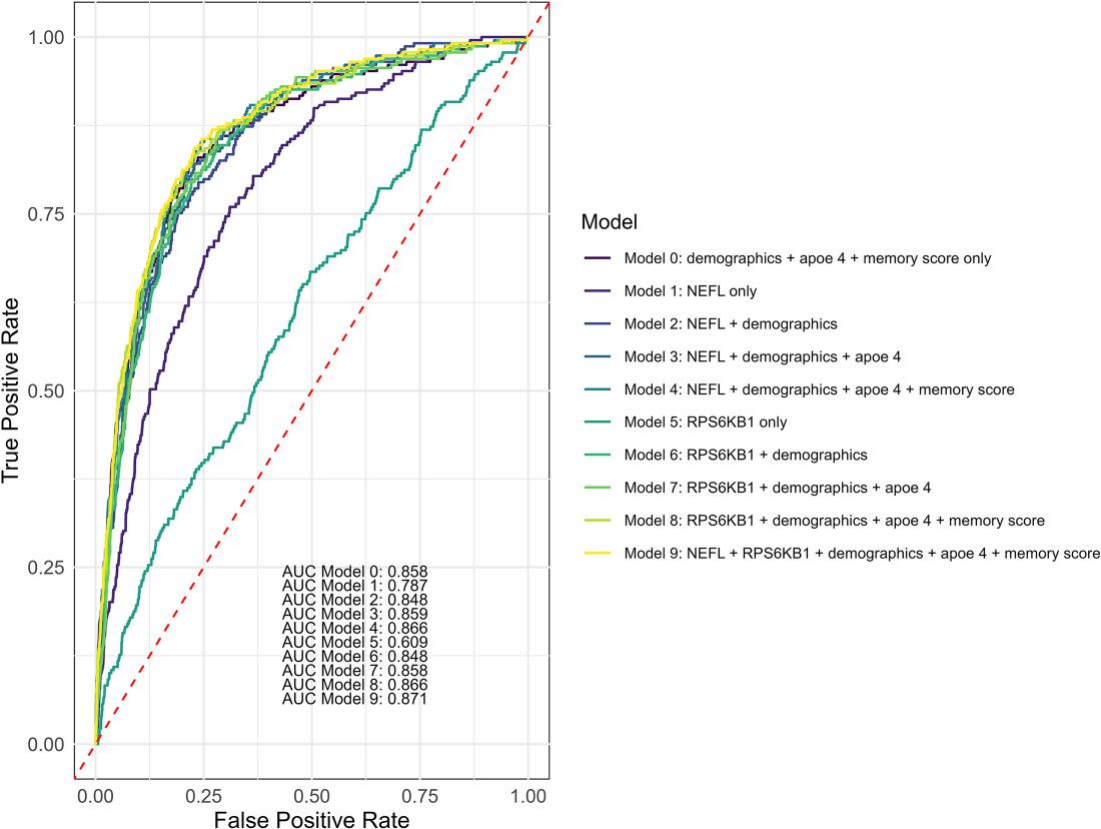
**Predictive accuracy of NEFL and RPS6KB1, alone or in combination with demographic variables, apolipoprotein E 4 (APOE 4) status and memory score for all-cause dementia.** Area under the curve (AUC) of the receiver operating characteristic (ROC) curves illustrate the performance of various variable models in predicting the incidence of all-cause dementia in a sample size of 3249. Demographics variables included sex, age, education and ethnicity. Memory score included a combined test score of immediate recall and delayed recall.

For predicting incident ACD, plasma NEFL and RPS6KB1 parsimonious models yielded modest area under the receiver operating characteristic curve (AUC) values (95% CI) of 0.787 (0.757, 0.815) and 0.609 (0.571, 0.647), respectively (Fig. 2). We also evaluated the performance of these two proteins in combination with other measures, including demographic predictors (age, sex, ethnicity, education), APOE ε4 status, and memory score. When NEFL was combined with these predictors, the model achieved an accuracy of AUC (95% CI): 0.866 (0.840, 0.888). Comparatively, when RPS6KB1 was combined with other predictors, the model achieved a comparable accuracy of AUC (95% CI): 0.866 (0.842, 0.891). NEFL and RPS6KB1 in combination with all the other predictors yielded AUC (95% CI): 0.871 (0.845, 0.894).

**Figure 3:**
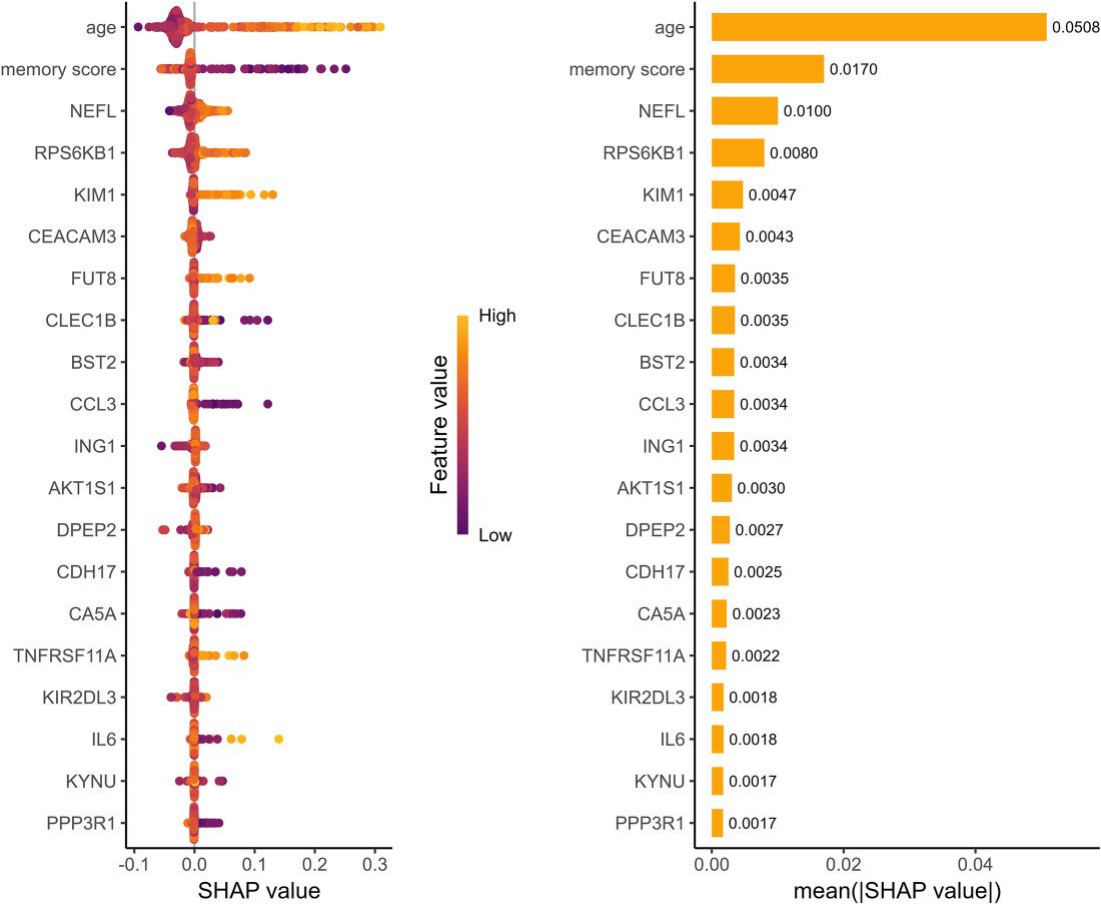
**Protein importance ranking using XGBoost decision tree-based machine learning algorithm and SHAP visualization for selected features on all-cause dementia.** (**A**) SHapley Additive exPlanations (SHAP) values from eXtreme Gradient Boosting (XGBoost) model displaying the top 20 selected features in a sample size of 3249. The *y*-axis indicates the feature names in order of importance ranked from top to bottom. The *x*-axis represents the SHAP value, which indicates the degree of change in log odds. The width of the range of the horizontal bars showed the extent of the contribution to the prediction of all-cause dementia. The colour of each point on the graph represents the value of the corresponding feature. The direction on the *x*-axis indicates the likelihood of developing all-cause dementia towards the right, and likelihood of free from dementia towards the left. (**B**) Mean absolute SHAP values for the top 20 selected features derived from XGBoost model in a sample size of 3249.

For ACD, the XGBoost models revealed that age (mean |SHAP|=0.0508) and memory score (mean |SHAP|=0.0170) were the most important features contributing to the prediction ACD onset. Additionally, protein markers NEFL (mean |SHAP|=0.010), RPS6KB1 (mean |SHAP|=0.0080) and KIM1 (mean |SHAP|=0.047) emerged as the most prominent protein marker in predicting ACD (Fig. 3). The SHAP plot also illustrated that, as an example, individuals with elevated levels of RPS6KB1 were more predisposed to developing ACD, while those with lower levels were more likely to remain ACD-free.

### Analysis 2: protein-dementia associations in the UKB validation cohort

Based on the main, sensitivity and subtype analyses results in ELSA, all identified proteins were selected for validation using the UKB cohort. However, RPS6KB1 was not assayed in the UKB.

In the UKB, which included 52 745 participants with proteomics assayed and without dementia at study baseline (53.9% women, 93.3% white ethnicity), the mean age was 56.8 years (*SD* = 8.2) (Supplementary Table 2). UKB participants with proteomics assayed were, on average, younger than participants in ELSA. Over a median of 13.7 years (min–max: 0.03–16.8 years) of follow-up, a total of 1506 incident ACD, 732 Ad, 281 VAD and 111 FTD cases were recorded.

**Figure 4:**
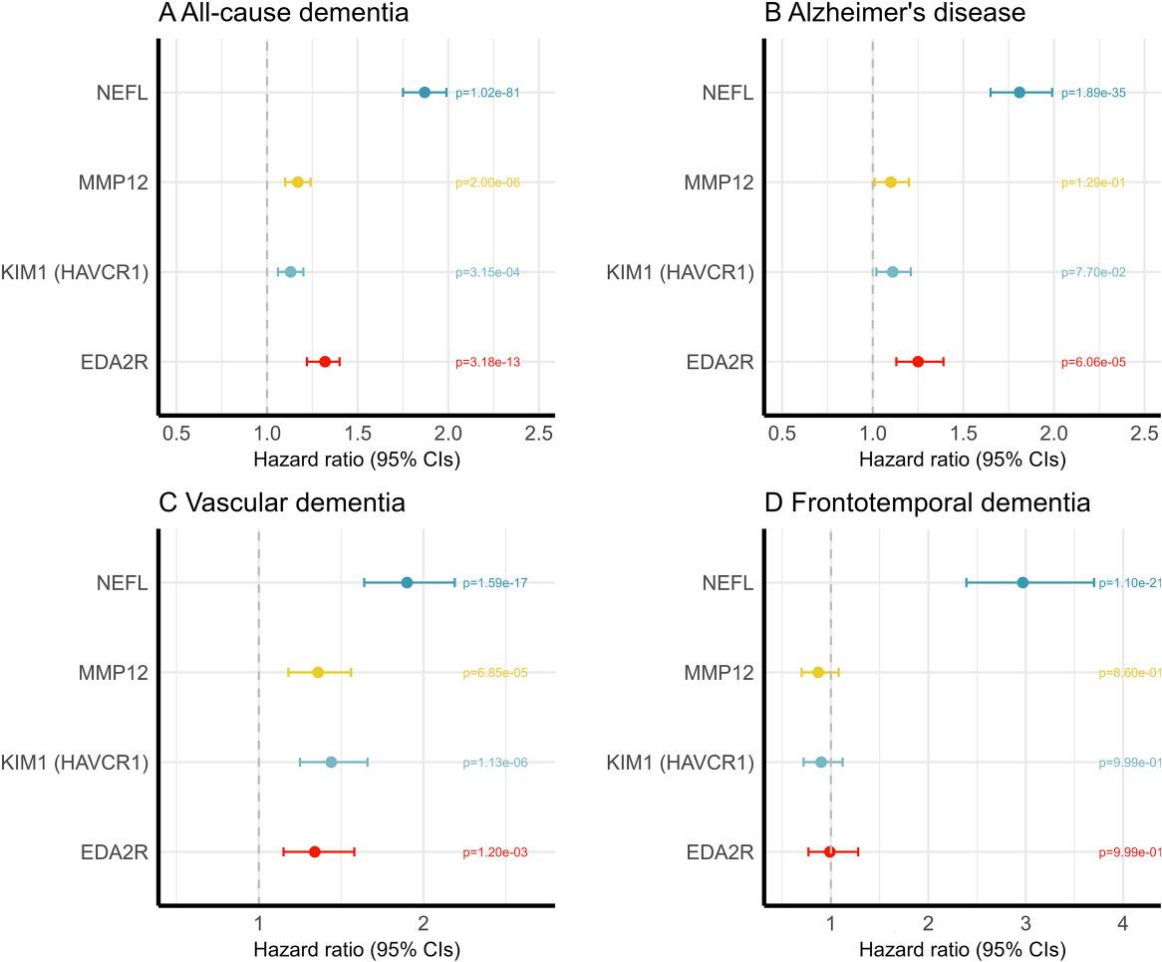
**Forest plots for the associations between identified proteins from ELSA and dementia and dementia subtypes validated in the UK biobank.** Multiple adjusted hazard ratios and 95% confidence intervals (95% CIs) from Cox Proportional Hazard Regression models for NEFL, KIM1 (HAVCR1), MMP12, EDA2R and the associations with: (**A**) all-cause dementia; (**B**) Alzheimer’s disease; (**C**) vascular dementia; (**D**) frontotemporal dementia. All models adjusted for age, sex, education, ethnicity, smoking status, depression, cardiovascular disease, body mass index, systolic blood pressure, low-density lipoprotein (LDL) cholesterol, in a sample size of 52 745. *P* values were FDR corrected.

Using the same adjustment strategy for the Cox regression models in ELSA, NEFL was replicated in the UKB for ACD [*P_FDR_* = 1.02 × 10^−81^; HR (95% CI): 1.87 (1.75, 1.99)], Alzheimer’s disease [*P_FDR_* = 1.89 × 10^−35^; HR (95% CI): 1.81 (1.65, 1.99)], VAD [*P_FDR_* = 1.59 × 10^−17^; HR (95% CI): 1.90 (1.64, 2.19)] and FTD [*P_FDR_* = 1.10 × 10^−21^; HR (95% CI): 2.97 (2.30, 3.70); Fig. 4; Supplementary Table 3). KIM1 was replicated for ACD [*P_FDR_* = 3.15 × 10^−4^; HR (95% CI): 1.13 (1.06, 1.20)], Alzheimer’s disease [*P_FDR_* = 0.077; HR (95% CI): 1.11 (1.02, 1.21)] and VAD [*P_FDR_* = 1.13 × 10^−6^; HR (95% CI): 1.44 (1.25, 1.66)]. MMP12 was replicated for VAD [*P_FDR_* = 6.85 × 10^−5^; HR (95% CI): 1.36 (1.18, 1.56)], and it was also associated with ACD [*P_FDR_* = 2.00 × 10^−6^; HR (95% CI): 1.17 (1.10, 1.24)]. EDA2R was replicated for ACD [*P_FDR_* = 3.18 × 10^−13^; HR (95% CI): 1.31 (1.22, 1.40)], Alzheimer’s disease [*P_FDR_* = 6.06 × 10^−5^; HR (95% CI): 1.25 (1.13, 1.39)] and VAD [*P_FDR_* = 0.001; HR (95% CI): 1.34 (1.15, 1.58)].

### Analysis 3: two-sample bi-directional MR

For two-sample bi-directional MR analyses, since RPS6KB1 was not assayed in the UKB, and there were insufficient instruments based on the genome-wide association meta-analysis the SCALLOP Consortium, MR analyses were conducted for NEFL, KIM1, EDA2R and MMP12.

For GWAS used for dementia, three GWAS for Alzheimer’s disease (denoted as Kunkle 2019, Bellenguez 2022, FinnGen 2023), one GWAS for ACD (FinnGen 2023) and one GWAS for VAD (FinnGen 2023) were used.

In the forward direction MR (circulating protein concentration → dementia; Supplementary Table 4; Supplementary Figs 16–19), there was evidence of a potential causal link from circulating EDA2R to Alzheimer’s disease (FinnGen 2023; coefficient [β]; standard error [se]: 0.259 [0.096]; *P* = 0.007, based on the inverse-variance weighted [IVW] method), and ACD [β (se): 0.232 (0.110); *P* = 0.035, based on IVW]. However, these findings were less robust when methods such as MR-Egger were applied, suggesting horizontal pleiotropy.

MR analyses in the backward direction (dementia → circulating protein concentration) (Supplementary Table 4; Supplementary Figs 20–23) supported Alzheimer’s disease (Bellenguez 2022) [β (se): 0.056 (0.014); *P* = 1.081 × 10^−4^, based on IVW], Alzheimer’s disease (FinnGen 2023) [β (se): 0.033 (0.015); *P* = 0.024, based on IVW] and VAD [β (se): 0.036 (0.014); *P* = 0.008, based on maximum likelihood] as cause of altered NEFL abundance. Furthermore, there was evidence supporting a causal link between Alzheimer’s disease (Kunkle 2019) and MMP12, demonstrated by MR-Egger, weighted median, and weighted mode methods [β (se): 0.027 (0.012); *P* = 0.043; β (se): 0.025 (0.012); *P* = 0.037, and β (se): 0.026 (0.012); *P* = 0.037, respectively]. There was evidence suggesting that Alzheimer’s disease (FinnGen 2023) [β (se): 0.013 (0.049); *P* = 0.007 (based on IVW)], ACD [β (se): −0.032 (0.012); *P* = 0.007 (based on IVW)] and VAD [β (se): −0.036 (0.012); *P* = 0.003 (based on weighted median)] might have a causal link to altered EDA2R abundance.

### Analysis 4: two sample drug target MR (cis-MR)

The instrument selection for drug target MR (cis-MR) relies on single nucleotide polymorphisms (SNPs) within or near the gene encoding region that regulates the protein of interest. However, the encoding region of EDA2R is located within the X chromosome, which precluded the analysis of drug target MR on EDA2R, as the sex chromosomes were excluded from GWAS summary statistics. There were insufficient valid instruments for RPS6KB1. Cis-MR was conducted for NEFL, KIM1 and MMP12.

In drug target MR, there was no causal evidence for any of the protein–dementia relationships (Supplementary Table 5, Supplementary Figs 24–26).

In sensitivity analysis using a less stringent instrument selection approach, results were largely consistent. There was some evidence indicating a causal relationship between KIM1 and Alzheimer’s disease (FinnGen 2023) [β (se): −0.102 (0.041); *P* = 0.037] and ACD [and β (se): −0.094 (0.037); *P* = 0.036] based on MR-Egger.

#### Analysis 5: enrichment analysis

**Figure 5:**
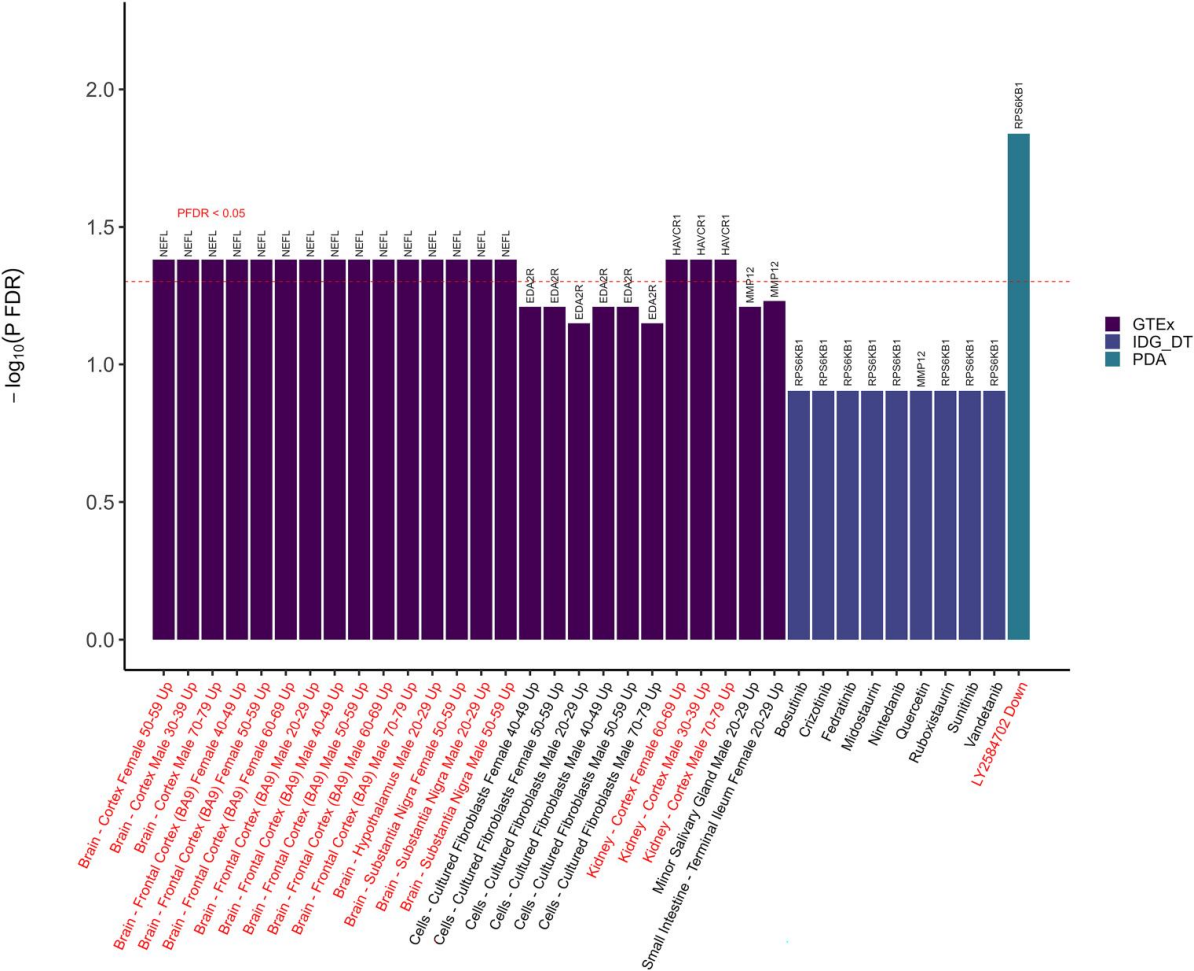
**Enrichment analysis of the identified proteins in genotype-tissue expression (GTEx) 2023, illuminating the druggable genome (IDG) drug target 2022, and proteomics drug atlas (PDA) 2023.** Enrichment for Genotype-Tissue Expression (GTEx) 2023, Illuminating the Druggable Genome (IDG) drug target 2022, and Proteomics Drug Atlas (PDA) 2023. Significant proteins after FDR correction (denoted as *P*_FDR_) derived from Cox proportional hazard regressions in minimally- and fully adjusted models were fed into Enrichr (https://maayanlab.cloud/enrichr/) for enrichment analysis. The full list of proteins from ELSA was used as the background gene set. Terms above the horizontal dotted line were enriched after FDR-correction with *P*-value < 0.05, and the texts were highlighted in red.

**Figure 6:**
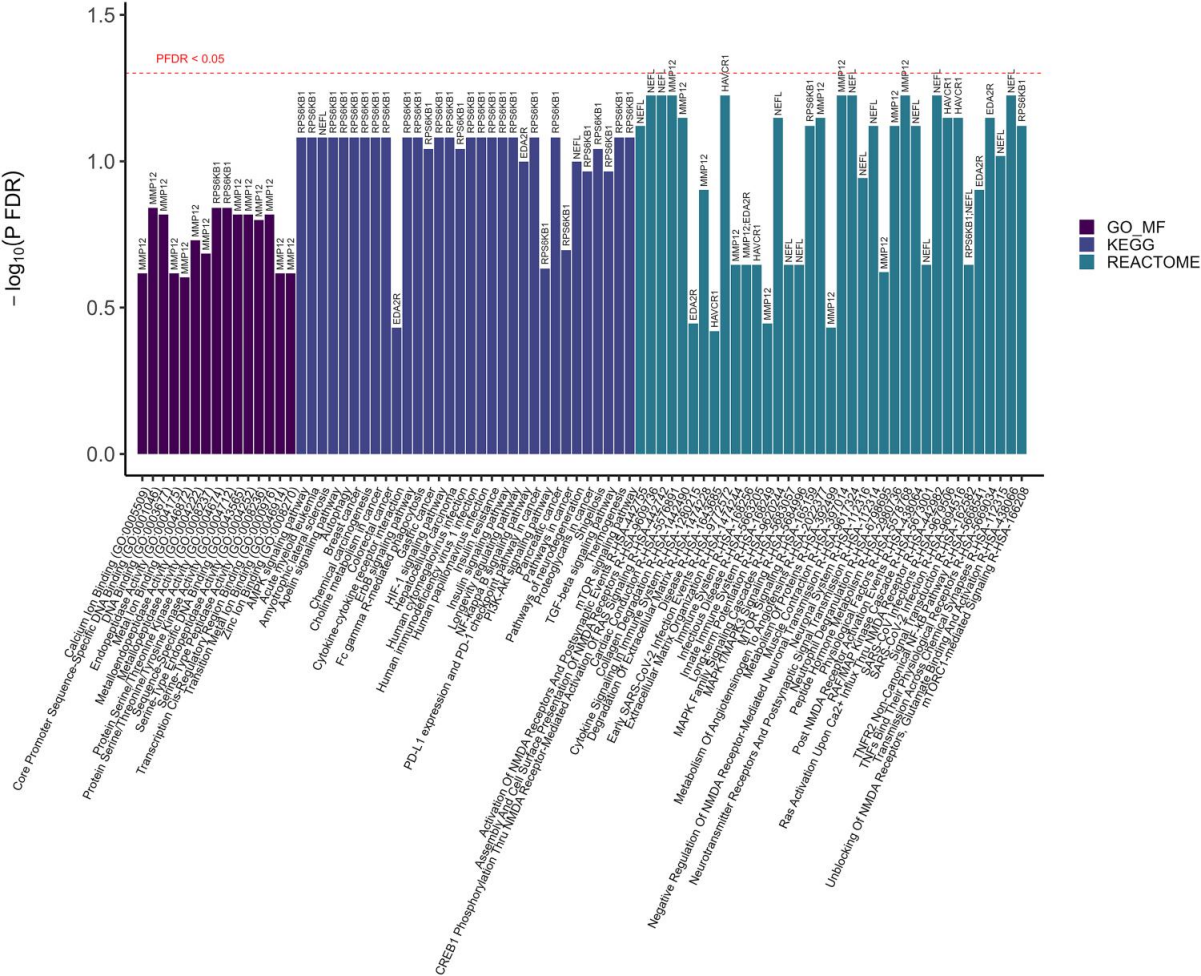
**Enrichment analysis of the functional annotations in identified proteins in gene ontology (GO) 2023, Kyoto encyclopaedia of genes and genomes (KEGG) 2021, and reactome pathways 2022.** Enrichment for gene ontology (GO) 2023 (GO_MF: Gene Ontology Molecular function), Kyoto Encyclopaedia of Genes and Genomes (KEGG) 2021, and Reactome pathways 2022. Significant proteins after FDR correction (denoted as P_FDR_) derived from Cox proportional hazard regressions in minimally- and fully adjusted models were fed into Enrichr (https://maayanlab.cloud/enrichr/) for enrichment analysis. The full list of proteins from ELSA was used as the background gene set. Terms above the horizontal dotted line were enriched after FDR-correction with *P*-value < 0.05, and the text were highlighted in red.

In Figs 5 and 6 (also depicted in Supplementary Table 6), the enrichment analyses revealed several biological pathways potentially implicated for the identified proteins (NEFL, RPS6KB1, KIM1, EDA2R and MMP12), including the immune system, cancers and insulin signaling. Tissue expression analysis showed expression in the brain for NEFL and in the kidney for KIM1. Notably, one drug, LY2584702, which is a selective, adenosine triphosphate (ATP)-competitive p70S6 K inhibitor, has been investigated in clinical trials for the treatment of renal cell carcinoma, metastases, neoplasm, and neuroendocrine tumors—where RPS6KB1 was shown to be implicated in the mechanisms of action of the drug.

Upon searching the Open Targets platform, we identified 10 known small molecule drugs in clinical trials (including LY2584702) that are linked to two proteins (RPS6KB1 and MMP12), targeting various cancers, chronic hepatitis C infection and chronic obstructive pulmonary disease (Supplementary Table 7).

## Discussion

Through a broad proteomics study within the ELSA cohort, encompassing 276 proteins across 3249 participants, we identified key proteins linked to an elevated risk of incident ACD (NEFL, RPS6KB) and VAD (MMP12), based on fully adjusted models. NEFL and RPS6KB1 individually displayed moderate predictive accuracy for ACD risk (AUC = 0.787 and 0.609, respectively) and yielded an AUC of 0.871 when combined with demographic, genetic and cognitive factors. Notably, XGboost machine algorithm further underscored RPS6KB1, NEFL and KIM1 as the most important protein features in predicting ACD onset. Further, there was evidence for sex difference in RPS6KB1 in relation to ACD risk, exhibiting a stronger association in men. These discoveries from ELSA were robustly replicated in the UKB, where NEFL, MMP12, KIM1 and EDA2R were significantly associated with ACD, Alzheimer’s disease and VAD. Employing MR approaches, several causal relationships were observed between Alzheimer’s disease and VAD with NEFL, Alzheimer’s disease with MMP12, and between Alzheimer’s disease, ACD, VAD, with EDA2R in the reverse direction. There was no evidence supporting causal relationships between proteins and dementia from cis-MR analyses.

To the best of our knowledge, this is the first study to identify an association between RPS6KB1 and the risk of dementia. RPS6KB1 functions as a serine/threonine-protein kinase, operating downstream of phosphoinositide 3-kinase (PI3 K)/mammalian target of rapamycin (mTOR) signaling in response to growth factors and nutrients, promoting cell proliferation, growth and progression through the cell cycle.^47^ The mTOR complex 1 (mTORC1) signaling was found in a previous study to be implicated in the biological aging process,^48^ such that the inhibition of mTOR may extend lifespan given that the mTOR activity becomes abnormally high with age.^48^ In the nervous system, the mTOR pathway is implicated in the regulation of synaptic remodeling and long term potentiation.^49-52^ Importantly, mTOR plays a crucial role in autophagy regulation in neurons,^52,53^ and the mTOR/p70S6K axis is shown to be essential in the early phases of plasticity for synaptic modifications and the formation of enduring memory.^54^ Previous analyses of the ARIC study similarly highlighted the importance of autophagy signaling pathways in the two decades before dementia onset.^10^ Interestingly, our sensitivity analysis, which excluded APOE ε4 carriers observed an attenuation in association between RPS6KB1 and dementia after considering multiple testing. Previous literature highlighted the mechanisms affected by APOE ε4,^55^ such that the presence of APOE ε4 may be necessary for the overactivation in mTOR pathway which subsequently lead to tau hyperphosphorylation and reduced Aβ clearance.^56^ Nevertheless, genomics studies found that the combined effect of alleles in the RPS6KB1 gene, along with other genes in the tau kinase pathway, is linked to an increased risk of late-onset Alzheimer’s disease in without APOE ε4 allele.^57^ At a lower expression level, RPS6KB1 can facilitate the growth of damaged axons resulting from CNS injury.^58^ Transcriptomic exploration has revealed the central role of RPS6KB1 and alterations in its co-expression occur during the initial stages of Alzheimer’s disease, which highlights its potential as a biomarker for the early diagnosis of Alzheimer’s disease.^57^ Several small clinical trials of rapamycin are underway for investigating age-related diseases including Alzheimer’s disease,^48^ with primary outcomes assessing the effects on cognitive performance, and biomarkers of aging.^48^ From the Proteomics Drug Atlas, one drug (LY2584702) was found to target RPS6KB1, which is a highly selective adenosine triphosphate competitive inhibitor against p70S6 Kinase. Further explorations are therefore needed to decipher the relationship between RPS6KB1 and the protein’s pharmacological properties. Moreover, sex-specific association between RPS6KB1 and ACD was intriguing. Animal studies have shown that the genetic deletion of RPS6KB1 inhibits cellular senescence and promotes longevity, but only in female mice.^59^ Additionally, evidence suggests that RPS6KB1 mRNA expression varies based on menopausal status,^60^ pointing to complex molecular interactions with hormones. The increased dementia risk that RPS6KB1 poses for men, however, warrants further investigation.

Elevated NEFLs were found to be associated with an increased risk of ACD in ELSA, and with ACD, Alzheimer’s disease, VAD and FTD in the UKB. Consistently, previous studies from the UKB ranked NEFL as the most important protein associated with future dementia events out of 1463 protein markers^16^ and was associated Alzheimer’s disease and VAD.^17^ NEFL is a marker of axonal injury^61,62^ and is implicated in several biological mechanisms related to dementia,^63,64^ including neurodegeneration,^65,66^ inflammation,^67^ central nervous system (CNS) injury^68-70^ and atherosclerosis.^71^ It is a well-established and non-specific marker of neurodegenerative diseases. While NEFL was found in our study to be causally linked to dementia based on the MR findings, the strongest indication was in the backwards direction, which points toward its role as a manifestation of prodromal dementia and anomalies in the brain, rather than a cause of dementia. This underscores the value of NEFL as an important diagnostic and early identification marker, as also demonstrated by the prediction models. It should be noted that the inconsistencies in MR findings from the current study across various Alzheimer’s disease GWAS could stem from the fact that the chosen genetic variant serving as the instrumental variable might exert a varied impact on the outcome within the represented population.

Matrix metalloproteinases (MMPs) belong to a multigenic family of membrane-bound or secreted zinc-containing endopeptidases, which indirectly modulate the cellular processes through activation and inactivation of signaling molecules such as trophic factors cytokines, and receptor.^72,73^ MMPs play important roles in cell proliferation and death, neuroinflammation, neurodegeneration and glial reactivity^74^ and are linked to their proteolytic disruption action on the blood-brain barrier.^75^ More specifically, MMP2, MMP3 and MMP9 were shown to play a crucial role in Alzheimer’s disease,^76-78^ and damage to the white matter associated with VAD.^79^ Based on experimental models, Aβ_40_ contribute to the changes in blood-brain barrier (BBB) permeability, and increased expression of MMPs in transgenic human amyloid precursor protein (hAPP)-overexpressing mice, in turn compromises BBB integrity.^80^ Selective inhibitors for MMP12^81^ was shown to reduce inflammation and delay of atherosclerosis progression.^82,83^ For dementia, the associations between MMP12 and VAD risk in the UKB, and MMP12 and Alzheimer’s disease risk in the ARIC cohort was similarly highlighted.^10,16^ At an elevated dosage, this medication can penetrate the BBB and manifest an inhibitory effect on metalloproteinase activity within the brain,^84^ and was shown to decrease some seizure-related parameters.^84^

In our study, although the significant findings for KIM1 and EDA2R attenuated after full adjustments, MR analyses showed some possible causal links between these proteins and dementia. However, some of these MR results may be biased by horizontal pleiotropy with the proteins affecting multiple diseases,^85^ possibly via immune, renal and metabolic disease pathways,^85-87^ which subsequently contribute to the risk of dementia.^85^ There was also evidence from previous studies indicating higher levels of EDA2R were associated a smaller total brain volume, smaller grey matter volume, and less normal-appearing white matter volume.^86^

The current study exhibits robustness through several key strengths. Firstly, it draws upon two extensive population-based cohorts with prolonged follow-up, employing high-throughput and reliable proteomics data. The selection of the protein panel in ELSA is noteworthy for its focused curation on dementia-related markers, enhancing the study's precision in investigating associations with dementia risk in a nationally representative sample of older adults. The findings through the inclusion of the UKB encompassing a broader selection of proteins enhance the validity of our results. Both cohorts are well-characterized longitudinal cohorts, which enabled adjustment for wide range of factors. Furthermore, our study benefits from applying a robust and comprehensive dementia algorithm in ELSA, which integrated information from various sources, bolstered by details on medication use and informant-solicited information, which has been reported to correlates better with objective cognitive performance than self-report alone as well as medication which particularly captured those with younger onset dementia,^88^ allowing for accurate identification of incident dementia cases and the exclusion of prevalent cases. Another significant strength lies in our approach to assessing protein-dementia associations through a range of established methods for evidence triangulation. Additionally, the utilization of Olink antibody-based proximity extension assay is recognized for its superior specificity in proteomics assays.^19^ The integration of proteogenomic in MR analyses was an additional strength.^12^

Some limitations should be acknowledged. First, several circulating protein markers potentially relevant to dementia, for example, GFAP and GDF-15, were not assayed in ELSA. There is also limited specification, or a lack of protein measurements from the A/T/N classification framework,^89^ such as beta-amyloid, p-tau217 and p-tau181. Third, another limitation is that the algorithm used for dementia ascertainment lacked information from primary care data and pathology for confirming dementia cases, thus uncertainties exist ascertaining dementia subtypes. Nevertheless, it is important to note that Alzheimer’s disease and VAD pathology often co-exist on a population level, and many dementia patients exhibit mixed neuropathology.^90^ Fourth, it is important to note that we lacked external validation cohorts for RPS6KB1. Lastly, there are inherent assumptions in MR analyses, and for drug target MR specifically, genetics might not directly inform on specific pharmacological aspects of drug exposure.

In conclusion, our proteomics analysis from two large-scale, population-based cohorts in the UK highlighted the utility of proteomics in identifying novel targets, enhancing our understanding of the mechanisms underlying dementia. MR analyses leveraging extensive GWAS data substantiated some of these protein-dementia relationships with causal evidence. Looking forward, integrating large-scale population-based proteomics with other omics, such as genomics,^12^ offers potential for deeper biological insights into diseases. Further research and validation are required to clarify the role of RPS6KB1 in ADRD and to explore potential sex differences in its effects.

## Supplementary Material

fcaf097_Supplementary_Data

## Data Availability

The ELSA data is available on the UK Data Service. The proteomics data in ELSA will be deposited on the UK Data Service upon publication. All data from the UK Biobank, including the proteomics data, is available by directly submitting a project application to the UK Biobank. All GWAS summary statistics are available online: https://doi.org/10.7303/syn51364943; https://www.finngen.fi; https://gwas.mrcieu.ac.uk/. The codes used for all analyses are available on GitHub repository: https://github.com/jgong94/ELSA_proteomics_dementia.
