# Supplementary material for: Unraveling the role of proteins in dementia: insights from two UK cohorts with causal evidence
Source: Brain Commun. 2025 Mar 3;7(2):fcaf097. doi: 10.1093/braincomms/fcaf097 (PMC11906402; doi:10.1093/braincomms/fcaf097)
Supplement: fcaf097_Supplementary_Data [file fcaf097_supplementary_data.docx]

**Supplementary Materials**

**Content**

**Supplementary Methods**

| ELSA proteomics data quality control pipeline. | Page 3-4 |
| --- | --- |
| Dementia algorithm development in ELSA. | Page 5-7 |
| Two-sample bidirectional Mendelian randomization (MR) and cis-MR. | Page 8-9 |
| References for supplementary methods. | Page 10 |

**Supplementary Tables**

| Supplementary Table 1A. International classification of disease codes for definite dementia as recorded in hospital admission and mortality records in ELSA. | Page 11 |
| --- | --- |
| Supplementary Table 1B. International classification of disease codes by dementia sub-type for definite dementia cases in ELSA. | Page 12 |
| Supplementary Table 2. Demographic and phenotypic information of the ELSA and UK Biobank participants. | Page 13-14 |
| Supplementary Table 3. Multiple-adjusted hazard ratios and 95% confidence intervals for the associations between identified proteins and dementia outcomes in the UK Biobank. | Page 15 |
| Supplementary Table 4. Two sample bi-directional Mendelian randomization between proteins and dementia. | Page 16-19 |
| Supplementary Table 5. Two sample cis-Mendelian randomization between proteins and dementia. | Page 20-22 |
| Supplementary Table 6. Enrichment analysis for the identified proteins. | Page 23-26 |
| Supplementary Table 7. Drugs linked to the identified proteins associated with dementia, drug type, their potential mechanisms of action, current target disease, and the status and phase of the trials. | Page 27 |
| Supplementary Table 8. Summary information of dementia GWAS datasets. | Page 28 |

**Supplementary Figures**

| Supplementary Figure 1. Flow diagram for ELSA proteomics project sample selection and quality control pipeline. | Page 29 |
| --- | --- |
| Supplementary Figure 2. Dementia cases by data source in ELSA. | Page 30 |
| Supplementary Figure 3. Boxplot for all protein concentration from the Olink Target 96 Cardiovascular II panel in normalized protein expression (NPX) in ELSA by dementia status. | Page 31 |
| Supplementary Figure 4. Boxplot for all protein concentration from the Olink Target 96 Neurology I panel in normalized protein expression (NPX) in ELSA by dementia status. | Page 32 |
| Supplementary Figure 5. Boxplot for all protein concentration from the Olink Target 96 Neuro Exploratory panel in normalized protein expression (NPX) in ELSA by dementia status. | Page 33 |
| Supplementary Figure 6. Volcano plot showing the unadjusted HR (x axis) and two-sided P values (y axis) for the association between protein concentration with incident all-cause dementia using imputed data. | Page 34 |
| Supplementary Figure 7. Volcano plot shows the sex-, age- and ethnicity-adjusted HR (x axis) and two-sided P values (y axis) for the association between protein concentration with incident all-cause dementia using imputed data. | Page 35 |
| Supplementary Figure 8. Volcano plot shows the fully adjusted HR (x axis) and two-sided P values (y axis) for the association between protein concentration with incident all-cause dementia using imputed data from random forest methods. | Page 36 |
| Supplementary Figure 9. Volcano plot shows the fully adjusted HR (x axis) and two-sided P values (y axis) for the association between protein concentration with incident all-cause dementia, excluding other ethnic groups using imputed data. | Page 37 |
| Supplementary Figure 10. Volcano plot shows the fully adjusted HR (x axis) and two-sided P values (y axis) for the association between protein concentration with incident all-cause dementia, excluding APOE 4 carriers using imputed data. | Page 38 |
| Supplementary Figure 11. Volcano plot shows the fully adjusted HR (x axis) and two-sided P values (y axis) for the association between protein concentration with incident all-cause dementia, after reducing the possibility of reverse causation bias by excluding all-cause dementia cases that occurred during the first year of follow-up using imputed data. | Page 39 |
| Supplementary Figure 12. Volcano plot shows the fully adjusted HR (x axis) and two-sided P values (y axis) for the association between protein concentration with incident all-cause dementia, excluding participants <60 years using imputed data. | Page 40 |
| Supplementary Figure 13. Volcano plot shows the fully adjusted sub-distribution HR (x axis) and two-sided P values (y axis) for the association between protein concentration with incident all-cause dementia, using Fine-Gray competing risk regression using imputed data. | Page 41 |
| Supplementary Figure 14. Volcano plot showing the fully adjusted HR (x axis) and two-sided P values (y axis) for the association between protein concentration with incident Alzheimer’s disease using imputed data. | Page 42 |
| Supplementary Figure 15. Volcano plot shows the fully adjusted HR (x axis) and two-sided P values (y axis) for the association between protein concentration with incident vascular dementia using imputed data. | Page 43 |
| Supplementary Figure 16. Two-sample Mendelian randomization in the forward direction (protein concentration 🡪 dementia) scatter plots for NEFL in five GWAS for Alzheimer’s disease, all-cause dementia, and vascular dementia. | Page 44 |
| Supplementary Figure 17. Two-sample Mendelian randomization in the forward direction (protein concentration 🡪 dementia) scatter plots for KIM1 (HAVCR1) in five GWAS for Alzheimer’s disease, all-cause dementia, and vascular dementia. | Page 45 |
| Supplementary Figure 18. Two-sample Mendelian randomization in the forward direction (protein concentration 🡪 dementia) scatter plots for EDA2R in five GWAS for Alzheimer’s disease, all-cause dementia, and vascular dementia. | Page 46 |
| Supplementary Figure 19. Two-sample Mendelian randomization in the forward direction (protein concentration 🡪 dementia) scatter plots for MMP12 in five GWAS for Alzheimer’s disease, all-cause dementia, and vascular dementia. | Page 47 |
| Supplementary Figure 20. Two-sample Mendelian randomization in the reverse direction (dementia 🡪 protein concentration) scatter plots for NEFL in five GWAS for Alzheimer’s disease, all-cause dementia, and vascular dementia. | Page 48 |
| Supplementary Figure 21. Two-sample Mendelian randomization in the reverse direction (dementia 🡪 protein concentration) scatter plots for KIM1 (HAVCR1) in five GWAS for Alzheimer’s disease, all-cause dementia, and vascular dementia. | Page 49 |
| Supplementary Figure 22. Two-sample Mendelian randomization in the reverse direction (dementia 🡪 protein concentration) scatter plots for EDA2R in five GWAS for Alzheimer’s disease, all-cause dementia, and vascular dementia. | Page 50 |
| Supplementary Figure 23. Two-sample Mendelian randomization in the reverse direction (dementia 🡪 protein concentration) scatter plots for MMP12 in five GWAS for Alzheimer’s disease, all-cause dementia, and vascular dementia. | Page 51 |
| Supplementary Figure 24. Two-sample drug target Mendelian randomization scatter plots for NEFL in five GWAS for Alzheimer’s disease, all-cause dementia, and vascular dementia. | Page 52 |
| Supplementary Figure 25. Two-sample drug target Mendelian randomization scatter plots for KIM1 (HAVCR1) in five GWAS for Alzheimer’s disease, all-cause dementia, and vascular dementia. | Page 53 |
| Supplementary Figure 26. Two-sample drug target Mendelian randomization scatter plots for MMP12 in five GWAS for Alzheimer’s disease, all-cause dementia, and vascular dementia. | Page 54 |

**Supplementary Methods**

**ELSA proteomics data quality control pipeline**

This section provides a summary of the study design and quality control (QC) protocol implemented by the proteomics data curation within the English Longitudinal Study of Ageing (ELSA) for normalized protein expression (NPX) data generated using the antibody based Olink™ Proximity Extension Assay (PEA) in blood plasma samples derived from 3262 ELSA participants in Wave 4 in 2008.

Data is presented as normalized protein expression (NPX) values, Olink Proteomics’ arbitrary unit on log2 scale. Data points for samples that did not pass QC are written in red text in the raw data files derived from Olink.

The protocol follows the following curation and quality control pipeline:

1. Data pre-processing and internal quality control by Olink

Four internal controls are added to each sample to monitor the quality of assay performance, as well as the quality of individual samples. The QC is performed in two steps:

- Each sample plate is evaluated on the standard deviation (SD) of the internal controls. This should be below 0.2 NPX. Only data from sample plates that pass this quality control will be reported.
- The quality of each sample is assessed by evaluating the deviation from the median value of the controls for each individual sample. Samples that deviate less than 0.3 NPX from the median pass quality control.

1. Importing data and removing Olink control samples
2. Removing data with quality control warnings or assay warnings

Samples that did not pass the QC are indicated in columns named "QC Warning". Data points from samples that do not pass QC should be treated with caution.

1. Outlier sample detection and removal

IQR-median and principal component analyses (PCA) are used to detect and visualize outliers. Generates PCA projection of all samples from NPX data along two principal components (Default PC2 vs PC1) colored by the variable QC_Warning and including the percentage of explained variance. By default, the values scaled and cantered in the PCA and proteins with missing NPX values removed from the corresponding assay(s). Imputation by median value is done for assays with missingness

**Dementia algorithm development in ELSA**

Regarding self-reported diagnosis of dementia, during each interview, participants were asked whether a medical professional had informed them of a diagnosis of AD, dementia, organic brain senility, or any other serious memory condition. A positive response indicates the presence of dementia.

Caregivers completed a modified short-form IQCODE on behalf of individuals unable to respond independently. Caregivers (acting as proxies) were instructed to assess the current functional performance of the participant, comparing it to that of two years prior, instead of the standard 10-year interval. Consistent with prior research, individuals with an IQCODE score exceeding 3.38 were classified as having dementia.[1]

Medication data, encoded for analysis, was collected during nurse visits in waves 6 (2012-2013) and wave 8 (2016-2017), and wave 9 (2018-2019). In waves 8 and 9, two mutually exclusive subsets of the sample underwent nurse visits. As specified in the British National Formulary (BNF), we specifically considered four common drugs for dementia: Donepezil hydrochloride (0411000D0), Galantamine (0411000F0), Memantine hydrochloride (0411000G0), and Rivastigmine (0411000E0).

The 10th revision of the International Statistical Classification of Diseases and Related Health Problems (ICD-10) was employed to extract dementia-related outcomes from the National Health Service (NHS) Hospital Episode Statistics (HES) data; HES data covered the period between March 1997 and January 2018. Mortality statistics provided by the Office for National Statistics (ONS) were accessible for the period between April 2002 and April 2018. The methodology used to determine all-cause dementia (ACD) using mortality-linked data mirrored the approach taken with HES data based on ICD codes.

Different data sources were used for ascertaining dementia subtypes. For AD (Alzheimer’s disease), four data sources were utilized: self-reported physician-diagnosis of AD during each study interview, medication usage based on BNF from nurse visits, and the presence of ICD-10 codes in HES and mortality data. For ascertaining vascular dementia (VAD), ICD-10 codes from HES and mortality data were used.

The term "time to event" pertains to the duration until a specific predefined endpoint of interest occurs. The methodology for determining the time to event in participants with a documented event of dementia varied by the source where the dementia event was initially established. This divergence in assessment methods is summarized as follows:

1. *Interview:* If the earliest date of diagnosed dementia was reported at the time of interview, we calculated the time to event by determining the midpoint between the interview date when doctor-diagnosed dementia was first reported and the date of the preceding interview. Participants who reported diagnosed dementia at the first interview were classified as prevalent cases.
2. *IQCODE:* If the earliest date corresponded to dementia reported through the proxy/informant method using IQCODE data (and considering that the questionnaire solicited information pertaining to the preceding two years), the time to event was computed as one year preceding the interview date where the IQCODE score was calculated as < 3.38.
3. *Hospital Episode Statistics (HES):* When the earliest date of dementia was derived from HES, we used the exact date of the dementia-related admission (HES datasets included appointments at Outpatient and Admitted Patient Care services and attendance at Accident and Emergency units).
4. *Mortality statistics:* In cases where the earliest date of dementia was established through linkage to mortality data, we used the date of death.
5. *Medications*: When the earliest date of dementia was deduced from medication information obtained during the nurse visits, we calculated the time to event by determining the midpoint between the date of the nurse visit when dementia medication usage was recorded and the date of the interview prior to the nurse visit.

For the participants not recorded as having dementia, the date of censoring varied depending on conditions such as the consent given for linkage to hospital episodes and mortality data:

1. If the participant's death was confirmed by mortality data, they were censored at the date of death.
2. If the participant provided consent for mortality statistics linkage (and did not later revoke it) and was not recorded as having died, they were censored on 15^th^ April 2018, the last date of linkage).
3. If the participant consented to HES linkage (and did not later revoke it) and was not recorded as having died, they were censored on 15^th^ January 2018, the last date of linkage.
4. For participants who did not consent to HES or mortality linkage, the date of censoring was set at the date of their last interview.

**Two-sample bidirectional Mendelian randomization (MR) and cis-MR**

MR pertains to employing genetic variants to explore causal connections regarding the influence of modifiable exposures on a range of outcomes. Anchored in Mendel's laws of inheritance and instrumental variable estimation techniques, MR principles facilitate the inference of causal effects, even in the presence of unobserved confounding factors.

The MR approach rests on three key assumptions: 1) the instruments are correlated with the exposure; 2) the instruments are linked to the outcome solely through the studied exposure (exclusion restriction assumption); and 3) the instruments are independent of other factors influencing the outcome (independence assumption).

Selection of instruments for different dementia types was done by leveraging summary statistics from different GWAS. For AD, we used GWAS summary statistics derived from three consortia: (International Genomics of Alzheimer’s Project (IGAP),[2] European Alzheimer & Dementia Biobank (EADB) consortium,[3] and the FinnGen 2023 study);[4] For ACD and VAD GWAS were both derived from the FinnGen 2023 study.[4] Summary details of the dementia GWAS used were included in Supplementary Table 8. To ensure the three MR assumptions were not violated, instruments for the exposure were selected by using association at genome-wide significance (P < 5 × 10^−8^). SNP with a minor allele frequency (MAF) < 5% were excluded. linkage disequilibrium (LD) clumping was done with a window size of 10,000 kilobases [kb] at an R^2^ threshold of 0.001. In cases where a requested SNP from the exposure GWAS is not found in the outcome GWAS, a proxy SNP with a LD coefficient of R^2^ > 0.8 to the requested missing target SNP were sought as a substitute, if available, by querying the LDLink web server. LD proxies are determined using data from the 1000 Genomes European sample. The returned information includes the effect of the proxy SNP on the outcome, along with details such as the proxy SNP itself, the effect allele of the proxy SNP, and the corresponding allele for the target SNP. The effect of a SNP on an outcome and exposure were then harmonized to be relative to the same allele. We specify the inference of positive strand alleles by utilizing allele frequencies for palindromes instead of eliminating palindromic variants. F-statistics were used to assess the SNP-exposure strength (F > 10).

We first performed the MR in the forward direction (circulating protein concentration → dementia) on the harmonized effects to estimate the effect of genetically proxied protein abundance on genetic liability to the dementia outcome of interest to be relative to the same allele. Given the study outcomes were AD and related dementia, variants in the APOE region (human genome reference builds GRCh38 - chromosome: 19, base pair position: 44,407,913 – 45,408,821) were removed due to its pleiotropic nature and large effect size. We then calculated the effects for each individual variant by employing a two-term Taylor series expansion of the Wald ratio. Subsequently, we utilized the weighted delta inverse-variance weighted (IVW) method to conduct a meta-analysis of individual SNP effects, to estimate the combined effect of the Wald ratios. Sensitivity analyses included using various MR methods, including MR-Egger, Weighted Median, Maximum Likelihood, Weighted Mode, and leave-one-out methods.[5]

The same method was then applied to the backward MR (dementia → circulating protein concentration), with SNPs extracted from the same GWAS as described above, except with dementia being the exposure data and protein being the outcome.

All analyses were conducted using human genome reference build GRCh38. In instances where the genome build was based on GRCh37 assembly Hg19, a lift-over process was executed to convert genome coordinates and annotations to the GRCh38 using a designated alignment, using CrossMap 0.7.0 in Python (version 3.12). All MR analyses were conducted using ‘TwoSampleMR’, ‘MendelianRandomization’ and ‘LDlinkR’ R packages.[6]

The selection of the primary cis-MR models included SNPs successfully harmonized with the gene encoding regions, with the flanking region being within 10-kilobase (kB) in either direction of the start and stop coordinates for the genes, according to Human Genome reference release GRCh38. LD clumping was then done to remove the excess of most highly correlated variants at each locus, with R^2^ < 0.001, retaining the SNPs with the strongest associations with the protein of interest filtered by P < 5 × 10^−5^. The sensitivity analyses which took a more liberal approach, selecting valid instruments at the threshold of P < 5 × 10^−5^ and clumped at R^2^ < 0.01.

**Supplementary References**

1. Almeida-Meza, P., A. Steptoe, and D. Cadar, *Markers of cognitive reserve and dementia incidence in the English Longitudinal Study of Ageing.* The British Journal of Psychiatry, 2021. **218**(5): p. 243-251.

2. Kunkle, B.W., et al., *Genetic meta-analysis of diagnosed Alzheimer’s disease identifies new risk loci and implicates Aβ, tau, immunity and lipid processing.* Nature genetics, 2019. **51**(3): p. 414-430.

3. Bellenguez, C., et al., *New insights into the genetic etiology of Alzheimer’s disease and related dementias.* Nature Genetics, 2022. **54**(4): p. 412-436.

4. Kurki, M.I., et al., *FinnGen provides genetic insights from a well-phenotyped isolated population.* Nature, 2023. **613**(7944): p. 508-518.

5. Burgess, S., et al., *Guidelines for performing Mendelian randomization investigations: update for summer 2023.* Wellcome open research, 2019. **4**.

6. Yavorska, O.O. and S. Burgess, *MendelianRandomization: an R package for performing Mendelian randomization analyses using summarized data.* International journal of epidemiology, 2017. **46**(6): p. 1734-1739.

**Supplementary Tables**

**Supplementary Table 1A. International classification of disease codes for definite dementia as recorded in hospital admission and mortality records in ELSA.**

| **ICD Code** | **ICD Description** | **Dementia Category** |
| --- | --- | --- |
| F00 | Dementia in Alzheimer's disease | Definite |
| G30 | Alzheimer's disease | Definite |
| F00.0 | Dementia in Alzheimer's disease with early onset | Definite |
| G30.0 |  |  |
| F00.1 | Dementia in Alzheimer disease with late onset | Definite |
| G30.1 |  |  |
| F00.2 | Dementia in Alzheimer disease, atypical or mixed type | Definite |
| G30.8 | Other Alzheimer's disease | Definite |
| F00.9 | Dementia in Alzheimer's disease, unspecified | Definite |
| G30.9 |  |  |
| F01 | Vascular dementia | Definite |
| F01.0 | Vascular dementia of acute onset | Definite |
| F01.1 | Multi-infarct dementia | Definite |
| F01.2 | Subcortical vascular dementia | Definite |
| F01.3 | Mixed cortical and subcortical vascular dementia | Definite |
| F01.8 | Other vascular dementia | Definite |
| F01.9 | Vascular dementia, unspecified | Definite |
| F02 | Dementia in other diseases classified elsewhere | Definite |
| F02.0 | Dementia in Pick’s disease | Definite |
| F02.1 | Dementia in Creutzfeldt-Jakob disease | Definite |
| F02.2 | Dementia in Huntington's disease | Definite |
| F02.3 | Dementia in Parkinson's disease | Definite |
| F02.4 | Dementia in human immunodeficiency virus [HIV] disease | Definite |
| F02.8 | Dementia in other specified diseases classified elsewhere | Definite |
| G31.0 | Frontotemporal dementia | Definite |
| G31.8 | Other specified degenerative diseases of nervous system  Grey-matter degeneration [Alpers]  Lewy body(ies)(dementia)(disease)  Subacute necrotizing encephalopathy [Leigh] | Definite |
| F03 | Unspecified dementia | Definite |
| F05.1 | Delirium superimposed on dementia | Definite |
| I67.3 | Binswanger's disease. Also known as subcortical leukoencephalopathy, is a form of small vessel vascular dementia | Definite |
| F10.7 | Residual and late-onset psychotic disorder: Includes Alcoholic dementia NOS  Chronic alcoholic brain syndrome  Dementia and other milder forms of persisting impairment of cognitive functions | Definite |
| F04 | Amnestic disorder due to known physiological condition - Korsakov's psychosis or syndrome, non-alcoholic | Definite (listed as dementia by Alzheimer's Association) |
| A81.0 | Creutzfeldt-Jakob disease | Definite (listed as dementia by Alzheimer's Association) |

*References:*

1. *Hayat S, Luben R, Khaw K, et al. Evaluation of routinely collected records for dementia outcomes in UK: a prospective cohort study BMJ Open 2022;12:e060931. doi: 10.1136/bmjopen-2022-060931.*
2. *Wilkinson T, Ly A, Schnier C, Rannikmäe K, Bush K, Brayne C, Quinn TJ, Sudlow CLM. Identifying dementia cases with routinely-collected health data: a systematic review. Alzheimer's & Dementia: The Journal of the Alzheimer's Association.*

**Supplementary Table 1B. International classification of disease codes by dementia sub-type for definite dementia cases in ELSA.**

| **Alzheimer’s Disease** | **Vascular dementia** |
| --- | --- |
| F0.0 | F01 |
| F00.0 | F01.0 |
| F00.1 | F01.1 |
| F00.2 | F01.2 |
| F00.9 | F01.3 |
| G30.0 | F01.8 |
| G30 | F01.9 |
| G30.1 |  |
| G30.8 |  |
| G30.9 |  |

**Supplementary Table 2. Demographic and phenotypic information of the ELSA and UK Biobank participants.**

|  | **Overall** | **ACD** | **AD** | **VAD** | **FTD** |  |
| --- | --- | --- | --- | --- | --- | --- |
| **ELSA, study wave 4 (2008 – 2009)** |  |  |  |  |  |  |
| **Sample N** | 3249 | 229 | 89 | 41 | - |  |
| **Age (years)**, Mean (SD) | 63.4 (9.20) | 75.1 (9.40) | 73.9 (9.25) | 76.5 (9.59) | - |  |
| **Sex**, N (%) |  |  |  |  | - |  |
| Female | 1786 (54.97) | 137 (59.83) | 57 (64.04) | 20 (48.78) | - |  |
| Male | 1463 (45.03) | 92 (40.17) | 32 (35.96) | 21 (51.22) | - |  |
| **Ethnicity**, N (%) |  |  |  |  | - |  |
| White | 3157 (97.17) | 226 (98.69) | 89 (100.00) | 41 (100.00) | - |  |
| Other ethnic groups | 92 (2.83) | 3 (1.31) | 0 (0.00) | 0 (0.00) | - |  |
| **Age completed full-time education**, N (%) |  |  |  |  |  |  |
| None | 16 (0.49) | 3 (1.31) | 2 (2.25) | 0 (0.00) | - |  |
| Age 14 or under | 271 (8.34) | 62 (27.07) | 21 (23.60) | 14 (34.15) | - |  |
| Age 15 | 1023 (31.49) | 72 (31.44) | 28 (31.46) | 11 (26.83) | - |  |
| Age 16 | 745 (22.93) | 33 (14.41) | 9 (10.11) | 7 (17.07) | - |  |
| Age 17 | 265 (8.16) | 15 (6.55) | 7 (7.87) | 1 (2.44) | - |  |
| Age 18 | 248 (7.63) | 12 (5.24) | 5 (5.62) | 1 (2.44) | - |  |
| Age 19 or over | 631 (19.42) | 27 (11.79) | 16 (17.98) | 7 (17.07) | - |  |
| **Smoking status**, N (%) |  |  |  |  |  |  |
| Never smoker | 1319 (40.60) | 96 (41.92) | 35 (39.33) | 14 (34.15) | - |  |
| Former smoker | 1475 (45.40) | 109 (47.60) | 45 (50.56) | 23 (56.10) | - |  |
| Current smoker | 448 (13.79) | 24 (10.48) | 9 (10.11) | 4 (9.76) | - |  |
| **Cardiovascular disease**, N (%) | 259 (7.97) | 42 (18.34) | 11 (12.36) | 9 (21.95) | - |  |
| **Depression**, N (%) | 198 (6.09) | 14 (6.11) | 6 (6.74) | 3 (7.32) |  |  |
| **Body mass index**, Mean (SD) | 28.11 (5.07) | 27.66 (5.05) | 26.65 (4.83) | 28.64 (5.14) | - |  |
| **Systolic blood pressure**, Mean (SD) | 131.7 (17.3) | 136.4 (21.6) | 133.5 (16.6) | 146.2 (22.3) | - |  |
| **LDL cholesterol**, Mean (SD) | 3.28 (1.02) | 2.96 (1.07) | 3.18 (1.08) | 2.70 (1.12) | - |  |
| **UK Biobank, study baseline (2006 – 2010)** |  |  |  |  |  |  |
| **Sample N** | 52745 | 1506 | 732 | 281 | 111 |  |
| **Age (years)**, Mean (SD) | 56.8 (8.21) | 64.5 (5.10) | 65.0 (4.97) | 65.0 (4.53) | 62.1 (6.04) |  |
| **Sex**, N (%) |  |  |  |  |  |  |
| Female | 28436 (53.9) | 732 (48.61) | 402 (54.92) | 110 (39.15) | 48 (43.24) |  |
| Male | 24309 (46.1) | 774 (51.39) | 330 (45.08) | 171 (60.85) | 63 (56.76) |  |
| **Ethnicity**, N (%) |  |  |  |  |  |  |
| White | 49194 (93.3) | 1448 (96.15) | 703 (96.04) | 274 (97.51) | 109 (98.20) |  |
| Black | | 1210 (2.29) | 26 (1.73) | 15 (2.05) | 2 (0.71) | 1 (0.90) |
| Asian | 1132 (2.15) | 9 (0.60) | 3 (0.41) | 2 (0.71) | 0 (0.00) |  |
| Other or mixed | 958 (1.82) | 15 (1.00) | 7 (0.96) | 2 (0.71) | 1 (0.90) |  |
| **Educational attainment**, N (%) |  |  |  |  |  |  |
| College or University degree | 16887 (32.02) | 336 (22.31) | 154 (21.04) | 46 (16.37) | 31 (27.93) |  |
| A levels/AS levels | 5796 (10.99) | 125 (8.30) | 60 (8.20) | 32 (11.39) | 8 (7.21) |  |
| O levels/GCSEs or equivalent | 10894 (20.65) | 291 (19.32) | 148 (20.22) | 58 (20.64) | 14 (12.61) |  |
| CSEs or equivalent | 2813 (5.33) | 25 (1.66) | 9 (1.23) | 1 (0.36) | 4 (3.60) |  |
| NVQ or HND or HNC or equivalent | 3485 (6.61) | 96 (6.37) | 42 (5.74) | 27 (9.61) | 7 (6.31) |  |
| Other professional qualifications e.g.: nursing, teaching | 2780 (5.27) | 88 (5.84) | 42 (5.74) | 16 (5.69) | 10 (9.01) |  |
| None of the above | 9295 (17.62) | 493 (32.74) | 249 (34.02) | 93 (33.10) | 34 (30.63) |  |
| **Smoking status**, N (%) |  |  |  |  |  |  |
| Never smoker | 28533 (54.10) | 722 (47.94) | 363 (49.59) | 116 (41.28) | 54 (48.65) |  |
| Former smoker | 18390 (34.87) | 636 (42.23) | 302 (41.26) | 133 (47.33) | 43 (38.74) |  |
| Current smoker | 5567 (10.55) | 139 (9.23) | 61 (8.33) | 30 (10.68) | 13 (11.71) |  |
| **Cardiovascular disease**, N (%) | 2796 (5.30) | 201 (13.35) | 76 (10.38) | 53 (18.86) | 13 (11.71) |  |
| **Depression,** N (%) | 5835 (11.06) | 177 (11.75) | 70 (9.56) | 39 (13.88) | 23 (20.72) |  |
| **Body mass index**, Mean (SD) | 27.47 (4.81) | 27.77 (4.92) | 27.40 (4.84) | 28.55 (4.99) | 27.58 (4.95) |  |
| **Systolic blood pressure**, Mean (SD) | 139.7 (19.7) | 146.0 (20.5) | 146.8 (20.6) | 146.1 (20.3) | 142.2 (19.5) |  |
| **LDL cholesterol**, Mean (SD) | 3.53 (0.88) | 3.38 (0.93) | 3.42 (0.94) | 3.31 (1.01) | 3.58 (0.94) |  |

*ELSA, English Longitudinal Study of Ageing; SD, standard deviation; ACD, all-cause dementia; AD, Alzheimer’s disease; VAD, vascular dementia; FTD, frontotemporal dementia; LDL, low-density lipoprotein; GCSE, General Certificate of Secondary Education; CSE, Certificate of Secondary Education; NVQ, National Vocational Qualification; HND, Higher National Diploma; HNC, Higher National Certificate.*

**Supplementary Table 3. Multiple-adjusted hazard ratios and 95% confidence intervals for the associations between identified proteins and dementia outcomes in the UK Biobank.**

|  | **HR (95% CI)** | **P_FDR_** | **P_Uncorrected_** |
| --- | --- | --- | --- |
| All-cause dementia | | |  |
| NEFL | 1.87 (1.75, 1.99) | 1.02 × 10^-81^ | 2.54 × 10^-82^ |
| KIM1 | 1.13 (1.06, 1.20) | 3.15 × 10^-4^ | 7.87 × 10^-5^ |
| MMP12 | 1.17 (1.10, 1.24) | 2.00 × 10^-6^ | 4.99 × 10^-7^ |
| EDA2R | 1.31 (1.22, 1.40) | 3.18 × 10^-13^ | 7.94 × 10^-14^ |
| Alzheimer’s disease | | |  |
| NEFL | 1.81 (1.65, 1.99) | 1.89 × 10^-35^ | 4.72 × 10^-36^ |
| KIM1 | 1.11 (1.02, 1.21) | 0.077 | 0.019 |
| MMP12 | 1.10 (1.01, 1.20) | 0.129 | 0.032 |
| EDA2R | 1.25 (1.13, 1.39) | 6.06 × 10^-5^ | 1.52 × 10^-5^ |
| Vascular dementia |  |  |  |
| NEFL | 1.90 (1.64, 2.19) | 1.59 × 10^-17^ | 3.99 × 10^-18^ |
| KIM1 | 1.44 (1.25, 1.66) | 1.13 × 10^-6^ | 2.82 × 10^-7^ |
| MMP12 | 1.36 (1.18, 1.56) | 6.85 × 10^-5^ | 1.71 × 10^-5^ |
| EDA2R | 1.34 (1.15, 1.58) | 1.20 × 10^-3^ | 3.01 × 10^-4^ |
| Frontotemporal dementia | | |  |
| NEFL | 2.97 (2.39, 3.70) | 1.10 × 10^-21^ | 2.75 × 10^-22^ |
| KIM1 | 0.90 (0.72, 1.12) | 0.999 | 0.347 |
| MMP12 | 0.87 (0.70, 1.08) | 0.860 | 0.215 |
| EDA2R | 0.99 (0.77, 1.28) | 0.999 | 0.938 |

*All models adjusted for age, sex, education, ethnicity, smoking status, depression, cardiovascular disease, body mass index, systolic blood pressure, LDL cholesterol. P values were false discovery rate (FDR) corrected.*

**Supplementary Table 4. Two sample bi-directional Mendelian randomization between proteins and dementia.**

| **Gene name** | **Outcome trait** | **MR methods** | **Forward (protein →** **dementia)** | | | **Backward (dementia → protein)** | | |
| --- | --- | --- | --- | --- | --- | --- | --- | --- |
|  |  |  | **No of SNP** | **Slope (SE)** | **P value** | **No of SNP** | **Slope (SE)** | **P value** |
| NEFL | Alzheimer’s disease, *Kunkle et al 2019* | Inverse variant weighting | 7 | -0.208 (0.157) | 0.185 | 19 | 0.019 (0.013) | 0.134 |
| NEFL | Alzheimer’s disease, *Kunkle et al 2019* | MR-Egger | 7 | 0.307 (0.561) | 0.608 | 19 | 0.009 (0.018) | 0.628 |
| NEFL | Alzheimer’s disease, *Kunkle et al 2019* | Weighted Median | 7 | -0.151 (0.213) | 0.479 | 19 | -0.005 (0.013) | 0.724 |
| NEFL | Alzheimer’s disease, *Kunkle et al 2019* | Maximum-likelihood | 7 | -0.211 (0.159) | 0.709 | 19 | 0.019 (0.009) | 0.032 |
| NEFL | Alzheimer’s disease, *Kunkle et al 2019* | Weighted Mode | 7 | 1.028 (2.627) | 0.182 | 19 | 0.001 (0.015) | 0.930 |
| NEFL | Alzheimer’s disease, *Kunkle et al 2019* | Leave-one-out | 7 | -0.208 (0.157) | 0.185 | 19 | 0.019 (0.013) | 0.134 |
| NEFL | Alzheimer’s disease, *Bellenguez et al 2022* | Inverse variant weighting | 9 | 0.017 (0.125) | 0.890 | 59 | 0.056 (0.014) | 1.081×10^-4^ |
| NEFL | Alzheimer’s disease, *Bellenguez et al 2022* | MR-Egger | 9 | 0.592 (0.417) | 0.200 | 59 | 0.030 (0.025) | 0.238 |
| NEFL | Alzheimer’s disease, *Bellenguez et al 2022* | Weighted Median | 9 | 0.080 (0.128) | 0.532 | 59 | 0.029 (0.018) | 0.100 |
| NEFL | Alzheimer’s disease, *Bellenguez et al 2022* | Maximum-likelihood | 9 | 0.020 (0.077) | 0.803 | 59 | 0.057 (0.011) | 4.831×10^-7^ |
| NEFL | Alzheimer’s disease, *Bellenguez et al 2022* | Weighted Mode | 9 | -1.156 (1.990) | 0.577 | 59 | 0.024 (0.020) | 0.233 |
| NEFL | Alzheimer’s disease, *Bellenguez et al 2022* | Leave-one-out | 9 | 0.017 (0.125) | 0.890 | 59 | 0.056 (0.014) | 1.081×10^-4^ |
| NEFL | Alzheimer’s disease, *FinnGen 2023* | Inverse variant weighting | 9 | 0.086 (0.158) | 0.584 | 22 | 0.033 (0.015) | 0.024 |
| NEFL | Alzheimer’s disease, *FinnGen 2023* | MR-Egger | 9 | -0.464 (0.569) | 0.442 | 22 | 0.010 (0.022) | 0.655 |
| NEFL | Alzheimer’s disease, *FinnGen 2023* | Weighted Median | 9 | 0.156 (0.167) | 0.352 | 22 | 0.022 (0.013) | 0.099 |
| NEFL | Alzheimer’s disease, *FinnGen 2023* | Maximum-likelihood | 9 | 0.091 (0.120) | 0.448 | 22 | 0.034 (0.010) | 0.001 |
| NEFL | Alzheimer’s disease, *FinnGen 2023* | Weighted Mode | 9 | -1.921 (3.008) | 0.541 | 22 | 0.019 (0.014) | 0.183 |
| NEFL | Alzheimer’s disease, *FinnGen 2023* | Leave-one-out | 9 | 0.086 (0.158) | 0.584 | 22 | 0.033 (0.015) | 0.024 |
| NEFL | All-cause dementia, *FinnGen 2023* | Inverse variant weighting | 9 | 0.024 (0.175) | 0.889 | 22 | 0.030 (0.017) | 0.080 |
| NEFL | All-cause dementia, *FinnGen 2023* | MR-Egger | 9 | -0.608 (0.630) | 0.366 | 22 | 0.019 (0.026) | 0.470 |
| NEFL | All-cause dementia, *FinnGen 2023* | Weighted Median | 9 | 0.235 (0.158) | 0.136 | 22 | 0.019 (0.015) | 0.189 |
| NEFL | All-cause dementia, *FinnGen 2023* | Maximum-likelihood | 9 | 0.027 (0.111) | 0.807 | 22 | 0.031 (0.012) | 0.010 |
| NEFL | All-cause dementia, *FinnGen 2023* | Weighted Mode | 9 | -1.852 (2.811) | 0.528 | - | - | - |
| NEFL | All-cause dementia, *FinnGen 2023* | Leave-one-out | 9 | 0.024 (0.175) | 0.889 | 22 | 0.030 (0.017) | 0.080 |
| NEFL | Vascular dementia, *FinnGen 2023* | Inverse variant weighting | 9 | 0.050 (0.269) | 0.854 | 4 | 0.035 (0.019) | 0.058 |
| NEFL | Vascular dementia, *FinnGen 2023* | MR-Egger | 9 | -1.541 (0.927) | 0.140 | 4 | 0.057 (0.018) | 0.090 |
| NEFL | Vascular dementia, *FinnGen 2023* | Weighted Median | 9 | -0.052 (0.343) | 0.880 | 4 | 0.035 (0.014) | 0.009 |
| NEFL | Vascular dementia, *FinnGen 2023* | Maximum-likelihood | 9 | 0.052 (0.263) | 0.844 | 4 | 0.036 (0.014) | 0.008 |
| NEFL | Vascular dementia, *FinnGen 2023* | Weighted Mode | 9 | -2.777 (5.385) | 0.620 | - | - | - |
| NEFL | Vascular dementia, *FinnGen 2023* | Leave-one-out | 9 | -0.050 (0.027) | 0.853 | 4 | 0.035 (0.019) | 0.058 |
| KIM1 | Alzheimer’s disease, *Kunkle et al 2019* | Inverse variant weighting | 22 | -0.024 (0.032) | 0.451 | 19 | -0.007 (0.013) | 0.594 |
| KIM1 | Alzheimer’s disease, *Kunkle et al 2019* | MR-Egger | 22 | -0.064 (0.045) | 0.174 | 19 | -0.017 (0.018) | 0.362 |
| KIM1 (HAVCR1) | Alzheimer’s disease, *Kunkle et al 2019* | Weighted Median | 22 | -0.042 (0.039) | 0.277 | 19 | -0.011 (0.012) | 0.367 |
| KIM1 (HAVCR1) | Alzheimer’s disease, *Kunkle et al 2019* | Maximum-likelihood | 22 | -0.024 (0.032) | 0.452 | 19 | -0.007 (0.008) | 0.424 |
| KIM1 (HAVCR1) | Alzheimer’s disease, *Kunkle et al 2019* | Weighted Mode | 22 | -0.039 (0.039) | 0.332 | 19 | -0.011 (0.012) | 0.377 |
| KIM1 (HAVCR1) | Alzheimer’s disease, *Kunkle et al 2019* | Leave-one-out | 22 | -0.024 (0.032) | 0.451 | 19 | -0.007 (0.013) | 0.594 |
| KIM1 (HAVCR1) | Alzheimer’s disease, *Bellenguez et al 2022* | Inverse variant weighting | 27 | -0.022 (0.026) | 0.393 | 59 | -0.011 (0.014) | 0.444 |
| KIM1 (HAVCR1) | Alzheimer’s disease, *Bellenguez et al 2022* | MR-Egger | 27 | -0.034 (0.038) | 0.372 | 59 | -0.005 (0.024) | 0.825 |
| KIM1 (HAVCR1) | Alzheimer’s disease, *Bellenguez et al 2022* | Weighted Median | 27 | -0.018 (0.023) | 0.432 | 59 | -0.004 (0.017) | 0.831 |
| KIM1 (HAVCR1) | Alzheimer’s disease, *Bellenguez et al 2022* | Maximum-likelihood | 27 | -0.023 (0.018) | 0.196 | 59 | -0.011 (0.010) | 0.296 |
| KIM1 (HAVCR1) | Alzheimer’s disease, *Bellenguez et al 2022* | Weighted Mode | 27 | -0.019 (0.022) | 0.391 | 59 | -0.013 (0.021) | 0.557 |
| KIM1 (HAVCR1) | Alzheimer’s disease, *Bellenguez et al 2022* | Leave-one-out | 27 | -0.022 (0.026) | 0.393 | 59 | -0.011 (0.014) | 0.444 |
| KIM1 (HAVCR1) | Alzheimer’s disease, *FinnGen 2023* | Inverse variant weighting | 26 | 0.002 (0.040) | 0.966 | 22 | 0.003 (0.014) | 0.839 |
| KIM1 (HAVCR1) | Alzheimer’s disease, *FinnGen 2023* | MR-Egger | 26 | 0.002 (0.058) | 0.971 | 22 | -0.006 (0.021) | 0.789 |
| KIM1 (HAVCR1) | Alzheimer’s disease, *FinnGen 2023* | Weighted Median | 26 | -0.034 (0.043) | 0.430 | 22 | -0.014 (0.012) | 0.265 |
| KIM1 (HAVCR1) | Alzheimer’s disease, *FinnGen 2023* | Maximum-likelihood | 26 | 0.002 (0.028) | 0.951 | 22 | 0.003 (0.009) | 0.767 |
| KIM1 (HAVCR1) | Alzheimer’s disease, *FinnGen 2023* | Weighted Mode | 26 | -0.013 (0.037) | 0.716 | 22 | -0.012 (0.012) | 0.334 |
| KIM1 (HAVCR1) | Alzheimer’s disease, *FinnGen 2023* | Leave-one-out | 26 | 0.002 (0.040) | 0.966 | 22 | 0.003 (0.014) | 0.839 |
| KIM1 (HAVCR1) | All-cause dementia, *FinnGen 2023* | Inverse variant weighting | 26 | -0.001 (0.042) | 0.999 | 22 | 0.007 (0.017) | 0.659 |
| KIM1 (HAVCR1) | All-cause dementia, *FinnGen 2023* | MR-Egger | 26 | 0.014 (0.062) | 0.823 | 22 | -0.013 (0.025) | 0.613 |
| KIM1 (HAVCR1) | All-cause dementia, *FinnGen 2023* | Weighted Median | 26 | -0.068 (0.038) | 0.073 | 22 | -0.015 (0.014) | 0.271 |
| KIM1 (HAVCR1) | All-cause dementia, *FinnGen 2023* | Maximum-likelihood | 26 | -0.001 (0.025) | 0.998 | 22 | 0.008 (0.011) | 0.484 |
| KIM1 (HAVCR1) | All-cause dementia, *FinnGen 2023* | Weighted Mode | 26 | -0.053 (0.036) | 0.153 | 22 | -0.382 (1.619) | 0.816 |
| KIM1 (HAVCR1) | All-cause dementia, *FinnGen 2023* | Leave-one-out | 26 | -0.001 (0.042) | 0.999 | 22 | 0.007 (0.017) | 0.659 |
| KIM1 (HAVCR1) | Vascular dementia, *FinnGen 2023* | Inverse variant weighting | 26 | -0.067 (0.067) | 0.315 | 4 | -0.007 (0.012) | 0.590 |
| KIM1 (HAVCR1) | Vascular dementia, *FinnGen 2023* | MR-Egger | 26 | -0.010 (0.097) | 0.918 | 4 | 0.009 (0.017) | 0.658 |
| KIM1 (HAVCR1) | Vascular dementia, *FinnGen 2023* | Weighted Median | 26 | -0.142 (0.085) | 0.093 | 4 | -0.007 (0.012) | 0.594 |
| KIM1 (HAVCR1) | Vascular dementia, *FinnGen 2023* | Maximum-likelihood | 26 | -0.068 (0.061) | 0.270 | 4 | -0.007 (0.012) | 0.589 |
| KIM1 (HAVCR1) | Vascular dementia, *FinnGen 2023* | Weighted Mode | 26 | -0.074 (0.077) | 0.345 | - | - | - |
| KIM1 (HAVCR1) | Vascular dementia, *FinnGen 2023* | Leave-one-out | 26 | -0.067 (0.067) | 0.315 | 4 | -0.007 (0.012) | 0.590 |
| MMP12 | Alzheimer’s disease, *Kunkle et al 2019* | Inverse variant weighting | 10 | -0.013 (0.039) | 0.745 | 19 | 0.016 (0.009) | 0.072 |
| MMP12 | Alzheimer’s disease, *Kunkle et al 2019* | MR-Egger | 10 | 0.022 (0.045) | 0.649 | 19 | 0.027 (0.012) | 0.043 |
| MMP12 | Alzheimer’s disease, *Kunkle et al 2019* | Weighted Median | 10 | -0.002 (0.032) | 0.954 | 19 | 0.025 (0.012) | 0.037 |
| MMP12 | Alzheimer’s disease, *Kunkle et al 2019* | Maximum-likelihood | 10 | -0.013 (0.030) | 0.673 | 19 | 0.016 (0.009) | 0.072 |
| MMP12 | Alzheimer’s disease, *Kunkle et al 2019* | Weighted Mode | 10 | -0.002 (0.030) | 0.944 | 19 | 0.026 (0.012) | 0.037 |
| MMP12 | Alzheimer’s disease, *Kunkle et al 2019* | Leave-one-out | 10 | -0.013 (0.039) | 0.745 | 19 | 0.016 (0.009) | 0.072 |
| MMP12 | Alzheimer’s disease, *Bellenguez et al 2022* | Inverse variant weighting | 8 | -0.017 (0.046) | 0.708 | 59 | 0.002 (0.016) | 0.916 |
| MMP12 | Alzheimer’s disease, *Bellenguez et al 2022* | MR-Egger | 8 | 0.003 (0.058) | 0.965 | 59 | 0.024 (0.028) | 0.396 |
| MMP12 | Alzheimer’s disease, *Bellenguez et al 2022* | Weighted Median | 8 | -0.012 (0.018) | 0.491 | 59 | 0.017 (0.018) | 0.333 |
| MMP12 | Alzheimer’s disease, *Bellenguez et al 2022* | Maximum-likelihood | 8 | -0.017 (0.017) | 0.312 | 59 | 0.002 (0.011) | 0.880 |
| MMP12 | Alzheimer’s disease, *Bellenguez et al 2022* | Weighted Mode | 8 | -0.009 (0.018) | 0.629 | 59 | 0.017 (0.019) | 0.386 |
| MMP12 | Alzheimer’s disease, *Bellenguez et al 2022* | Leave-one-out | 8 | -0.017 (0.046) | 0.708 | 59 | 0.002 (0.016) | 0.916 |
| MMP12 | Alzheimer’s disease, *FinnGen 2023* | Inverse variant weighting | 10 | -0.006 (0.033) | 0.862 | 22 | 0.016 (0.011) | 0.134 |
| MMP12 | Alzheimer’s disease, *FinnGen 2023* | MR-Egger | 10 | 0.014 (0.042) | 0.739 | 22 | 0.021 (0.017) | 0.230 |
| MMP12 | Alzheimer’s disease, *FinnGen 2023* | Weighted Median | 10 | -0.002 (0.023) | 0.936 | 22 | 0.023 (0.013) | 0.082 |
| MMP12 | Alzheimer’s disease, *FinnGen 2023* | Maximum-likelihood | 10 | -0.006 (0.022) | 0.789 | 22 | 0.016 (0.010) | 0.104 |
| MMP12 | Alzheimer’s disease, *FinnGen 2023* | Weighted Mode | 10 | -0.004 (0.023) | 0.858 | 22 | 0.020 (0.014) | 0.177 |
| MMP12 | Alzheimer’s disease, *FinnGen 2023* | Leave-one-out | 10 | -0.006 (0.033) | 0.862 | 22 | 0.016 (0.011) | 0.134 |
| MMP12 | All-cause dementia, *FinnGen 2023* | Inverse variant weighting | 10 | -0.004 (0.027) | 0.875 | 22 | 0.007 (0.015) | 0.625 |
| MMP12 | All-cause dementia, *FinnGen 2023* | MR-Egger | 10 | -0.001 (0.035) | 0.975 | 22 | 0.029 (0.022) | 0.195 |
| MMP12 | All-cause dementia, *FinnGen 2023* | Weighted Median | 10 | -0.006 (0.021) | 0.794 | 22 | 0.018 (0.015) | 0.235 |
| MMP12 | All-cause dementia, *FinnGen 2023* | Maximum-likelihood | 10 | -0.004 (0.020) | 0.833 | 22 | 0.007 (0.012) | 0.521 |
| MMP12 | All-cause dementia, *FinnGen 2023* | Weighted Mode | 10 | -0.006 (0.021) | 0.799 | 22 | - | - |
| MMP12 | All-cause dementia, *FinnGen 2023* | Leave-one-out | 10 | -0.004 (0.027) | 0.875 | 22 | 0.007 (0.015) | 0.625 |
| MMP12 | Vascular dementia, *FinnGen 2023* | Inverse variant weighting | 10 | -0.033 (0.048) | 0.490 | 4 | 0.006 (0.025) | 0.808 |
| MMP12 | Vascular dementia, *FinnGen 2023* | MR-Egger | 10 | -0.006 (0.059) | 0.926 | 4 | 0.035 (0.026) | 0.313 |
| MMP12 | Vascular dementia, *FinnGen 2023* | Weighted Median | 10 | -0.025 (0.049) | 0.607 | 4 | 0.007 (0.013) | 0.584 |
| MMP12 | Vascular dementia, *FinnGen 2023* | Maximum-likelihood | 10 | -0.034 (0.048) | 0.488 | 4 | 0.006 (0.013) | 0.629 |
| MMP12 | Vascular dementia, *FinnGen 2023* | Weighted Mode | 10 | -0.028 (0.050) | 0.586 | 4 | - | - |
| MMP12 | Vascular dementia, *FinnGen 2023* | Leave-one-out | 10 | -0.033 (0.048) | 0.490 | 4 | 0.006 (0.025) | 0.808 |
| EDA2R | Alzheimer’s disease, *Kunkle et al 2019* | Inverse variant weighting | 16 | 0.106 (0.113) | 0.348 | 19 | -0.014 (0.008) | 0.085 |
| EDA2R | Alzheimer’s disease, *Kunkle et al 2019* | MR-Egger | 16 | 0.127 (0.312) | 0.689 | 19 | -0.013 (0.012) | 0.269 |
| EDA2R | Alzheimer’s disease, *Kunkle et al 2019* | Weighted Median | 16 | 0.163 (0.141) | 0.250 | 19 | -0.017 (0.012) | 0.143 |
| EDA2R | Alzheimer’s disease, *Kunkle et al 2019* | Maximum-likelihood | 16 | 0.112 (0.102) | 0.399 | 19 | -0.014 (0.008) | 0.075 |
| EDA2R | Alzheimer’s disease, *Kunkle et al 2019* | Weighted Mode | 16 | 0.196 (0.226) | 0.270 | 19 | -0.018 (0.011) | 0.134 |
| EDA2R | Alzheimer’s disease, *Kunkle et al 2019* | Leave-one-out | 16 | 0.106 (0.113) | 0.348 | 19 | -0.014 (0.008) | 0.085 |
| EDA2R | Alzheimer’s disease, *Bellenguez et al 2022* | Inverse variant weighting | 17 | 0.051 (0.070) | 0.471 | 59 | -0.015 (0.012) | 0.205 |
| EDA2R | Alzheimer’s disease, *Bellenguez et al 2022* | MR-Egger | 17 | 0.166 (0.186) | 0.386 | 59 | -0.012 (0.021) | 0.568 |
| EDA2R | Alzheimer’s disease, *Bellenguez et al 2022* | Weighted Median | 17 | 0.111 (0.084) | 0.185 | 59 | -0.012 (0.017) | 0.467 |
| EDA2R | Alzheimer’s disease, *Bellenguez et al 2022* | Maximum-likelihood | 17 | 0.055 (0.054) | 0.309 | 59 | -0.015 (0.010) | 0.128 |
| EDA2R | Alzheimer’s disease, *Bellenguez et al 2022* | Weighted Mode | 17 | 0.237 (0.169) | 0.180 | 59 | -0.034 (0.024) | 0.155 |
| EDA2R | Alzheimer’s disease, *Bellenguez et al 2022* | Leave-one-out | 17 | 0.051 (0.070) | 0.471 | 59 | -0.015 (0.012) | 0.205 |
| EDA2R | Alzheimer’s disease, *FinnGen 2023* | Inverse variant weighting | 16 | 0.259 (0.096) | 0.007 | 22 | 0.013 (0.049) | 0.049 |
| EDA2R | Alzheimer’s disease, *FinnGen 2023* | MR-Egger | 16 | 0.221 (0.244) | 0.382 | 22 | 0.020 (0.432) | 0.432 |
| EDA2R | Alzheimer’s disease, *FinnGen 2023* | Weighted Median | 16 | 0.194 (0.122) | 0.114 | 22 | 0.012 (0.016) | 0.016 |
| EDA2R | Alzheimer’s disease, *FinnGen 2023* | Maximum-likelihood | 16 | 0.267 (0.085) | 0.002 | 22 | 0.009 (0.006) | 0.006 |
| EDA2R | Alzheimer’s disease, *FinnGen 2023* | Weighted Mode | 16 | 0.238 (0.169) | 0.181 | 22 | 0.012 (0.039) | 0.039 |
| EDA2R | Alzheimer’s disease, *FinnGen 2023* | Leave-one-out | 16 | 0.259 (0.096) | 0.007 | 22 | 0.013 (0.049) | 0.049 |
| EDA2R | All-cause dementia, *FinnGen 2023* | Inverse variant weighting | 16 | 0.232 (0.110) | 0.035 | 22 | -0.032 (0.012) | 0.007 |
| EDA2R | All-cause dementia, *FinnGen 2023* | MR-Egger | 16 | 0.457 (0.272) | 0.115 | 22 | -0.030 (0.018) | 0.114 |
| EDA2R | All-cause dementia, *FinnGen 2023* | Weighted Median | 16 | 0.246 (0.111) | 0.027 | 22 | -0.036 (0.013) | 0.006 |
| EDA2R | All-cause dementia, *FinnGen 2023* | Maximum-likelihood | 16 | 0.241 (0.078) | 0.002 | 22 | -0.032 (0.011) | 0.003 |
| EDA2R | All-cause dementia, *FinnGen 2023* | Weighted Mode | 16 | 0.329 (0.143) | 0.036 | 22 | -0.570 (1.723) | 0.744 |
| EDA2R | All-cause dementia, *FinnGen 2023* | Leave-one-out | 16 | 0.232 (0.110) | 0.035 | 22 | -0.032 (0.012) | 0.007 |
| EDA2R | Vascular dementia, *FinnGen 2023* | Inverse variant weighting | 16 | 0.127 (0.194) | 0.512 | 4 | -0.037 (0.020) | 0.058 |
| EDA2R | Vascular dementia, *FinnGen 2023* | MR-Egger | 16 | -0.390 (0.474) | 0.424 | 4 | -0.013 (0.017) | 0.513 |
| EDA2R | Vascular dementia, *FinnGen 2023* | Weighted Median | 16 | 0.083 (0.247) | 0.737 | 4 | -0.036 (0.012) | 0.003 |
| EDA2R | Vascular dementia, *FinnGen 2023* | Maximum-likelihood | 16 | 0.131 (0.186) | 0.480 | 4 | -0.038 (0.012) | 0.002 |
| EDA2R | Vascular dementia, *FinnGen 2023* | Weighted Mode | 16 | -0.014 (0.317) | 0.966 | - | - | - |
| EDA2R | Vascular dementia, *FinnGen 2023* | Leave-one-out | 16 | 0.127 (0.194) | 0.512 | 4 | -0.037 (0.020) | 0.058 |

*MR, Mendelian randomization; SNP, single nucleotide polymorphism; SE, standard error.*

**Supplementary Table 5. Two sample cis-Mendelian randomization between proteins and dementia.**

| **Gene name** | **Outcome trait** | **MR methods** | **Cis-MR (protein → dementia)**  **Main analysis** (P < 5 × 10^−5^, R^2^ < 0.001) | | | **Cis-MR (protein → dementia)**  **Sensitivity analysis** (P < 5 × 10^−5^, R^2^ < 0.01) | | |
| --- | --- | --- | --- | --- | --- | --- | --- | --- |
|  |  |  | **No of SNP** | **Slope (SE)** | **P value** | **No of SNP** | **Slope (SE)** | **P value** |
| NEFL | Alzheimer’s disease, *Kunkle et al 2019* | Inverse variant weighting | 2 | 0.820 (0.485) | 0.091 | 2 | 0.820 (0.485) | 0.091 |
| NEFL | Alzheimer’s disease, *Kunkle et al 2019* | MR-Egger | 2 | - | - | 2 | - | - |
| NEFL | Alzheimer’s disease, *Kunkle et al 2019* | Weighted Median | 2 | - | - | 2 | - | - |
| NEFL | Alzheimer’s disease, *Kunkle et al 2019* | Maximum-likelihood | 2 | 0.857 (0.516) | 0.097 | 2 | 0.857 (0.516) | 0.097 |
| NEFL | Alzheimer’s disease, *Kunkle et al 2019* | Weighted Mode | 2 | - | - | 2 | - | - |
| NEFL | Alzheimer’s disease, *Kunkle et al 2019* | Leave-one-out | 2 | 0.820 (0.485) | 0.091 | 2 | 0.820 (0.485) | 0.091 |
| NEFL | Alzheimer’s disease, *Bellenguez et al 2022* | Inverse variant weighting | 2 | 0.303 (0.372) | 0.414 | 2 | 0.303 (0.372) | 0.414 |
| NEFL | Alzheimer’s disease, *Bellenguez et al 2022* | MR-Egger | 2 | - | - | 2 | - | - |
| NEFL | Alzheimer’s disease, *Bellenguez et al 2022* | Weighted Median | 2 | - | - | 2 | - | - |
| NEFL | Alzheimer’s disease, *Bellenguez et al 2022* | Maximum-likelihood | 2 | 0.331 (0.277) | 0.232 | 2 | 0.331 (0.277) | 0.232 |
| NEFL | Alzheimer’s disease, *Bellenguez et al 2022* | Weighted Mode | 2 | - | - | 2 | - | - |
| NEFL | Alzheimer’s disease, *Bellenguez et al 2022* | Leave-one-out | 2 | 0.303 (0.372) | 0.414 | 2 | 0.303 (0.372) | 0.414 |
| NEFL | Alzheimer’s disease, *FinnGen 2023* | Inverse variant weighting | 2 | 0.620 (0.392) | 0.114 | 2 | 0.620 (0.392) | 0.114 |
| NEFL | Alzheimer’s disease, *FinnGen 2023* | MR-Egger | 2 | - | - | 2 | - | - |
| NEFL | Alzheimer’s disease, *FinnGen 2023* | Weighted Median | 2 | - | - | 2 | - | - |
| NEFL | Alzheimer’s disease, *FinnGen 2023* | Maximum-likelihood | 2 | 0.621 (0.414) | 0.133 | 2 | 0.621 (0.414) | 0.133 |
| NEFL | Alzheimer’s disease, *FinnGen 2023* | Weighted Mode | 2 | - | - | 2 | - | - |
| NEFL | Alzheimer’s disease, *FinnGen 2023* | Leave-one-out | 2 | 0.620 (0.392) | 0.114 | 2 | 0.620 (0.392) | 0.114 |
| NEFL | All-cause dementia, *FinnGen 2023* | Inverse variant weighting | 2 | 0.161 (0.358) | 0.652 | 2 | 0.161 (0.358) | 0.652 |
| NEFL | All-cause dementia, *FinnGen 2023* | MR-Egger | 2 | - | - | 2 | - | - |
| NEFL | All-cause dementia, *FinnGen 2023* | Weighted Median | 2 | - | - | 2 | - | - |
| NEFL | All-cause dementia, *FinnGen 2023* | Maximum-likelihood | 2 | 0.162 (0.360) | 0.653 | 2 | 0.162 (0.360) | 0.653 |
| NEFL | All-cause dementia, *FinnGen 2023* | Weighted Mode | 2 | - | - | 2 | - | - |
| NEFL | All-cause dementia, *FinnGen 2023* | Leave-one-out | 2 | 0.161 (0.358) | 0.652 | 2 | 0.161 (0.358) | 0.652 |
| NEFL | Vascular dementia, *FinnGen 2023* | Inverse variant weighting | 2 | -0.222 (1.166) | 0.849 | 2 | -0.222 (1.166) | 0.849 |
| NEFL | Vascular dementia, *FinnGen 2023* | MR-Egger | 2 | - | - | 2 | - | - |
| NEFL | Vascular dementia, *FinnGen 2023* | Weighted Median | 2 | - | - | 2 | - | - |
| NEFL | Vascular dementia, *FinnGen 2023* | Maximum-likelihood | 2 | -0.235 (0.895) | 0.793 | 2 | -0.235 (0.895) | 0.793 |
| NEFL | Vascular dementia, *FinnGen 2023* | Weighted Mode | 2 | - | - | 2 | - | - |
| NEFL | Vascular dementia, *FinnGen 2023* | Leave-one-out | 2 | -0.222 (1.166) | 0.849 | 2 | -0.222 (1.166) | 0.849 |
| KIM1 (HAVCR1) | Alzheimer’s disease, *Kunkle et al 2019* | Inverse variant weighting | 4 | -0.030 (0.042) | 0.473 | 9 | -0.006 (0.039) | 0.882 |
| KIM1 (HAVCR1) | Alzheimer’s disease, *Kunkle et al 2019* | MR-Egger | 4 | -0.274 (0.114) | 0.138 | 9 | 0.021 (0.101) | 0.837 |
| KIM1 (HAVCR1) | Alzheimer’s disease, *Kunkle et al 2019* | Weighted Median | 4 | -0.025 (0.035) | 0.471 | 9 | -0.012 (0.036) | 0.731 |
| KIM1 (HAVCR1) | Alzheimer’s disease, *Kunkle et al 2019* | Maximum-likelihood | 4 | -0.030 (0.032) | 0.354 | 9 | -0.006 (0.032) | 0.858 |
| KIM1 (HAVCR1) | Alzheimer’s disease, *Kunkle et al 2019* | Weighted Mode | 4 | -0.008 (0.040) | 0.853 | 9 | -0.011 (0.036) | 0.767 |
| KIM1 (HAVCR1) | Alzheimer’s disease, *Kunkle et al 2019* | Leave-one-out | 4 | -0.030 (0.042) | 0.473 | 9 | -0.006 (0.039) | 0.882 |
| KIM1 (HAVCR1) | Alzheimer’s disease, *Bellenguez et al 2022* | Inverse variant weighting | 4 | 0.004 (0.017) | 0.814 | 10 | 0.003 (0.015) | 0.858 |
| KIM1 (HAVCR1) | Alzheimer’s disease, *Bellenguez et al 2022* | MR-Egger | 4 | 0.040 (0.055) | 0.542 | 10 | 0.019 (0.030) | 0.556 |
| KIM1 (HAVCR1) | Alzheimer’s disease, *Bellenguez et al 2022* | Weighted Median | 4 | 0.006 (0.020) | 0.761 | 10 | 0.005 (0.019) | 0.801 |
| KIM1 (HAVCR1) | Alzheimer’s disease, *Bellenguez et al 2022* | Maximum-likelihood | 4 | 0.004 (0.017) | 0.814 | 10 | 0.003 (0.015) | 0.859 |
| KIM1 (HAVCR1) | Alzheimer’s disease, *Bellenguez et al 2022* | Weighted Mode | 4 | 0.006 (0.019) | 0.766 | 10 | 0.006 (0.017) | 0.737 |
| KIM1 (HAVCR1) | Alzheimer’s disease, *Bellenguez et al 2022* | Leave-one-out | 4 | 0.004 (0.017) | 0.814 | 10 | 0.003 (0.015) | 0.858 |
| KIM1 (HAVCR1) | Alzheimer’s disease, *FinnGen 2023* | Inverse variant weighting | 4 | -0.028 (0.039) | 0.471 | 10 | -0.038 (0.023) | 0.090 |
| KIM1 (HAVCR1) | Alzheimer’s disease, *FinnGen 2023* | MR-Egger | 4 | -0.135 (0.088) | 0.264 | 10 | -0.102 (0.041) | 0.037 |
| KIM1 (HAVCR1) | Alzheimer’s disease, *FinnGen 2023* | Weighted Median | 4 | -0.047 (0.027) | 0.078 | 10 | -0.046 (0.027) | 0.086 |
| KIM1 (HAVCR1) | Alzheimer’s disease, *FinnGen 2023* | Maximum-likelihood | 4 | -0.028 (0.025) | 0.267 | 10 | -0.038 (0.023) | 0.091 |
| KIM1 (HAVCR1) | Alzheimer’s disease, *FinnGen 2023* | Weighted Mode | 4 | -0.050 (0.026) | 0.154 | 10 | -0.053 (0.026) | 0.072 |
| KIM1 (HAVCR1) | Alzheimer’s disease, *FinnGen 2023* | Leave-one-out | 4 | -0.028 (0.039) | 0.471 | 10 | -0.038 (0.023) | 0.090 |
| KIM1 (HAVCR1) | All-cause dementia, *FinnGen 2023* | Inverse variant weighting | 4 | -0.025 (0.037) | 0.504 | 10 | -0.026 (0.021) | 0.219 |
| KIM1 (HAVCR1) | All-cause dementia, *FinnGen 2023* | MR-Egger | 4 | -0.105 (0.099) | 0.401 | 10 | -0.094 (0.037) | 0.036 |
| KIM1 (HAVCR1) | All-cause dementia, *FinnGen 2023* | Weighted Median | 4 | -0.046 (0.026) | 0.075 | 10 | -0.049 (0.024) | 0.043 |
| KIM1 (HAVCR1) | All-cause dementia, *FinnGen 2023* | Maximum-likelihood | 4 | -0.025 (0.023) | 0.278 | 10 | -0.026 (0.021) | 0.218 |
| KIM1 (HAVCR1) | All-cause dementia, *FinnGen 2023* | Weighted Mode | 4 | -0.044 (0.024) | 0.157 | 10 | -0.048 (0.022) | 0.060 |
| KIM1 (HAVCR1) | All-cause dementia, *FinnGen 2023* | Leave-one-out | 4 | -0.025 (0.037) | 0.504 | 10 | -0.026 (0.021) | 0.219 |
| KIM1 (HAVCR1) | Vascular dementia, *FinnGen 2023* | Inverse variant weighting | 4 | 0.001 (0.057) | 0.986 | 10 | 0.009 (0.058) | 0.878 |
| KIM1 (HAVCR1) | Vascular dementia, *FinnGen 2023* | MR-Egger | 4 | -0.028 (0.172) | 0.885 | 10 | -0.002 (0.112) | 0.983 |
| KIM1 (HAVCR1) | Vascular dementia, *FinnGen 2023* | Weighted Median | 4 | -0.017 (0.061) | 0.785 | 10 | -0.001 (0.062) | 0.993 |
| KIM1 (HAVCR1) | Vascular dementia, *FinnGen 2023* | Maximum-likelihood | 4 | 0.001 (0.057) | 0.986 | 10 | 0.009 (0.051) | 0.863 |
| KIM1 (HAVCR1) | Vascular dementia, *FinnGen 2023* | Weighted Mode | 4 | -0.025 (0.062) | 0.713 | 10 | -0.028 (0.056) | 0.630 |
| KIM1 (HAVCR1) | Vascular dementia, *FinnGen 2023* | Leave-one-out | 4 | 0.001 (0.057) | 0.986 | 10 | 0.009 (0.058) | 0.878 |
| MMP12 | Alzheimer’s disease, *Kunkle et al 2019* | Inverse variant weighting | 4 | -0.008 (0.031) | 0.787 | 9 | -0.003 (0.027) | 0.919 |
| MMP12 | Alzheimer’s disease, *Kunkle et al 2019* | MR-Egger | 4 | 0.010 (0.044) | 0.838 | 9 | -0.011 (0.045) | 0.819 |
| MMP12 | Alzheimer’s disease, *Kunkle et al 2019* | Weighted Median | 4 | -0.003 (0.031) | 0.935 | 9 | 0.000 (0.030) | 0.989 |
| MMP12 | Alzheimer’s disease, *Kunkle et al 2019* | Maximum-likelihood | 4 | -0.008 (0.031) | 0.787 | 9 | -0.003 (0.027) | 0.919 |
| MMP12 | Alzheimer’s disease, *Kunkle et al 2019* | Weighted Mode | 4 | 0.000 (0.032) | 0.995 | 9 | -0.002 (0.028) | 0.950 |
| MMP12 | Alzheimer’s disease, *Kunkle et al 2019* | Leave-one-out | 4 | -0.008 (0.031) | 0.787 | 9 | -0.003 (0.027) | 0.919 |
| MMP12 | Alzheimer’s disease, *Bellenguez et al 2022* | Inverse variant weighting | 3 | -0.011 (0.018) | 0.528 | 9 | -0.009 (0.015) | 0.546 |
| MMP12 | Alzheimer’s disease, *Bellenguez et al 2022* | MR-Egger | 3 | -0.019 (0.025) | 0.580 | 9 | -0.009 (0.025) | 0.746 |
| MMP12 | Alzheimer’s disease, *Bellenguez et al 2022* | Weighted Median | 3 | -0.012 (0.018) | 0.487 | 9 | -0.011 (0.017) | 0.512 |
| MMP12 | Alzheimer’s disease, *Bellenguez et al 2022* | Maximum-likelihood | 3 | -0.011 (0.018) | 0.528 | 9 | -0.009 (0.015) | 0.545 |
| MMP12 | Alzheimer’s disease, *Bellenguez et al 2022* | Weighted Mode | 3 | -0.013 (0.018) | 0.562 | 9 | -0.011 (0.017) | 0.514 |
| MMP12 | Alzheimer’s disease, *Bellenguez et al 2022* | Leave-one-out | 3 | -0.011 (0.018) | 0.528 | 9 | -0.009 (0.015) | 0.546 |
| MMP12 | Alzheimer’s disease, *FinnGen 2023* | Inverse variant weighting | 4 | -0.001 (0.022) | 0.981 | 9 | 0.010 (0.020) | 0.620 |
| MMP12 | Alzheimer’s disease, *FinnGen 2023* | MR-Egger | 4 | -0.015 (0.034) | 0.701 | 9 | -0.003 (0.033) | 0.922 |
| MMP12 | Alzheimer’s disease, *FinnGen 2023* | Weighted Median | 4 | -0.002 (0.022) | 0.935 | 9 | 0.005 (0.021) | 0.803 |
| MMP12 | Alzheimer’s disease, *FinnGen 2023* | Maximum-likelihood | 4 | -0.001 (0.022) | 0.981 | 9 | 0.010 (0.020) | 0.620 |
| MMP12 | Alzheimer’s disease, *FinnGen 2023* | Weighted Mode | 4 | -0.003 (0.024) | 0.924 | 9 | 0.002 (0.022) | 0.938 |
| MMP12 | Alzheimer’s disease, *FinnGen 2023* | Leave-one-out | 4 | -0.001 (0.022) | 0.981 | 9 | 0.010 (0.020) | 0.620 |
| MMP12 | All-cause dementia, *FinnGen 2023* | Inverse variant weighting | 4 | -0.002 (0.020) | 0.923 | 9 | 0.010 (0.019) | 0.586 |
| MMP12 | All-cause dementia, *FinnGen 2023* | MR-Egger | 4 | -0.028 (0.031) | 0.462 | 9 | -0.018 (0.031) | 0.568 |
| MMP12 | All-cause dementia, *FinnGen 2023* | Weighted Median | 4 | -0.006 (0.021) | 0.788 | 9 | 0.001 (0.020) | 0.942 |
| MMP12 | All-cause dementia, *FinnGen 2023* | Maximum-likelihood | 4 | -0.002 (0.020) | 0.923 | 9 | 0.010 (0.019) | 0.584 |
| MMP12 | All-cause dementia, *FinnGen 2023* | Weighted Mode | 4 | -0.006 (0.021) | 0.808 | 9 | 0.000 (0.020) | 0.992 |
| MMP12 | All-cause dementia, *FinnGen 2023* | Leave-one-out | 4 | -0.002 (0.020) | 0.923 | 9 | 0.010 (0.019) | 0.586 |
| MMP12 | Vascular dementia, *FinnGen 2023* | Inverse variant weighting | 4 | -0.033 (0.049) | 0.499 | 9 | 0.004 (0.045) | 0.930 |
| MMP12 | Vascular dementia, *FinnGen 2023* | MR-Egger | 4 | 0.025 (0.076) | 0.771 | 9 | -0.071 (0.075) | 0.371 |
| MMP12 | Vascular dementia, *FinnGen 2023* | Weighted Median | 4 | -0.025 (0.051) | 0.627 | 9 | -0.010 (0.048) | 0.830 |
| MMP12 | Vascular dementia, *FinnGen 2023* | Maximum-likelihood | 4 | -0.033 (0.049) | 0.498 | 9 | 0.004 (0.045) | 0.930 |
| MMP12 | Vascular dementia, *FinnGen 2023* | Weighted Mode | 4 | -0.022 (0.049) | 0.686 | 9 | -0.018 (0.051) | 0.736 |
| MMP12 | Vascular dementia, *FinnGen 2023* | Leave-one-out | 4 | -0.033 (0.049) | 0.499 | 9 | 0.004 (0.045) | 0.930 |

*MR, Mendelian randomization; SNP, single nucleotide polymorphism; SE, standard error.*

**Supplementary Table 6. Enrichment analysis for the identified proteins.**

| **Term** | **P value** | **FDR-corrected P value** | **Genes** | **dataset** |
| --- | --- | --- | --- | --- |
| Calcium Ion Binding (GO:0005509) | 0.214 | 0.242 | MMP12 | GO_MF |
| Core Promoter Sequence-Specific DNA Binding (GO:0001046) | 0.029 | 0.144 | MMP12 | GO_MF |
| DNA Binding (GO:0003677) | 0.071 | 0.152 | MMP12 | GO_MF |
| Endopeptidase Activity (GO:0004175) | 0.201 | 0.242 | MMP12 | GO_MF |
| Metal Ion Binding (GO:0046872) | 0.249 | 0.249 | MMP12 | GO_MF |
| Metalloendopeptidase Activity (GO:0004222) | 0.112 | 0.186 | MMP12 | GO_MF |
| Metallopeptidase Activity (GO:0008237) | 0.138 | 0.207 | MMP12 | GO_MF |
| Protein Serine/Threonine Kinase Activity (GO:0004674) | 0.029 | 0.144 | RPS6KB1 | GO_MF |
| Protein Serine/Threonine/Tyrosine Kinase Activity (GO:0004712) | 0.014 | 0.144 | RPS6KB1 | GO_MF |
| Sequence-Specific DNA Binding (GO:0043565) | 0.057 | 0.152 | MMP12 | GO_MF |
| Serine-Type Endopeptidase Activity (GO:0004252) | 0.071 | 0.152 | MMP12 | GO_MF |
| Serine-Type Peptidase Activity (GO:0008236) | 0.085 | 0.159 | MMP12 | GO_MF |
| Transcription Cis-Regulatory Region Binding (GO:0000976) | 0.043 | 0.152 | MMP12 | GO_MF |
| Transition Metal Ion Binding (GO:0046914) | 0.226 | 0.242 | MMP12 | GO_MF |
| Zinc Ion Binding (GO:0008270) | 0.201 | 0.242 | MMP12 | GO_MF |
| Brain - Cortex Female 50-59 Up | 0.014 | 0.042 | NEFL | GTEx |
| Brain - Cortex Male 30-39 Up | 0.014 | 0.042 | NEFL | GTEx |
| Brain - Cortex Male 70-79 Up | 0.014 | 0.042 | NEFL | GTEx |
| Brain - Frontal Cortex (BA9) Female 40-49 Up | 0.029 | 0.042 | NEFL | GTEx |
| Brain - Frontal Cortex (BA9) Female 50-59 Up | 0.029 | 0.042 | NEFL | GTEx |
| Brain - Frontal Cortex (BA9) Female 60-69 Up | 0.029 | 0.042 | NEFL | GTEx |
| Brain - Frontal Cortex (BA9) Male 20-29 Up | 0.014 | 0.042 | NEFL | GTEx |
| Brain - Frontal Cortex (BA9) Male 40-49 Up | 0.014 | 0.042 | NEFL | GTEx |
| Brain - Frontal Cortex (BA9) Male 50-59 Up | 0.014 | 0.042 | NEFL | GTEx |
| Brain - Frontal Cortex (BA9) Male 60-69 Up | 0.014 | 0.042 | NEFL | GTEx |
| Brain - Frontal Cortex (BA9) Male 70-79 Up | 0.014 | 0.042 | NEFL | GTEx |
| Brain - Hypothalamus Male 20-29 Up | 0.029 | 0.042 | NEFL | GTEx |
| Brain - Substantia Nigra Female 50-59 Up | 0.029 | 0.042 | NEFL | GTEx |
| Brain - Substantia Nigra Male 20-29 Up | 0.029 | 0.042 | NEFL | GTEx |
| Brain - Substantia Nigra Male 50-59 Up | 0.029 | 0.042 | NEFL | GTEx |
| Cells - Cultured Fibroblasts Female 40-49 Up | 0.057 | 0.062 | EDA2R | GTEx |
| Cells - Cultured Fibroblasts Female 50-59 Up | 0.057 | 0.062 | EDA2R | GTEx |
| Cells - Cultured Fibroblasts Male 20-29 Up | 0.071 | 0.071 | EDA2R | GTEx |
| Cells - Cultured Fibroblasts Male 40-49 Up | 0.057 | 0.062 | EDA2R | GTEx |
| Cells - Cultured Fibroblasts Male 50-59 Up | 0.057 | 0.062 | EDA2R | GTEx |
| Cells - Cultured Fibroblasts Male 70-79 Up | 0.071 | 0.071 | EDA2R | GTEx |
| Kidney - Cortex Female 60-69 Up | 0.014 | 0.042 | HAVCR1 | GTEx |
| Kidney - Cortex Male 30-39 Up | 0.029 | 0.042 | HAVCR1 | GTEx |
| Kidney - Cortex Male 70-79 Up | 0.029 | 0.042 | HAVCR1 | GTEx |
| Minor Salivary Gland Male 20-29 Up | 0.057 | 0.062 | MMP12 | GTEx |
| Small Intestine - Terminal Ileum Female 20-29 Up | 0.043 | 0.059 | MMP12 | GTEx |
| Bosutinib | 0.125 | 0.125 | RPS6KB1 | IDG_DT |
| Crizotinib | 0.125 | 0.125 | RPS6KB1 | IDG_DT |
| Fedratinib | 0.112 | 0.125 | RPS6KB1 | IDG_DT |
| Midostaurin | 0.125 | 0.125 | RPS6KB1 | IDG_DT |
| Nintedanib | 0.125 | 0.125 | RPS6KB1 | IDG_DT |
| Quercetin | 0.098 | 0.125 | MMP12 | IDG_DT |
| Ruboxistaurin | 0.029 | 0.125 | RPS6KB1 | IDG_DT |
| Sunitinib | 0.125 | 0.125 | RPS6KB1 | IDG_DT |
| Vandetanib | 0.085 | 0.125 | RPS6KB1 | IDG_DT |
| AMPK signaling pathway | 0.057 | 0.083 | RPS6KB1 | KEGG |
| Acute myeloid leukemia | 0.014 | 0.083 | RPS6KB1 | KEGG |
| Amyotrophic lateral sclerosis | 0.029 | 0.083 | NEFL | KEGG |
| Apelin signaling pathway | 0.014 | 0.083 | RPS6KB1 | KEGG |
| Autophagy | 0.029 | 0.083 | RPS6KB1 | KEGG |
| Breast cancer | 0.043 | 0.083 | RPS6KB1 | KEGG |
| Chemical carcinogenesis | 0.057 | 0.083 | RPS6KB1 | KEGG |
| Choline metabolism in cancer | 0.029 | 0.083 | RPS6KB1 | KEGG |
| Colorectal cancer | 0.029 | 0.083 | RPS6KB1 | KEGG |
| Cytokine-cytokine receptor interaction | 0.371 | 0.371 | EDA2R | KEGG |
| ErbB signaling pathway | 0.057 | 0.083 | RPS6KB1 | KEGG |
| Fc gamma R-mediated phagocytosis | 0.043 | 0.083 | RPS6KB1 | KEGG |
| Gastric cancer | 0.071 | 0.091 | RPS6KB1 | KEGG |
| HIF-1 signaling pathway | 0.057 | 0.083 | RPS6KB1 | KEGG |
| Hepatocellular carcinoma | 0.029 | 0.083 | RPS6KB1 | KEGG |
| Human cytomegalovirus infection | 0.071 | 0.091 | RPS6KB1 | KEGG |
| Human immunodeficiency virus 1 infection | 0.057 | 0.083 | RPS6KB1 | KEGG |
| Human papillomavirus infection | 0.057 | 0.083 | RPS6KB1 | KEGG |
| Insulin resistance | 0.043 | 0.083 | RPS6KB1 | KEGG |
| Insulin signaling pathway | 0.029 | 0.083 | RPS6KB1 | KEGG |
| Longevity regulating pathway | 0.043 | 0.083 | RPS6KB1 | KEGG |
| NF-kappa B signaling pathway | 0.085 | 0.100 | EDA2R | KEGG |
| PD-L1 expression and PD-1 checkpoint pathway in cancer | 0.057 | 0.083 | RPS6KB1 | KEGG |
| PI3K-Akt signaling pathway | 0.226 | 0.233 | RPS6KB1 | KEGG |
| Pancreatic cancer | 0.014 | 0.083 | RPS6KB1 | KEGG |
| Pathways in cancer | 0.189 | 0.202 | RPS6KB1 | KEGG |
| Pathways of neurodegeneration | 0.085 | 0.100 | NEFL | KEGG |
| Proteoglycans in cancer | 0.098 | 0.108 | RPS6KB1 | KEGG |
| Shigellosis | 0.071 | 0.091 | RPS6KB1 | KEGG |
| TGF-beta signaling pathway | 0.098 | 0.108 | RPS6KB1 | KEGG |
| Thermogenesis | 0.057 | 0.083 | RPS6KB1 | KEGG |
| mTOR signaling pathway | 0.057 | 0.083 | RPS6KB1 | KEGG |
| LY2584702 Down | 0.014 | 0.014 | RPS6KB1 | PDA |
| Activation Of NMDA Receptors and Postsynaptic Events R-HSA-442755 | 0.043 | 0.076 | NEFL | REACTOME |
| Assembly And Cell Surface Presentation of NMDA Receptors R-HSA-9609736 | 0.014 | 0.060 | NEFL | REACTOME |
| CREB1 Phosphorylation Thru NMDA Receptor-Mediated Activation of RAS Signaling R-HSA-442742 | 0.014 | 0.060 | NEFL | REACTOME |
| Cardiac Conduction R-HSA-5576891 | 0.014 | 0.060 | MMP12 | REACTOME |
| Collagen Degradation R-HSA-1442490 | 0.029 | 0.071 | MMP12 | REACTOME |
| Cytokine Signaling in Immune System R-HSA-1280215 | 0.339 | 0.358 | EDA2R | REACTOME |
| Degradation Of Extracellular Matrix R-HSA-1474228 | 0.085 | 0.125 | MMP12 | REACTOME |
| Disease R-HSA-1643685 | 0.381 | 0.381 | HAVCR1 | REACTOME |
| Early SARS-CoV-2 Infection Events R-HSA-9772572 | 0.014 | 0.060 | HAVCR1 | REACTOME |
| Extracellular Matrix Organization R-HSA-1474244 | 0.189 | 0.226 | MMP12 | REACTOME |
| Immune System R-HSA-168256 | 0.196 | 0.226 | MMP12; EDA2R | REACTOME |
| Infectious Disease R-HSA-5663205 | 0.189 | 0.226 | HAVCR1 | REACTOME |
| Innate Immune System R-HSA-168249 | 0.339 | 0.358 | MMP12 | REACTOME |
| Long-term Potentiation R-HSA-9620244 | 0.029 | 0.071 | NEFL | REACTOME |
| MAPK Family Signaling Cascades R-HSA-5683057 | 0.176 | 0.226 | NEFL | REACTOME |
| MAPK1/MAPK3 Signaling R-HSA-5684996 | 0.176 | 0.226 | NEFL | REACTOME |
| MTOR Signaling R-HSA-165159 | 0.043 | 0.076 | RPS6KB1 | REACTOME |
| Metabolism Of Angiotensinogen to Angiotensin R-HSA-2022377 | 0.029 | 0.071 | MMP12 | REACTOME |
| Metabolism Of Proteins R-HSA-392499 | 0.360 | 0.370 | MMP12 | REACTOME |
| Muscle Contraction R-HSA-397014 | 0.014 | 0.060 | MMP12 | REACTOME |
| Negative Regulation of NMDA Receptor-Mediated Neuronal Transmission R-HSA-9617324 | 0.014 | 0.060 | NEFL | REACTOME |
| Neuronal System R-HSA-112316 | 0.071 | 0.114 | NEFL | REACTOME |
| Neurotransmitter Receptors and Postsynaptic Signal Transmission R-HSA-112314 | 0.043 | 0.076 | NEFL | REACTOME |
| Neutrophil Degranulation R-HSA-6798695 | 0.214 | 0.239 | MMP12 | REACTOME |
| Peptide Hormone Metabolism R-HSA-2980736 | 0.043 | 0.076 | MMP12 | REACTOME |
| Physiological Factors R-HSA-5578768 | 0.014 | 0.060 | MMP12 | REACTOME |
| Post NMDA Receptor Activation Events R-HSA-438064 | 0.043 | 0.076 | NEFL | REACTOME |
| RAF/MAP Kinase Cascade R-HSA-5673001 | 0.164 | 0.226 | NEFL | REACTOME |
| Ras Activation Upon Ca2+ Influx Thru NMDA Receptor R-HSA-442982 | 0.014 | 0.060 | NEFL | REACTOME |
| SARS-CoV Infections R-HSA-9679506 | 0.029 | 0.071 | HAVCR1 | REACTOME |
| SARS-CoV-2 Infection R-HSA-9694516 | 0.029 | 0.071 | HAVCR1 | REACTOME |
| Signal Transduction R-HSA-162582 | 0.190 | 0.226 | RPS6KB1; NEFL | REACTOME |
| TNFR2 Non-Canonical NF-kB Pathway R-HSA-5668541 | 0.085 | 0.125 | EDA2R | REACTOME |
| TNFs Bind Their Physiological Receptors R-HSA-5669034 | 0.029 | 0.071 | EDA2R | REACTOME |
| Transmission Across Chemical Synapses R-HSA-112315 | 0.057 | 0.096 | NEFL | REACTOME |
| Unblocking Of NMDA Receptors, Glutamate Binding and Activation R-HSA-438066 | 0.014 | 0.060 | NEFL | REACTOME |
| mTORC1-mediated Signaling R-HSA-166208 | 0.043 | 0.076 | RPS6KB1 | REACTOME |

*GO_MF, Gene Ontology – Molecular Function; GTEx, Genotype-Tissue Expression; IDG_DT, Illuminating the Druggable Genome – drug target; PDA, Proteomics Drug Atlas; KEGG, Kyoto Encyclopedia of Genes and Genomes; REACTOME, Reactome Pathway Database; FDR, false discovery rate.*

**Supplementary Table 7. Drugs linked to the identified proteins associated with dementia, drug type, their potential mechanisms of action, current target disease, and the status and phase of the trials.**

| **Drug** | **Gene** | **Type** | **Mechanism Of Action** | **Disease** | **Phase** | **Status** |
| --- | --- | --- | --- | --- | --- | --- |
| LY-2780301 | *RPS6KB1* | Small molecule | Ribosomal protein S6 kinase (P70S6K) inhibitor | lymphoma | Phase I | Completed |
| LY-2584702 | *RPS6KB1* | Small molecule | Ribosomal protein S6 kinase 1 inhibitor | renal cell carcinoma | Phase I | Terminated |
| LY-2584702 | *RPS6KB1* | Small molecule | Ribosomal protein S6 kinase 1 inhibitor | cancer | Phase I | Completed |
| LY-2584702 | *RPS6KB1* | Small molecule | Ribosomal protein S6 kinase 1 inhibitor | non-small cell lung carcinoma | Phase I | Terminated |
| LY-2584702 | *RPS6KB1* | Small molecule | Ribosomal protein S6 kinase 1 inhibitor | metastasis | Phase I | Terminated |
| XL-418 | *RPS6KB1* | Small molecule | Ribosomal protein S6 kinase (P70S6K) inhibitor | cancer | Phase I | Suspended |
| LY-2584702 | *RPS6KB1* | Small molecule | Ribosomal protein S6 kinase 1 inhibitor | cancer | Phase I | Terminated |
| LY-2584702 | *RPS6KB1* | Small molecule | Ribosomal protein S6 kinase 1 inhibitor | neuroendocrine neoplasm | Phase I | Terminated |
| TAS0612 | *RPS6KB1* | Small molecule | Ribosomal protein S6 kinase (P70S6K) inhibitor | neoplasm | Phase I | Recruiting |
| LY-2780301 | *RPS6KB1* | Small molecule | Ribosomal protein S6 kinase (P70S6K) inhibitor | metastasis | Phase I | Completed |
| MSC-2363318A | *RPS6KB1* | Small molecule | Ribosomal protein S6 kinase (P70S6K) inhibitor | neoplasm | Phase I | Completed |
| MARIMASTAT | *MMP12* | Small molecule | Matrix metalloproteinase 12 inhibitor | lung cancer | Phase III | Completed |
| MARIMASTAT | *MMP12* | Small molecule | Matrix metalloproteinase 12 inhibitor | breast cancer | Phase III | Completed |
| CTS-1027 | *MMP12* | Small molecule | Matrix metalloproteinase 12 inhibitor | hepatitis C virus infection | Phase II | Terminated |
| AZD-1236 | *MMP12* | Small molecule | Matrix metalloproteinase 12 inhibitor | cystic fibrosis | Phase II | Withdrawn |
| AZD-1236 | *MMP12* | Small molecule | Matrix metalloproteinase 12 inhibitor | chronic obstructive pulmonary disease | Phase II | Completed |
| CTS-1027 | *MMP12* | Small molecule | Matrix metalloproteinase 12 inhibitor | hepatitis C virus infection | Phase II | Completed |
| CTS-1027 | *MMP12* | Small molecule | Matrix metalloproteinase 12 inhibitor | chronic hepatitis C virus infection | Phase II | Completed |

**Supplementary Table 8. Summary Information on dementia GWAS datasets.**

| **Trait** | **Late-onset Alzheimer’s disease** | **Alzheimer's disease** | **Alzheimer's disease**  **(wide definition)** | **All-cause dementia** | **Vascular dementia** |
| --- | --- | --- | --- | --- | --- |
| **Year** | 2019 | 2022 | 2023 | 2023 | 2023 |
| **Case, n** | 21,982 | 39,106 (clinically diagnosed cases) + 46,828 (proxy cases) | 15,617 | 19,157 | 2,717 |
| **Control, n** | 41,944 | 401,577 | 396,564 | 388,560 | 393,024 |
| **SNP, n** | 10,528,610 | 20,921,626 | 21,306,349 | 21,306,258 | 21,306,039 |
| **Data cohort** | International Genomics of Alzheimer’s Project (IGAP consortium): Alzheimer Disease Genetics Consortium (ADGC), European Alzheimer's Disease Initiative (EADI), Cohorts for Heart and Aging Research in Genomic Epidemiology Consortium (CHARGE), Genetic and Environmental Risk in AD/Defining Genetic, Polygenic and Environmental Risk for Alzheimer's Disease Consortium (GERAD/PERADES) | European Alzheimer & Dementia Biobank (EADB) consortium | FinnGen | FinnGen | FinnGen |
| **Country of origin** | Canada, France, Germany, Greece, Iceland, Netherlands, U.K., U.S. | Belgium, Bulgaria, Czech Republic, Denmark, Finland, France, Germany, Greece, Italy, Netherlands, Norway, Portugal, Spain, Sweden, Switzerland, U.K., U.S. | Finland | Finland | Finland |
| **Case ascertainment** | Clinical assessment, magnetic resonance imaging or autopsy-confirmed, and/or diagnosis from health care records | Clinical diagnosis, proxy cases | Clinical diagnosis from health care records, insurance reimbursement records, medication purchase records | Clinical diagnosis from health care records, insurance reimbursement records, medication purchase records | Clinical diagnosis from health care records |
| **Genetic data** | Genome-wide genotyping, imputation using 1000 Genomes project, phase 2 release | Genome-wide genotyping, Affymetrix, Illumina [21101114] imputed | Illumina GWAS arrays, using Finnish specific WGS reference panel of ~9000 individuals | Illumina GWAS arrays, using Finnish specific WGS reference panel of ~9000 individuals | Illumina GWAS arrays, using Finnish specific WGS reference panel of ~9000 individuals |

*GWAS, genome wide association study; SNP, single-nucleotide polymorphism; WGS, whole genome sequence.*

**Supplementary Figures**

**Supplementary Figure 1. Flow diagram for ELSA proteomics project sample selection and quality control pipeline.**


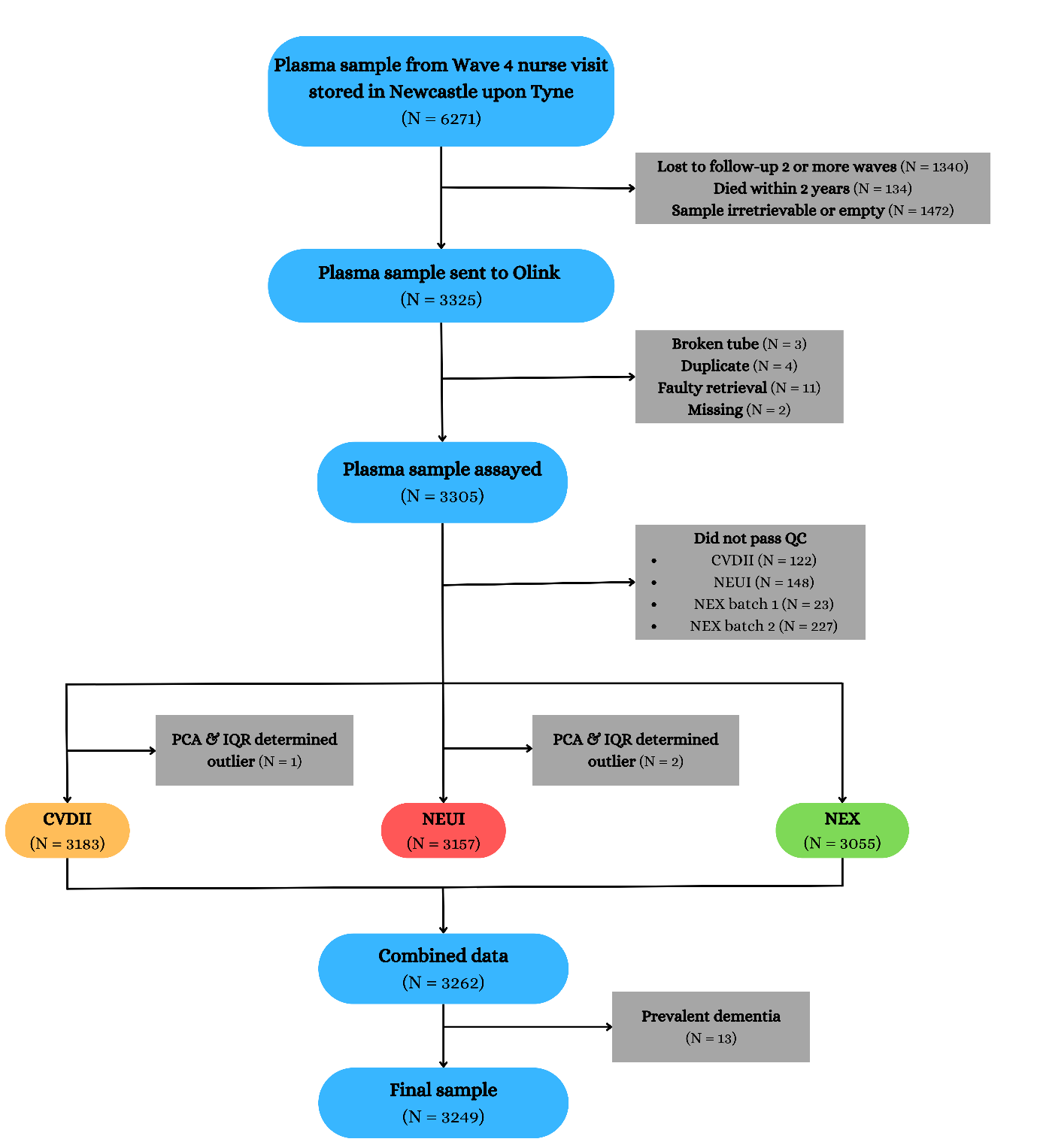


*ELSA, English Longitudinal Study of Ageing; QC, quality control; PCA, principal component analysis; IQR, interquartile range; CVDII, cardiovascular disease 2 Olink panel; NEU, neurology Olink panel; NEX, neurology exploratory Olink panel.*

**Supplementary Figure 2. Dementia cases by data source in ELSA.**


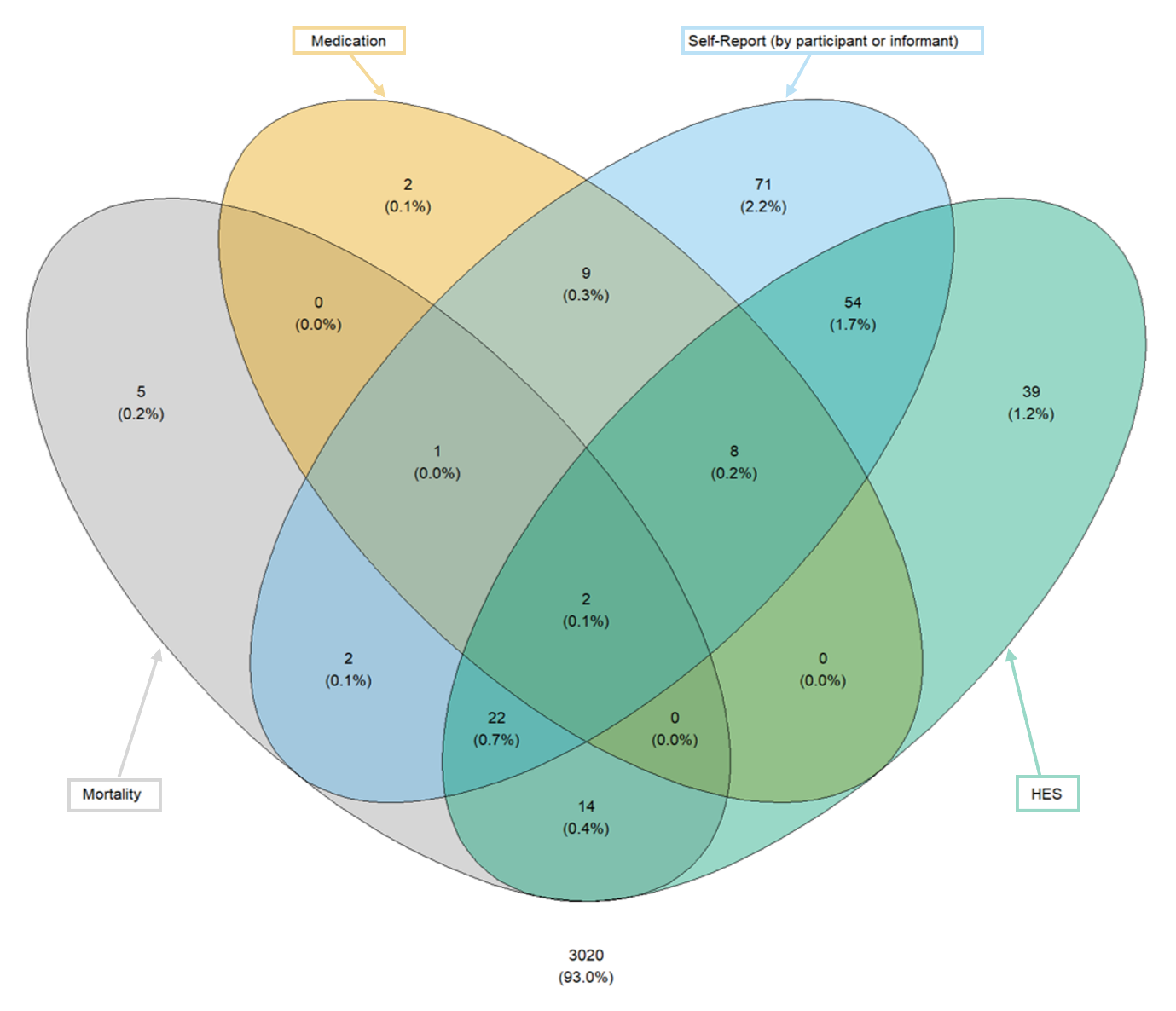


*HES, Hospital Episode Statistics.*

**Supplementary Figure 3. Boxplot for all protein concentration from the Olink Target 96 Cardiovascular II panel in normalized protein expression (NPX) in ELSA by dementia status.**


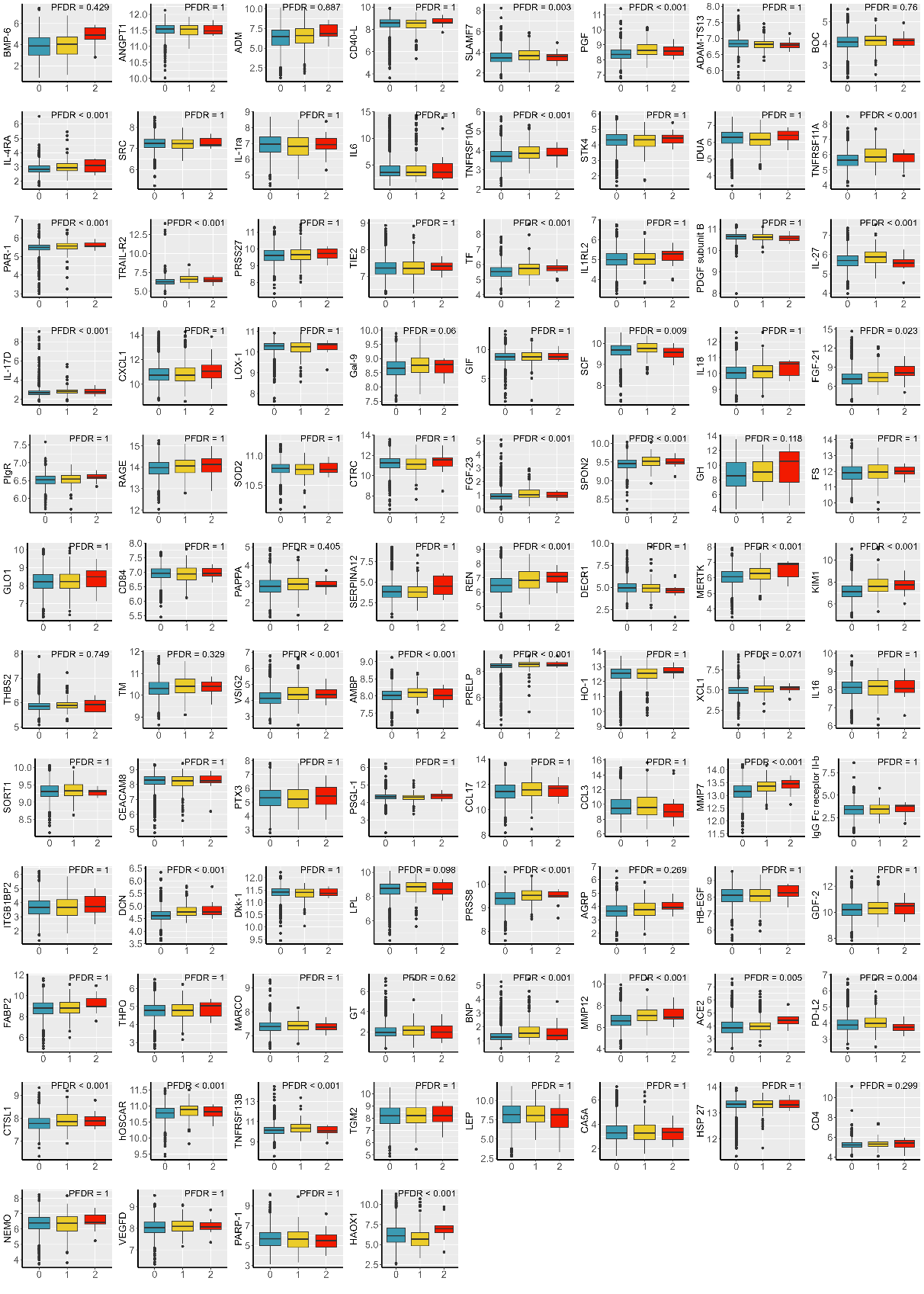


*X-axis: 0 = no dementia (N=3,020); 1 = incident all-cause dementia (N=229), 2 = prevalent dementia (N=13). Y-axis indicates the protein* normalized protein expression (*NPX) values.*

*The center line within the box represents the median of the data. The bottom and top edges of the box correspond to the 25th and 75th percentiles (quartiles), respectively. Extending from the box are "whiskers," which indicate the expected range of data variation. These whiskers span up to 1.5 times the interquartile range (IQR) beyond the upper and lower quartiles. Any dot positioned beyond the whiskers signify outliers — data points that deviate significantly from the expected range.*

*P-values were calculated using the Kruskal-Wallis method to test differences in protein concentrations between groups. To account for multiple comparisons, the False Discovery Rate (FDR) correction was applied, and the adjusted p-values are reported as P_FDR_.*

**Supplementary Figure 4. Boxplot for all protein concentration from the Olink Target 96 Neurology I panel in normalized protein expression (NPX) in ELSA by dementia status.**


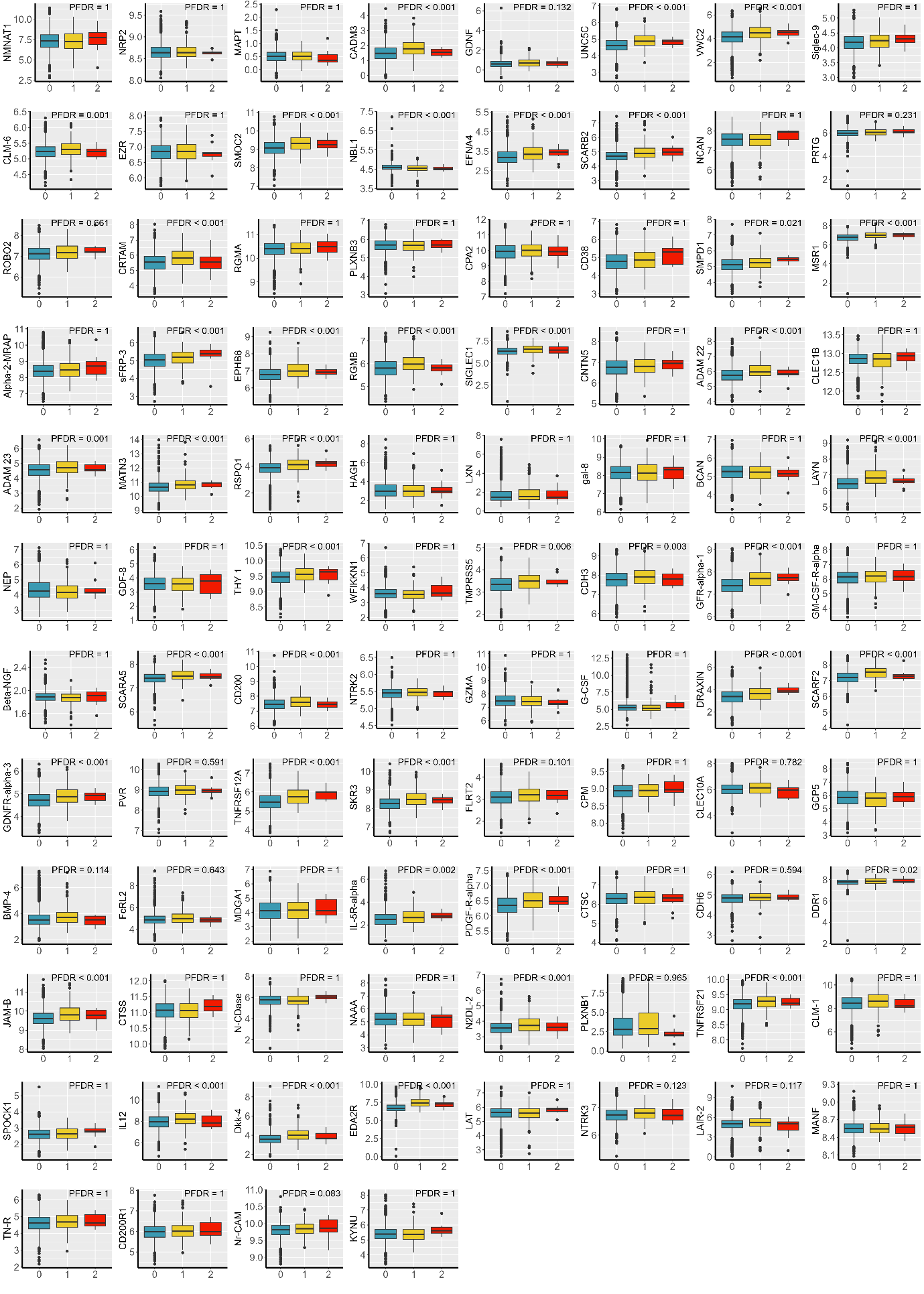


*X-axis: 0 = no dementia (N=3,020); 1 = incident all-cause dementia (N=229), 2 = prevalent dementia (N=13). Y-axis indicates the protein* normalized protein expression (*NPX) values.*

*The center line within the box represents the median of the data. The bottom and top edges of the box correspond to the 25th and 75th percentiles (quartiles), respectively. Extending from the box are "whiskers," which indicate the expected range of data variation. These whiskers span up to 1.5 times the interquartile range (IQR) beyond the upper and lower quartiles. Any dot positioned beyond the whiskers signify outliers — data points that deviate significantly from the expected range.*

*P-values were calculated using the Kruskal-Wallis method to test differences in protein concentrations between groups. To account for multiple comparisons, the False Discovery Rate (FDR) correction was applied, and the adjusted p-values are reported as P_FDR_.*

**Supplementary Figure 5. Boxplot for all protein concentration from the Olink Target 96 Neuro Exploratory panel in normalized protein expression (NPX) in ELSA by dementia status.**


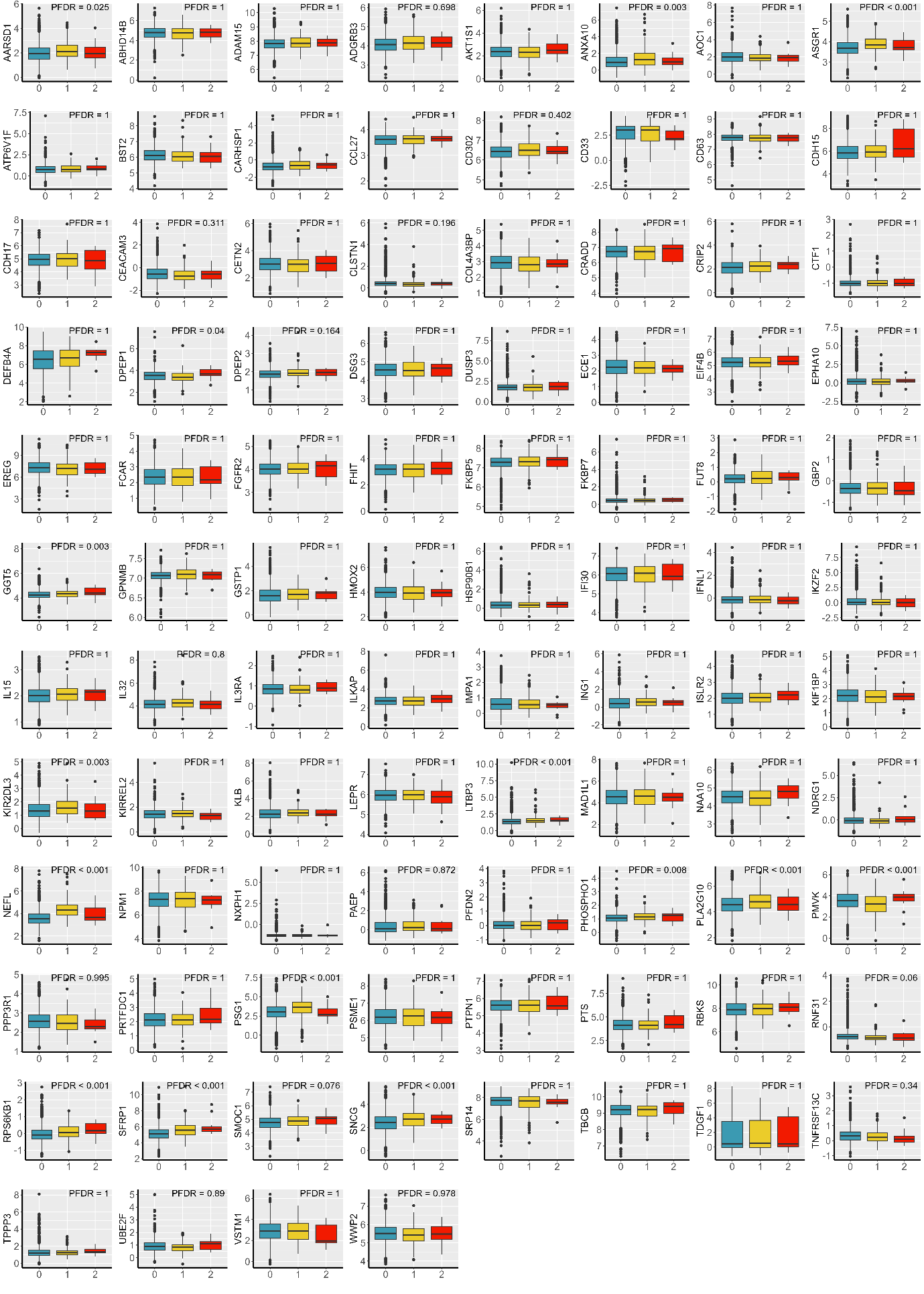


*X-axis: 0 = no dementia (N=3,020); 1 = incident all-cause dementia (N=229), 2 = prevalent dementia (N=13). Y-axis indicates the protein* normalized protein expression (*NPX) values.*

*The center line within the box represents the median of the data. The bottom and top edges of the box correspond to the 25th and 75th percentiles (quartiles), respectively. Extending from the box are "whiskers," which indicate the expected range of data variation. These whiskers span up to 1.5 times the interquartile range (IQR) beyond the upper and lower quartiles. Any dot positioned beyond the whiskers signify outliers — data points that deviate significantly from the expected range.*

*P-values were calculated using the Kruskal-Wallis method to test differences in protein concentrations between groups. To account for multiple comparisons, the False Discovery Rate (FDR) correction was applied, and the adjusted p-values are reported as P_FDR_.*

**Supplementary Figure 6. Volcano plot shows the unadjusted HR (x axis) and two-sided P values (y axis) for the association between protein concentration with incident all-cause dementia using imputed data.**


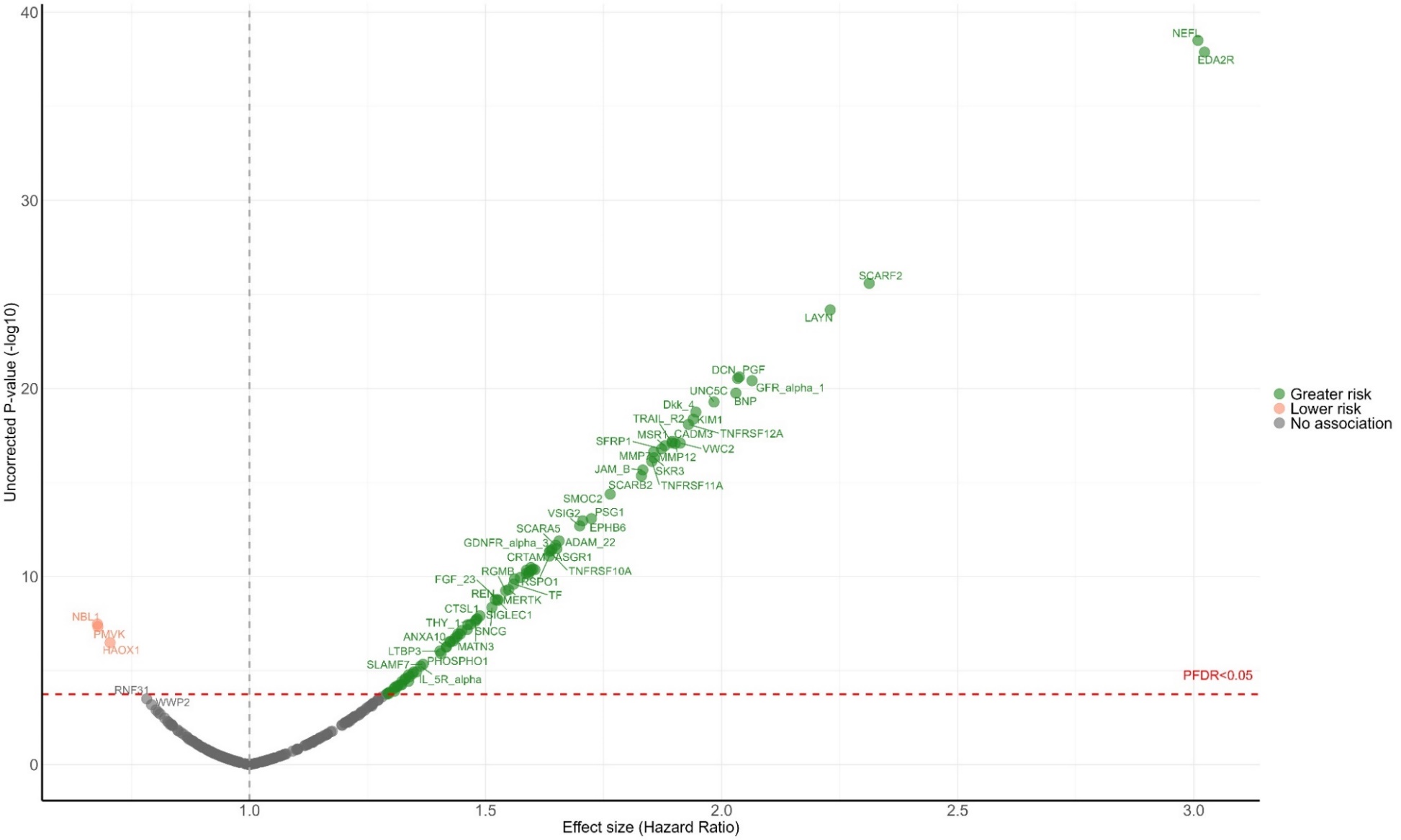


*X-axis displays the unadjusted hazard ratios from Cox Proportional Hazard Regression models, in a sample size of 3249. Y-axis displays the nominal uncorrected p-value (-log_10_). Proteins above the horizontal dotted red line were significantly associated with incident all-cause dementia with FDR-corrected p-value <0.05.*

**Supplementary Figure 7. Volcano plot shows the sex-, age- and ethnicity-adjusted HR (x axis) and two-sided P values (y axis) for the association between protein concentration with incident all-cause dementia using imputed data.**


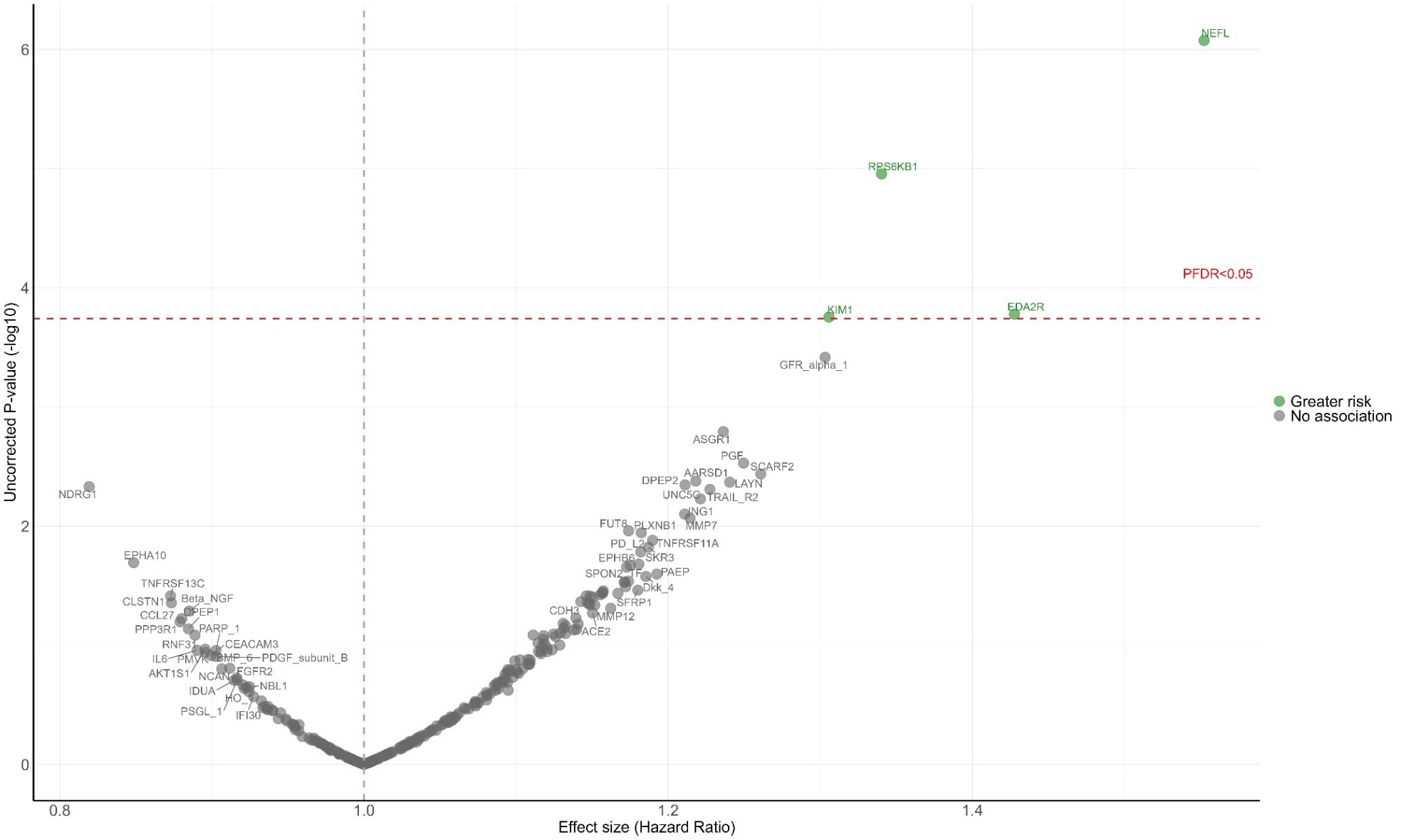


*X-axis displays the hazard ratios from Cox Proportional Hazard Regression models, adjusted for age, sex, and ethnicity, in a sample size of 3249. Y-axis displays the nominal uncorrected p-value (-log_10_). Proteins above the horizontal dotted red line were significantly associated with incident all-cause dementia with FDR-corrected p-value <0.05.*

**Supplementary Figure 8. Volcano plot shows the fully adjusted HR (x axis) and two-sided P values (y axis) for the association between protein concentration with incident all-cause dementia using imputed data from random forest methods.**


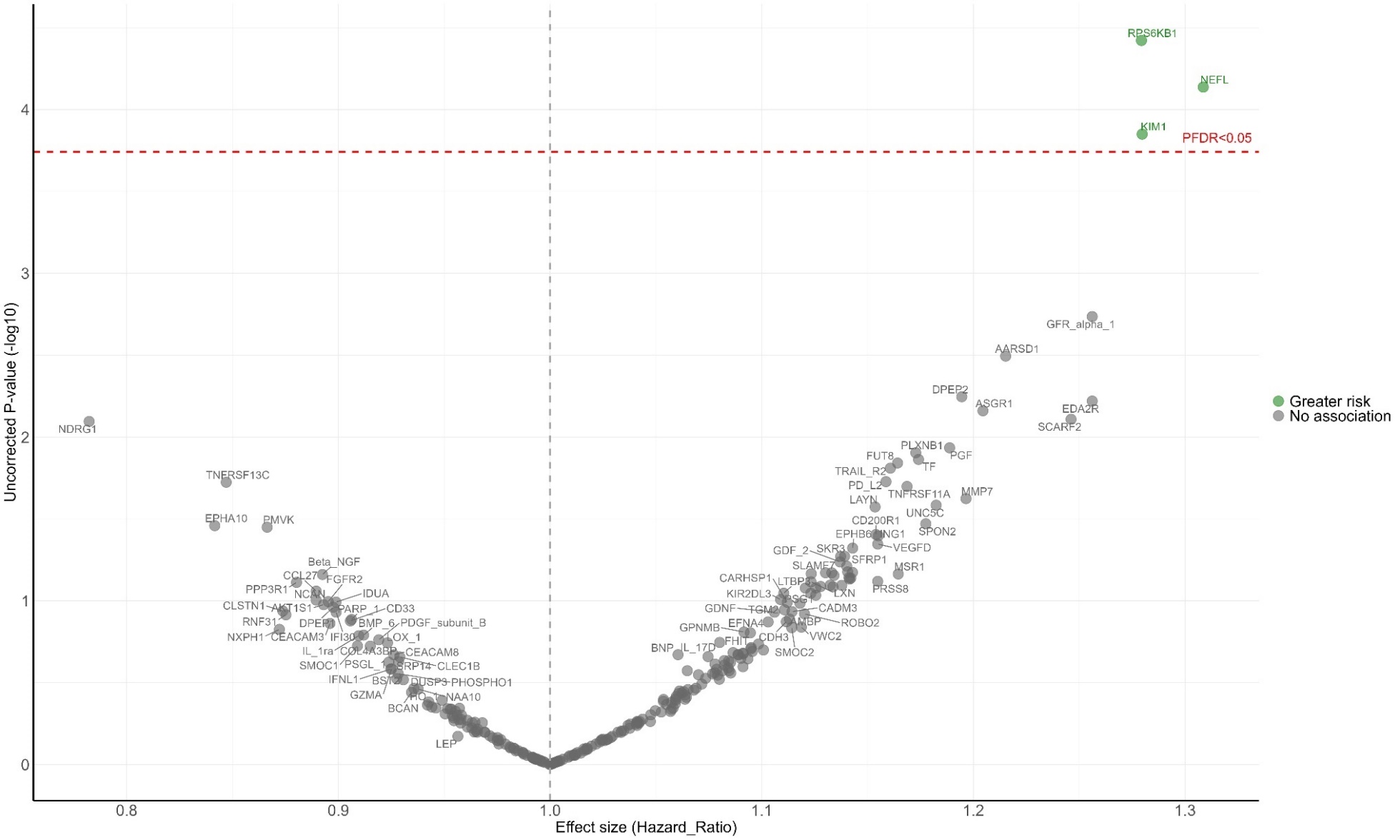


*X-axis displays the hazard ratios from Cox Proportional Hazard Regression models, adjusted for age, sex, education, ethnicity, smoking status, depression, cardiovascular disease, body mass index, systolic blood pressure, LDL cholesterol, in a sample size of 3249. Y-axis displays the nominal uncorrected p-value (-log_10_). Proteins above the horizontal dotted red line were significantly associated with incident all-cause dementia with FDR-corrected p-value <0.05.*

**Supplementary Figure 9. Volcano plot shows the fully adjusted HR (x axis) and two-sided P values (y axis) for the association between protein concentration with incident all-cause dementia, excluding other ethnic groups using imputed data.**


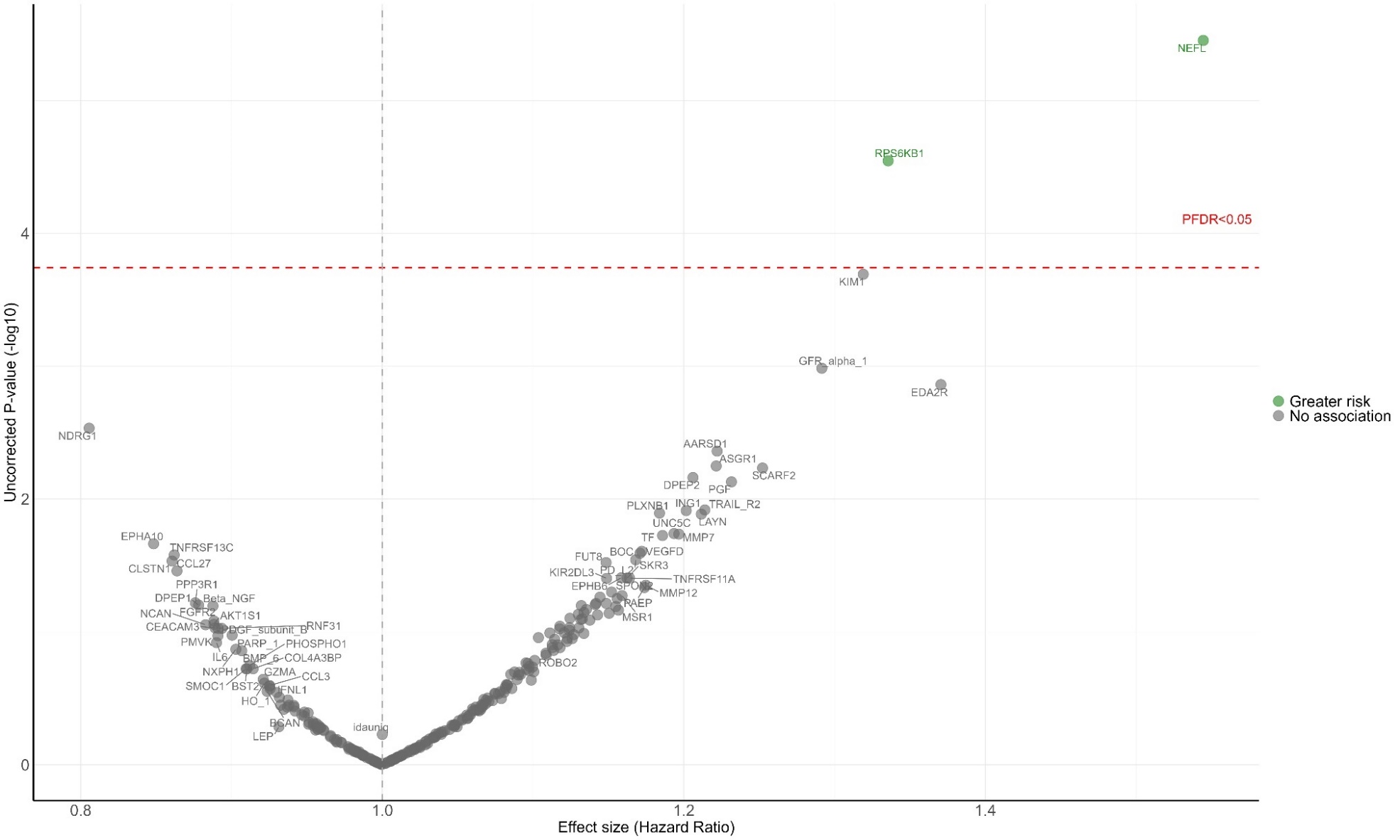


*X-axis displays the hazard ratios from Cox Proportional Hazard Regression models, adjusted for age, sex, education, smoking status, depression, cardiovascular disease, body mass index, systolic blood pressure, LDL cholesterol, in a sample size of 3157. Y-axis displays the nominal uncorrected p-value (-log_10_). Proteins above the horizontal dotted red line were significantly associated with incident all0cause dementia with FDR-corrected p-value <0.05.*

**Supplementary Figure 10. Volcano plot shows the fully adjusted HR (x axis) and two-sided P values (y axis) for the association between protein concentration with incident all-cause dementia, excluding APOE 4 carriers using imputed data.**


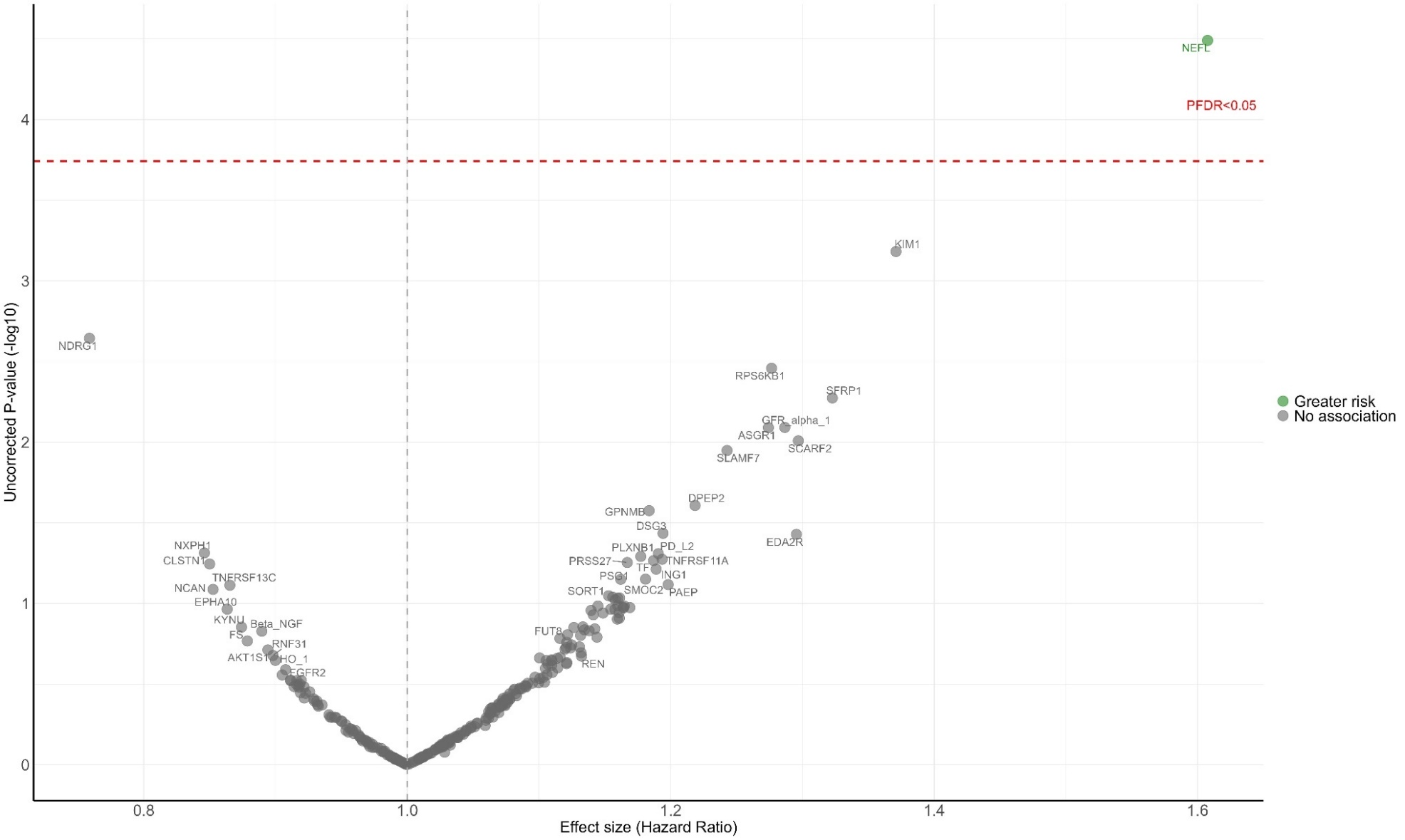


*X-axis displays the hazard ratios from Cox Proportional Hazard Regression models, adjusted for age, sex, education, ethnicity, smoking status, depression, cardiovascular disease, body mass index, systolic blood pressure, LDL cholesterol, in a sample size of 2392. Y-axis displays the nominal uncorrected p-value (-log_10_). Proteins above the horizontal dotted red line were significantly associated with incident all-cause dementia with FDR-corrected p-value <0.05.*

**Supplementary Figure 11. Volcano plot shows the fully adjusted HR (x axis) and two-sided P values (y axis) for the association between protein concentration with incident all-cause dementia, after reducing the possibility of reverse causation bias by excluding all-cause dementia cases that occurred during the first year of follow-up using imputed data.**


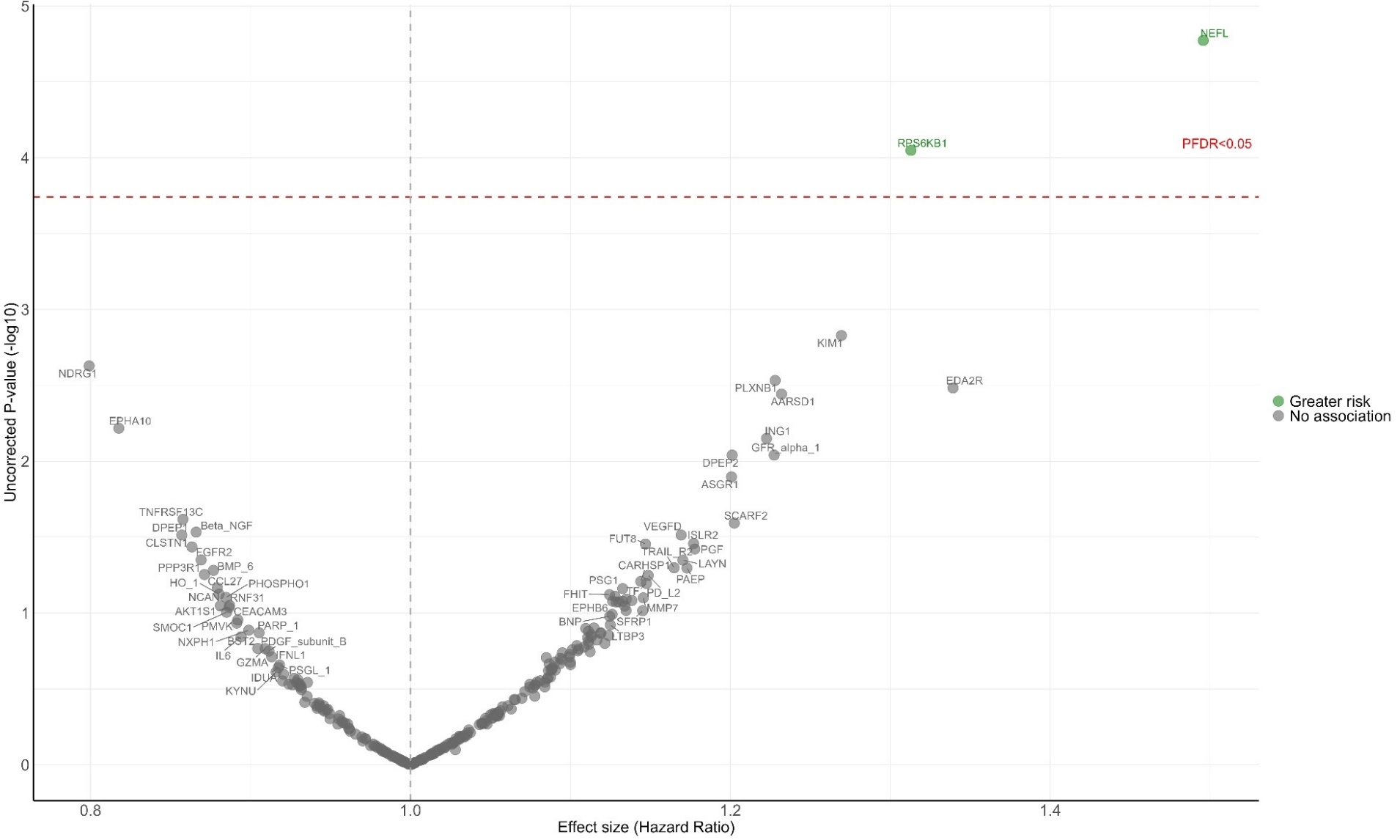


*X-axis displays the hazard ratios from Cox Proportional Hazard Regression models, adjusted for age, sex, education, ethnicity, smoking status, depression, cardiovascular disease, body mass index, systolic blood pressure, LDL cholesterol, in a sample size of 3234. Y-axis displays the nominal uncorrected p-value (-log_10_). Proteins above the horizontal dotted red line were significantly associated with incident all-cause dementia with FDR-corrected p-value <0.05.*

**Supplementary Figure 12. Volcano plot shows the fully adjusted HR (x axis) and two-sided P values (y axis) for the association between protein concentration with incident all-cause dementia, excluding participants <60 years using imputed data.**


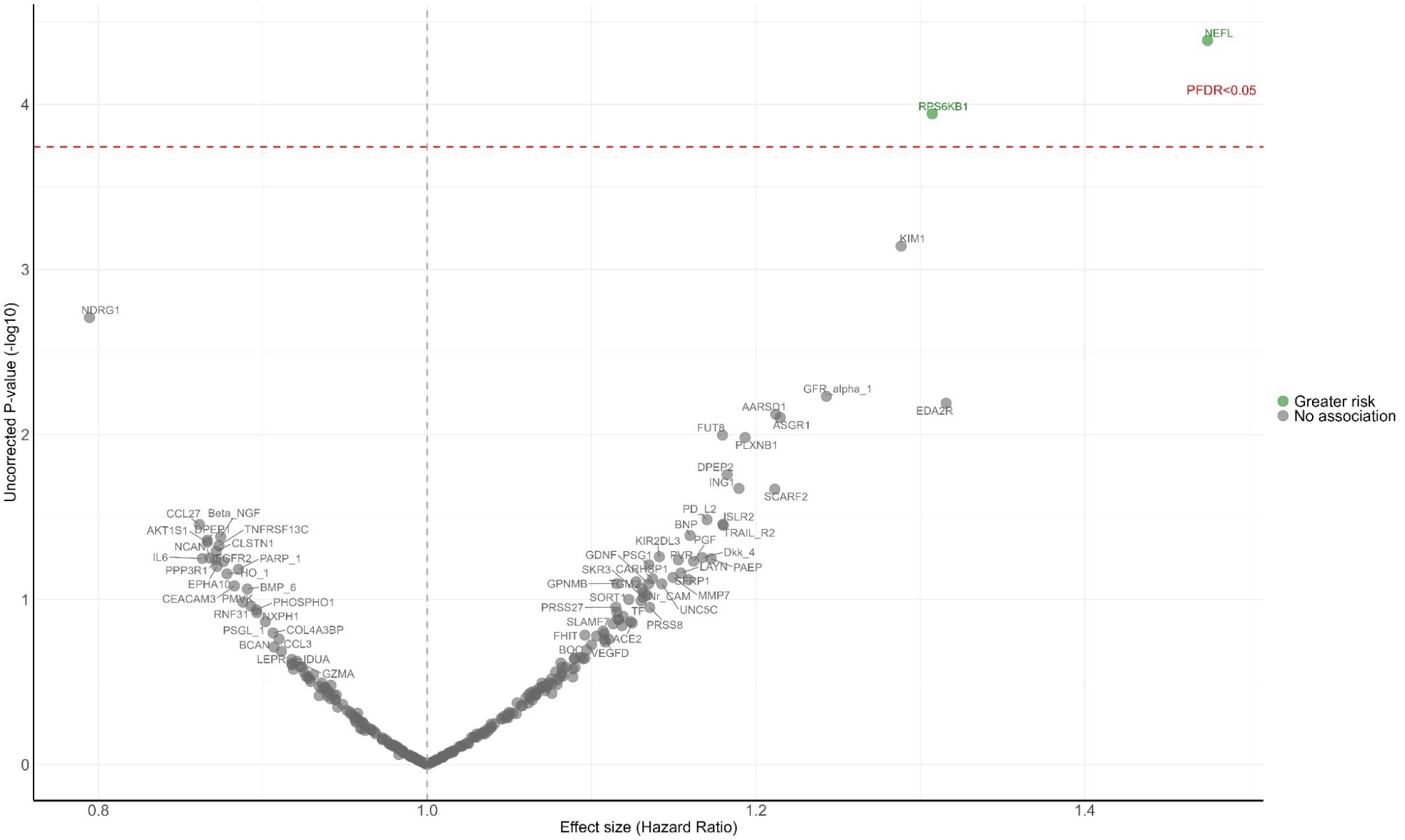


*X-axis displays the hazard ratios from Cox Proportional Hazard Regression models, adjusted for age, sex, education, ethnicity, smoking status, depression, cardiovascular disease, body mass index, systolic blood pressure, LDL cholesterol, in a sample size of 1963. Y-axis displays the nominal uncorrected p-value (-log_10_). Proteins above the horizontal dotted red line were significantly associated with incident all-cause dementia with FDR-corrected p-value <0.05.*

**Supplementary Figure 13. Volcano plot showing the fully adjusted sub-distribution HR (x axis) and two-sided P values (y axis) for the association between protein concentration with incident all-cause dementia, using Fine-Gray competing risk regression using imputed data.**


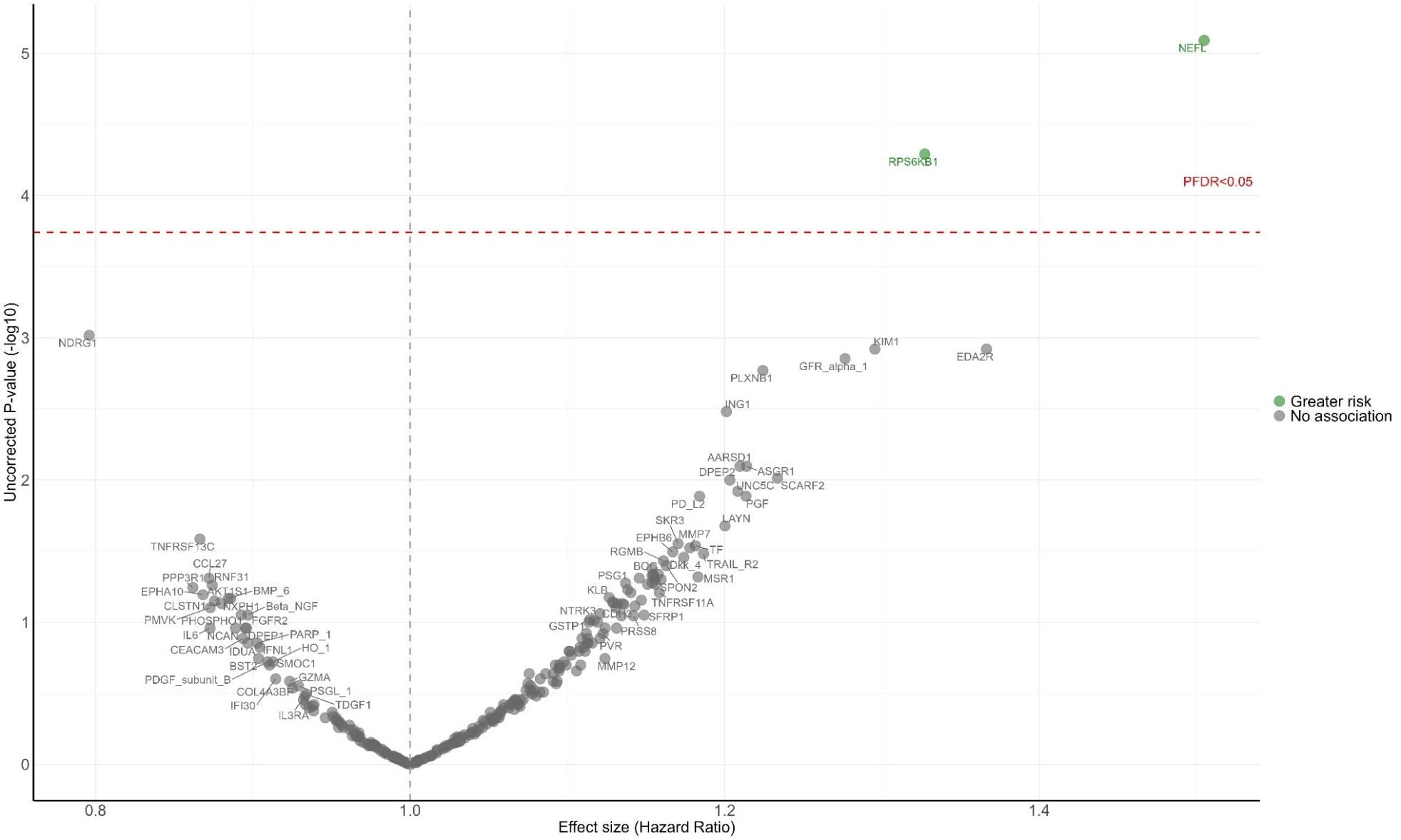


*X-axis displays the hazard ratios from Cox Proportional Hazard Regression models, adjusted for age, sex, education, ethnicity, smoking status, depression, cardiovascular disease, body mass index, systolic blood pressure, LDL cholesterol, in a sample size of 3249. Y-axis displays the nominal uncorrected p-value (-log_10_). Proteins above the horizontal dotted red line were significantly associated with incident all-cause dementia with FDR-corrected p-value <0.05.***Supplementary Figure 14. Volcano plot showing the fully adjusted HR (x axis) and two-sided P values (y axis) for the association between protein concentration with incident Alzheimer’s disease using imputed data.**


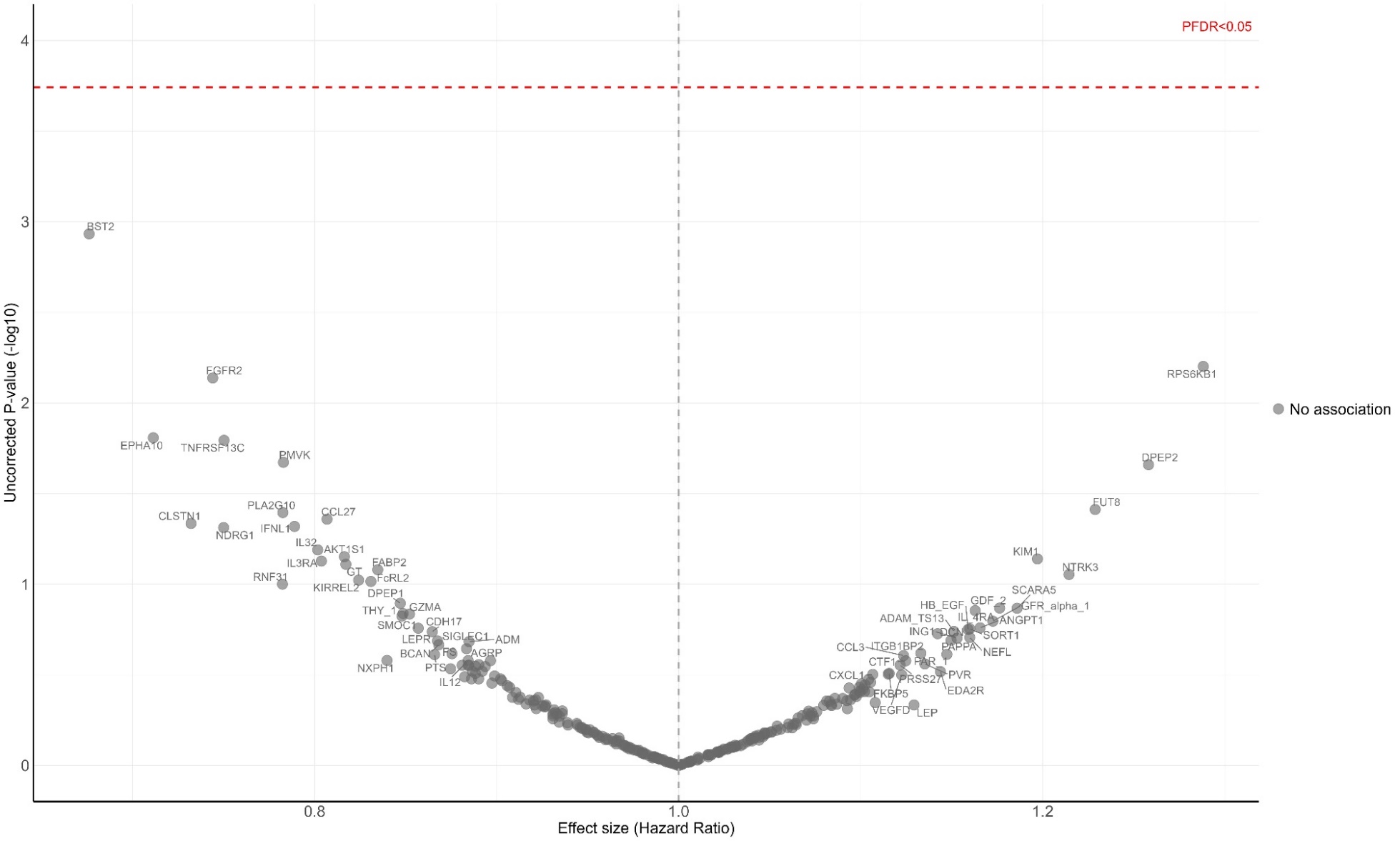


*X-axis displays the hazard ratios from Cox Proportional Hazard Regression models, adjusted for age, sex, education, ethnicity, smoking status, depression, cardiovascular disease, body mass index, systolic blood pressure, LDL cholesterol, in a sample size of 3249. Y-axis displays the nominal uncorrected p-value (-log_10_). Proteins above the horizontal dotted red line were significantly associated with incident Alzheimer’s disease with FDR-corrected p-value <0.05.***Supplementary Figure 15. Volcano plot showing the fully adjusted HR (x axis) and two-sided P values (y axis) for the association between protein concentration with incident vascular dementia using imputed data.**


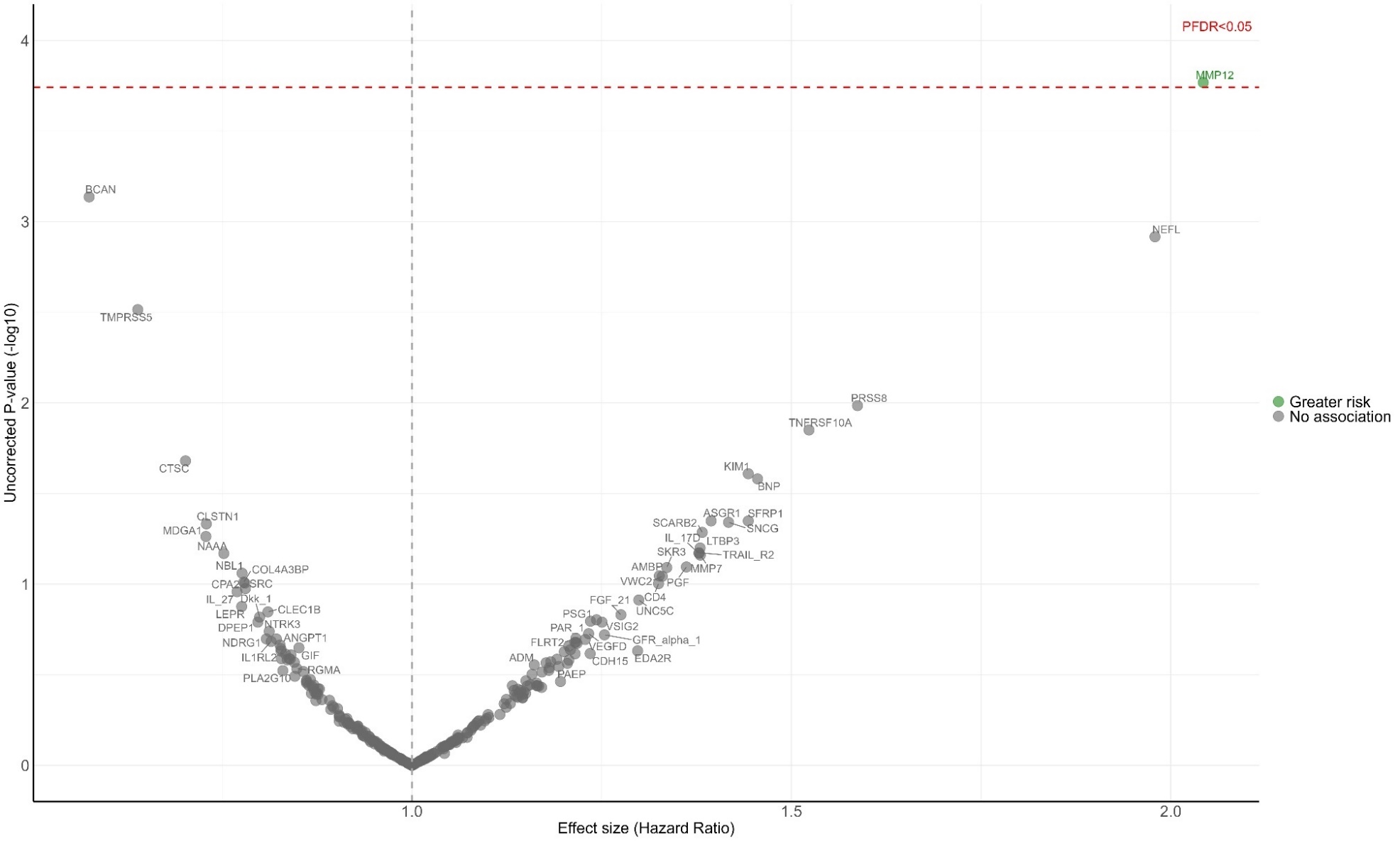


*X-axis displays the hazard ratios from Cox Proportional Hazard Regression models, adjusted for age, sex, education, ethnicity, smoking status, depression, cardiovascular disease, body mass index, systolic blood pressure, LDL cholesterol, in a sample size of 3249. Y-axis displays the nominal uncorrected p-value (-log_10_). Proteins above the horizontal dotted red line were significantly associated with incident vascular dementia with FDR-corrected p-value <0.05.*

**Supplementary Figure 16. Two-sample Mendelian randomization in the forward direction (protein concentration 🡪 dementia) scatter plots for NEFL in five GWAS for Alzheimer’s disease, all-cause dementia, and vascular dementia.**


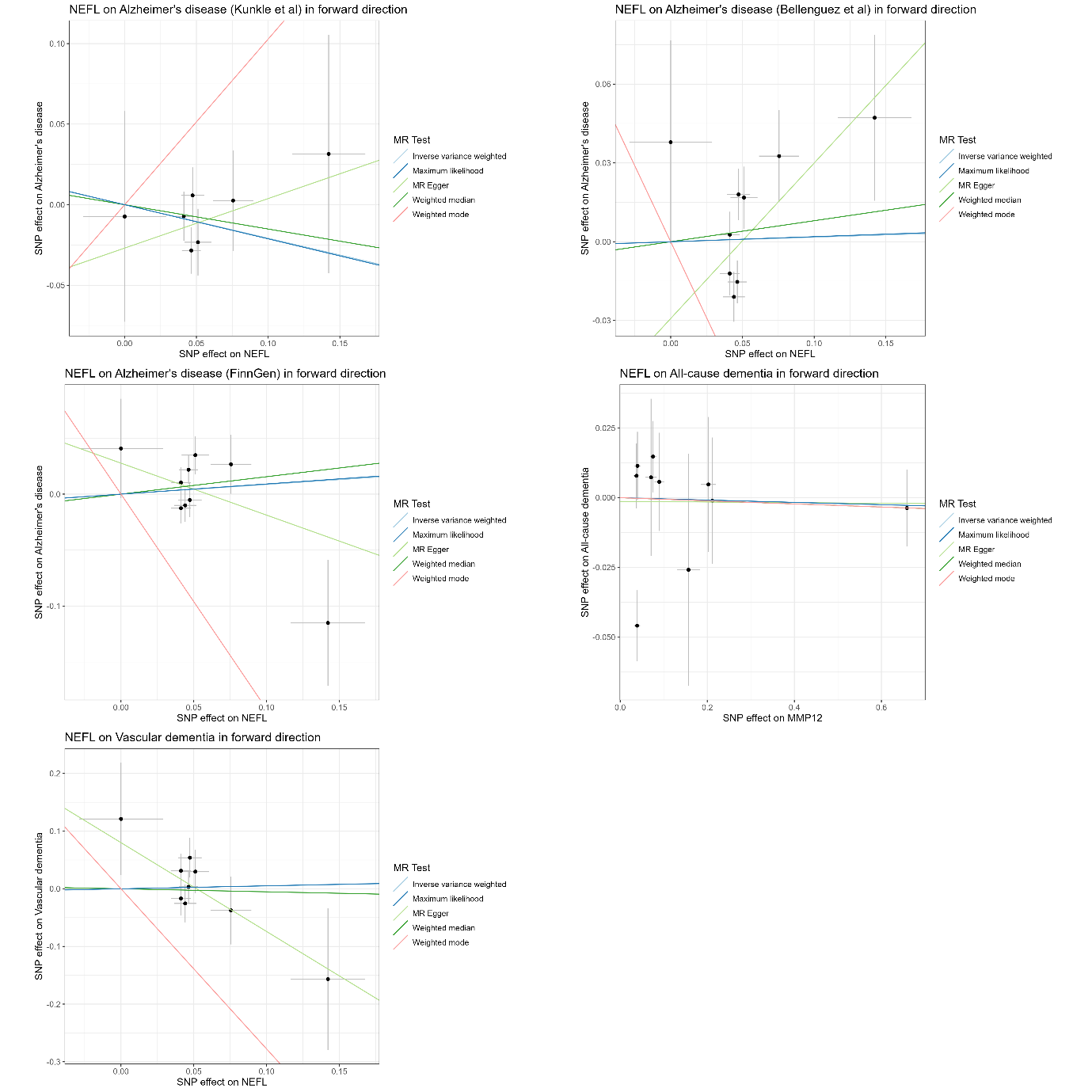


Analyses were conducted using the inverse variant weighted, maximum likelihood, MR-Egger, Weighted median, Weighted mode methods. The slope of each line corresponding to the estimated MR effect per method.

**Supplementary Figure 17. Two-sample Mendelian randomization in the forward direction (protein concentration 🡪 dementia) scatter plots for KIM1 (HAVCR1) in five GWAS for Alzheimer’s disease, all-cause dementia, and vascular dementia.**


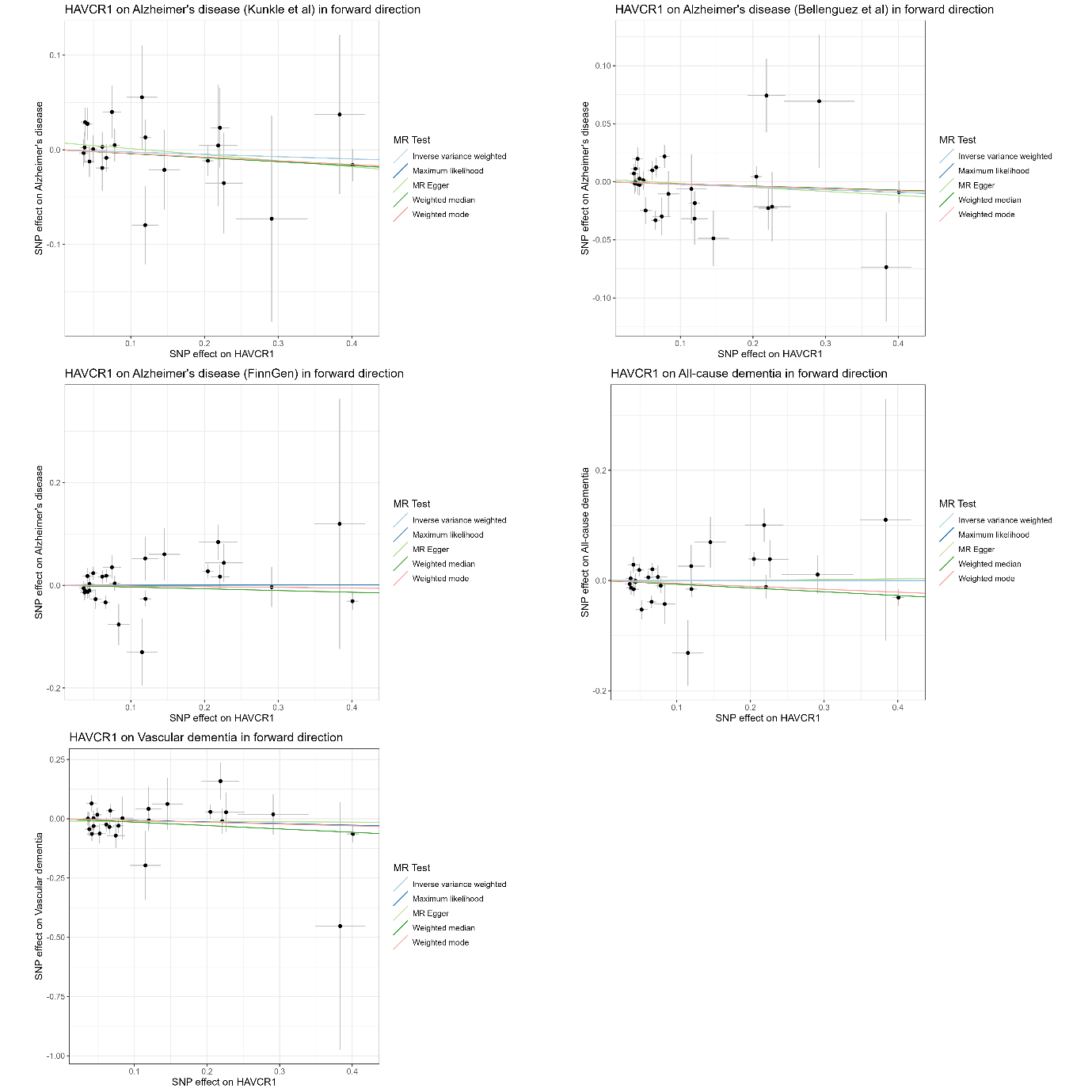


Analyses were conducted using the inverse variant weighted, maximum likelihood, MR-Egger, Weighted median, Weighted mode methods. The slope of each line corresponding to the estimated MR effect per method.

**Supplementary Figure 18. Two-sample Mendelian randomization in the forward direction (protein concentration 🡪 dementia) scatter plots for EDA2R in five GWAS for Alzheimer’s disease, all-cause dementia, and vascular dementia.**


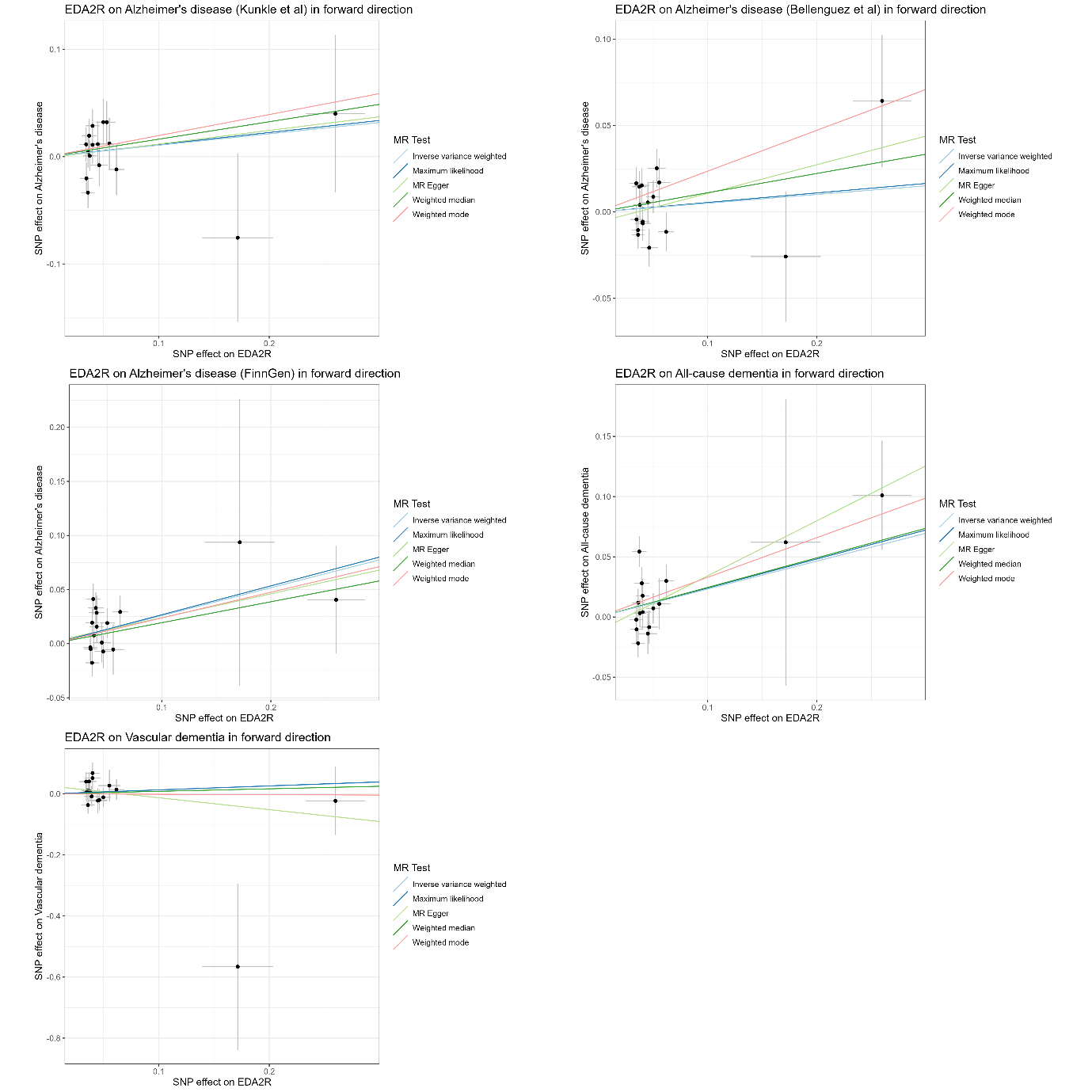


Analyses were conducted using the inverse variant weighted, maximum likelihood, MR-Egger, Weighted median, Weighted mode methods. The slope of each line corresponding to the estimated MR effect per method.

**Supplementary Figure 19. Two-sample Mendelian randomization in the forward direction (protein concentration 🡪 dementia) scatter plots for MMP12 in five GWAS for Alzheimer’s disease, all-cause dementia, and vascular dementia.**


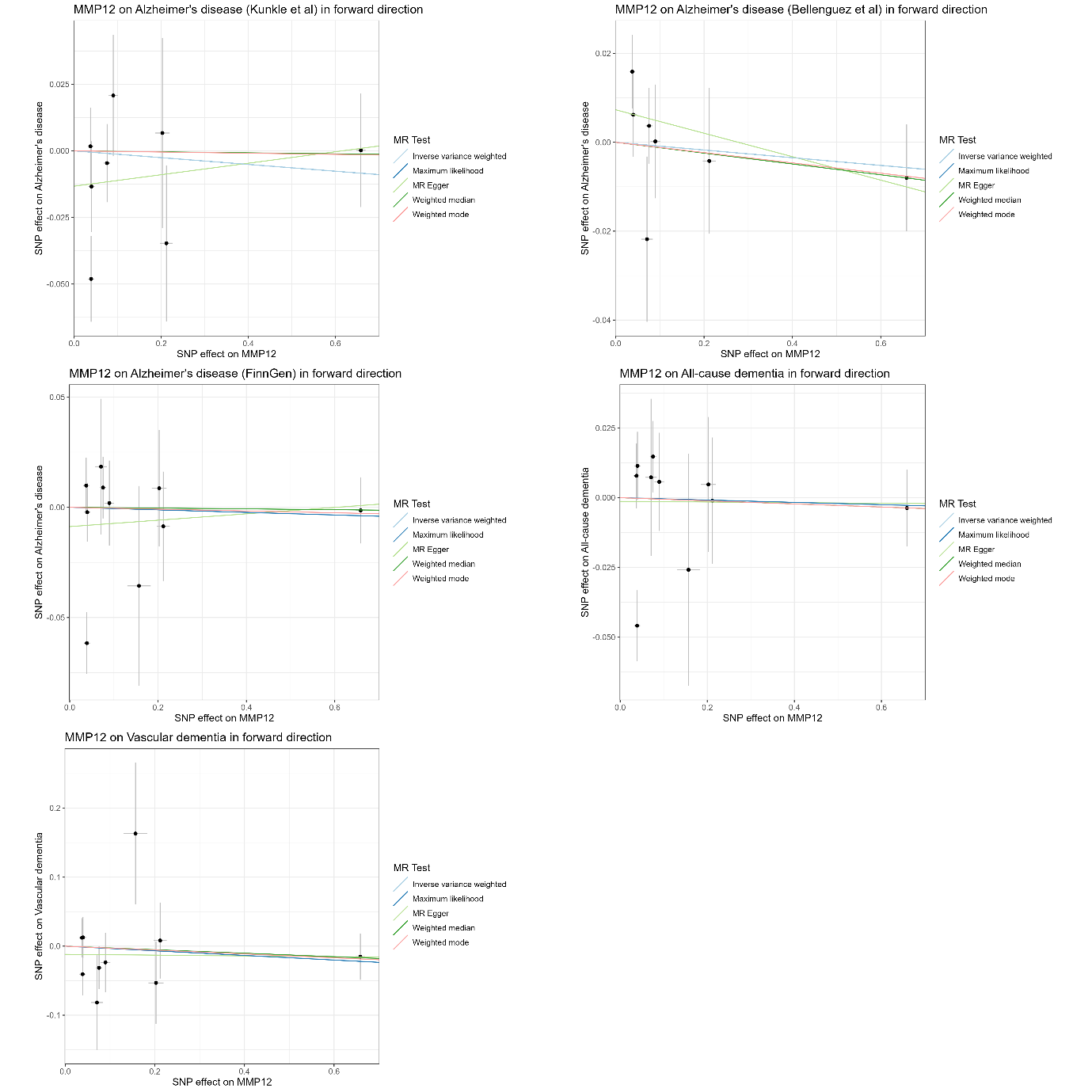


Analyses were conducted using the inverse variant weighted, maximum likelihood, MR-Egger, Weighted median, Weighted mode methods. The slope of each line corresponding to the estimated MR effect per method.

**Supplementary Figure 20. Two-sample Mendelian randomization in the reverse direction (dementia 🡪 protein concentration) scatter plots for four proteins NEFL in five GWAS for Alzheimer’s disease, all-cause dementia, and vascular dementia.**


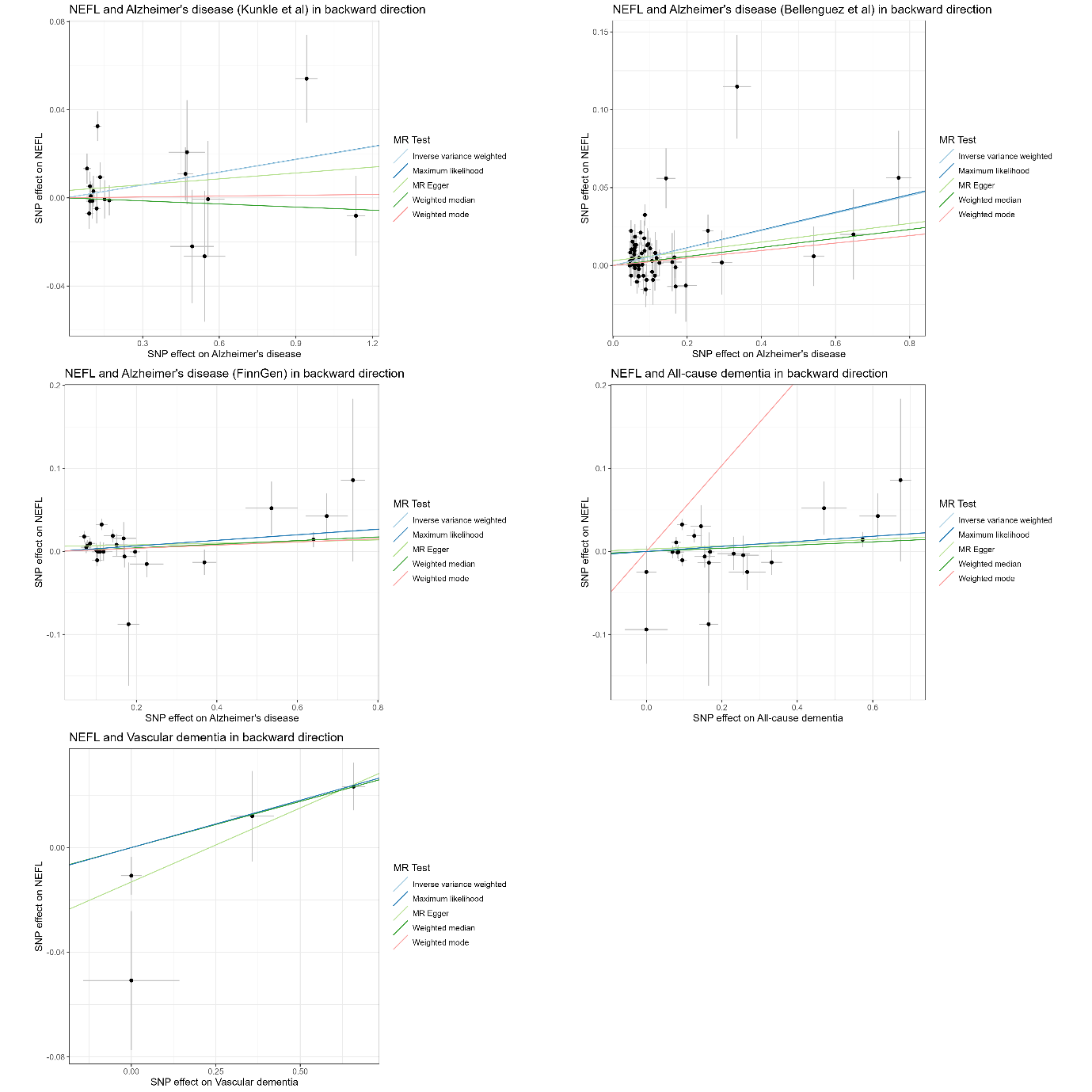


Analyses were conducted using the inverse variant weighted, maximum likelihood, MR-Egger, Weighted median, Weighted mode methods. The slope of each line corresponding to the estimated MR effect per method.

**Supplementary Figure 21. Two-sample Mendelian randomization in the reverse direction (dementia 🡪 protein concentration) scatter plots for KIM1 (HAVCR1) in five GWAS for Alzheimer’s disease, all-cause dementia, and vascular dementia.**


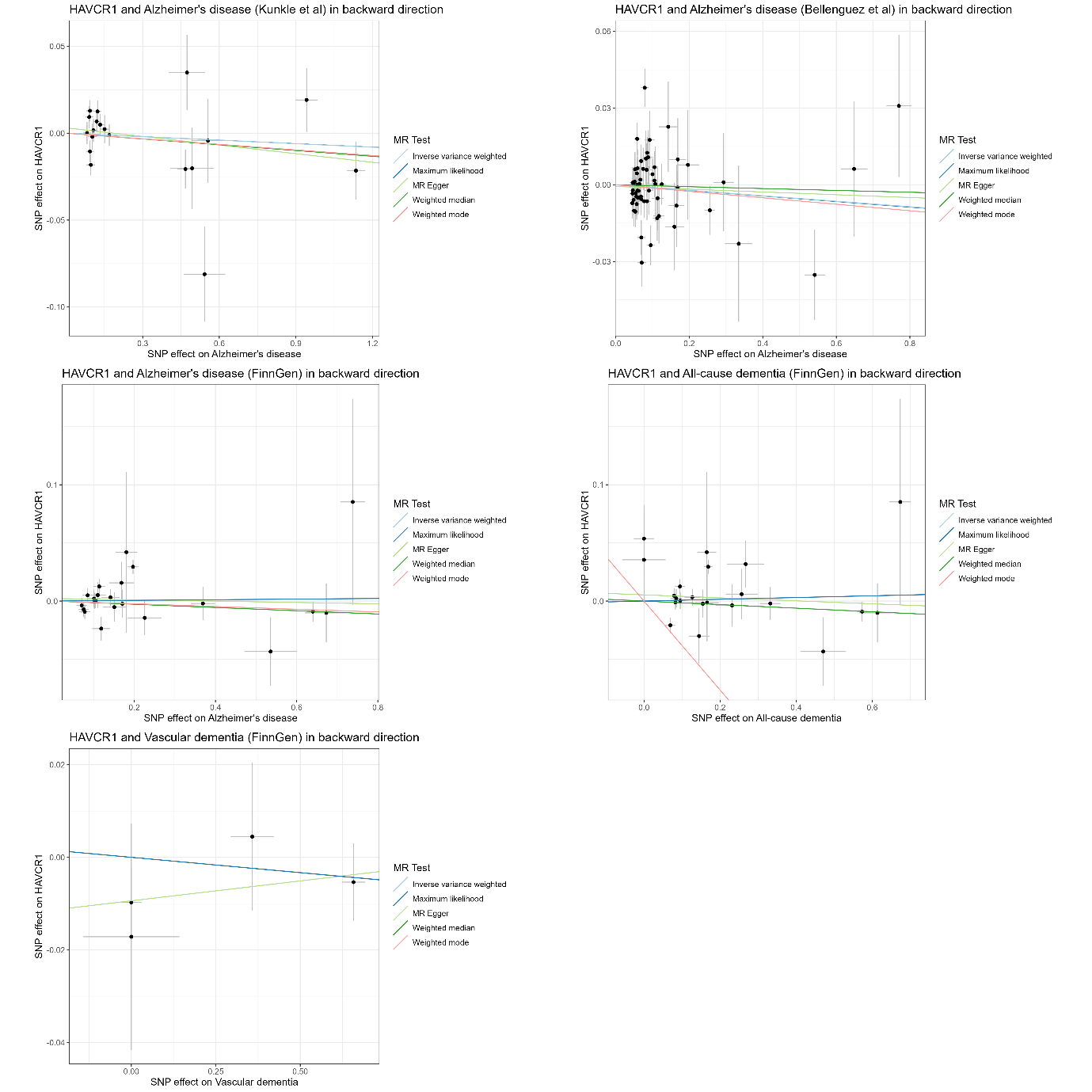


Analyses were conducted using the inverse variant weighted, maximum likelihood, MR-Egger, Weighted median, Weighted mode methods. The slope of each line corresponding to the estimated MR effect per method.

**Supplementary Figure 22. Two-sample Mendelian randomization in the reverse direction (dementia 🡪 protein concentration) scatter plots for EDA2R in five GWAS for Alzheimer’s disease, all-cause dementia, and vascular dementia.**


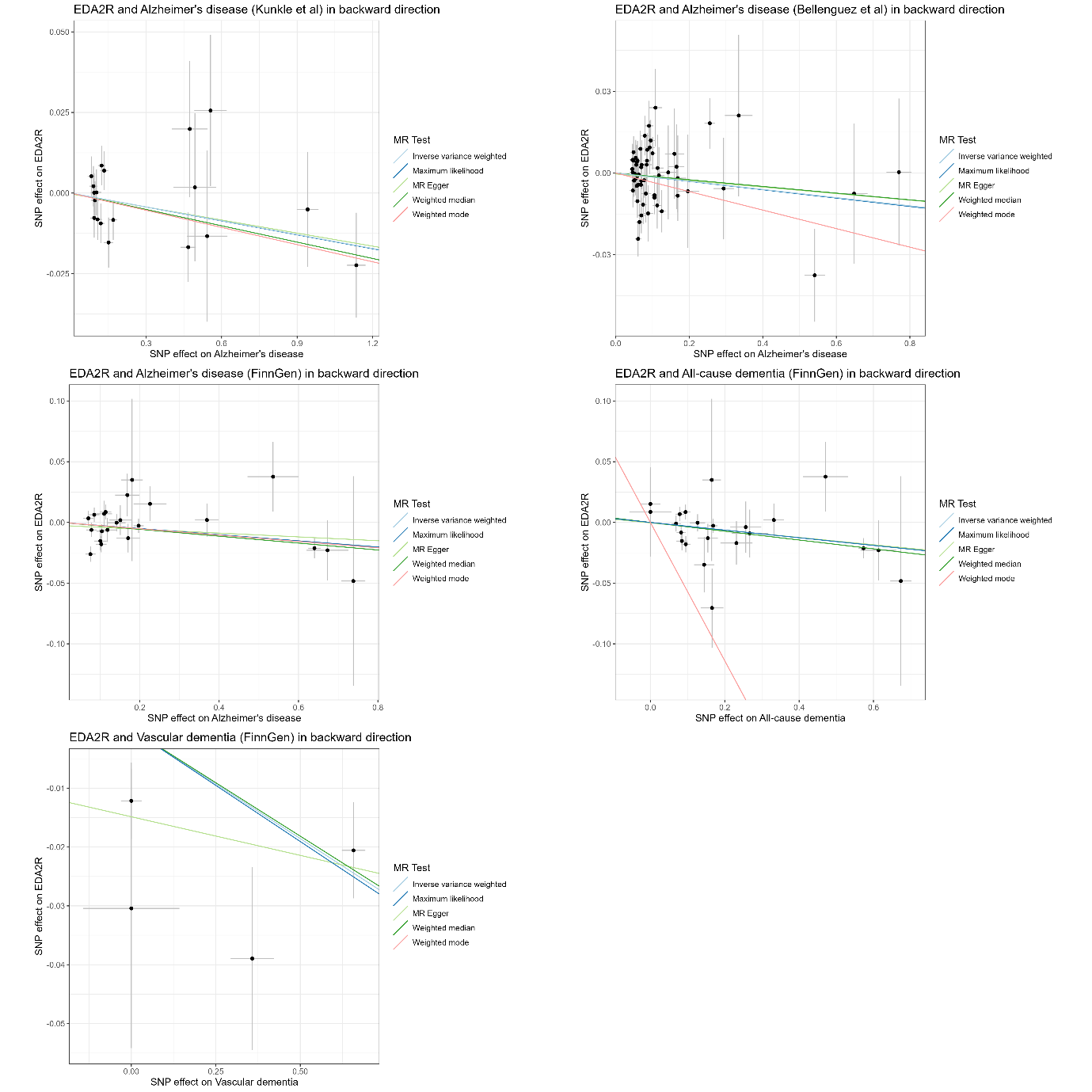


Analyses were conducted using the inverse variant weighted, maximum likelihood, MR-Egger, Weighted median, Weighted mode methods. The slope of each line corresponding to the estimated MR effect per method.

**Supplementary Figure 23. Two-sample Mendelian randomization in the reverse direction (dementia 🡪 protein concentration) scatter plots for MMP12 in five GWAS for Alzheimer’s disease, all-cause dementia, and vascular dementia.**


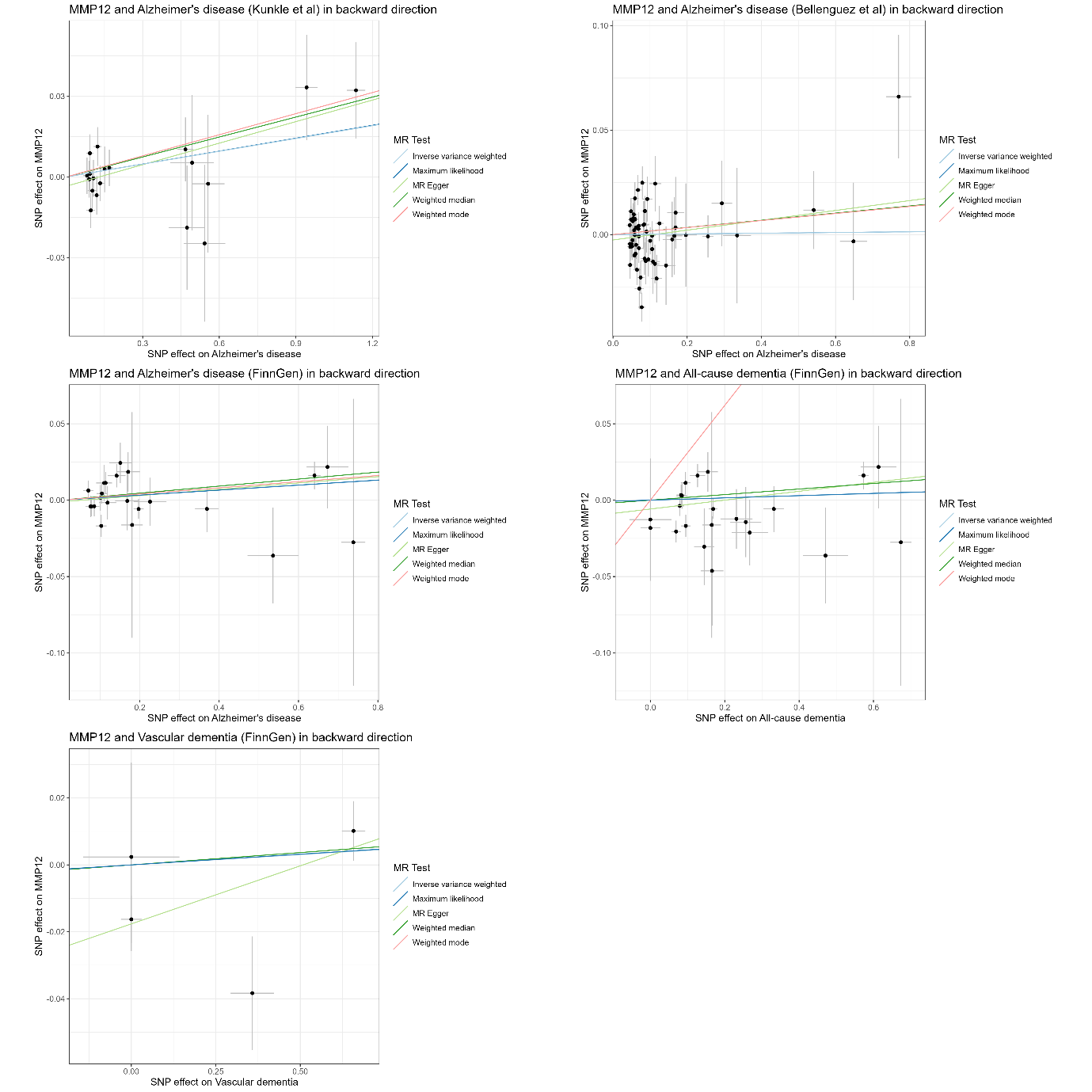


Analyses were conducted using the inverse variant weighted, maximum likelihood, MR-Egger, Weighted median, Weighted mode methods. The slope of each line corresponding to the estimated MR effect per method.

**Supplementary Figure 24. Two-sample drug target Mendelian randomization scatter plots for NEFL in five GWAS for Alzheimer’s disease, all-cause dementia, and vascular dementia.**


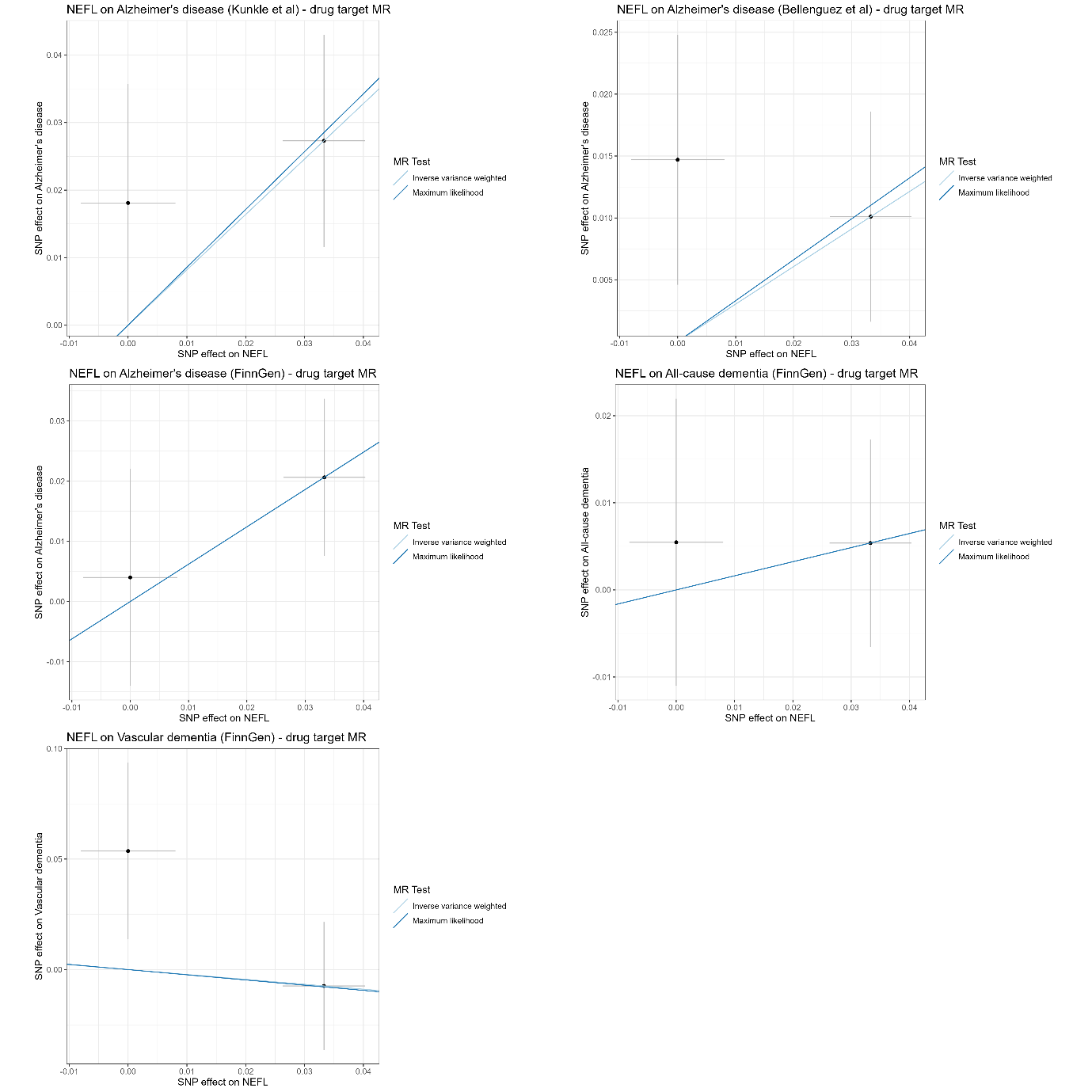


Analyses were conducted using the inverse variant weighted, maximum likelihood, MR-Egger, Weighted median, Weighted mode methods. The slope of each line corresponding to the estimated MR effect per method.

**Supplementary Figure 25. Two-sample drug target Mendelian randomization scatter plots for KIM1 (HAVCR1) in five GWAS for Alzheimer’s disease, all-cause dementia, and vascular dementia.**


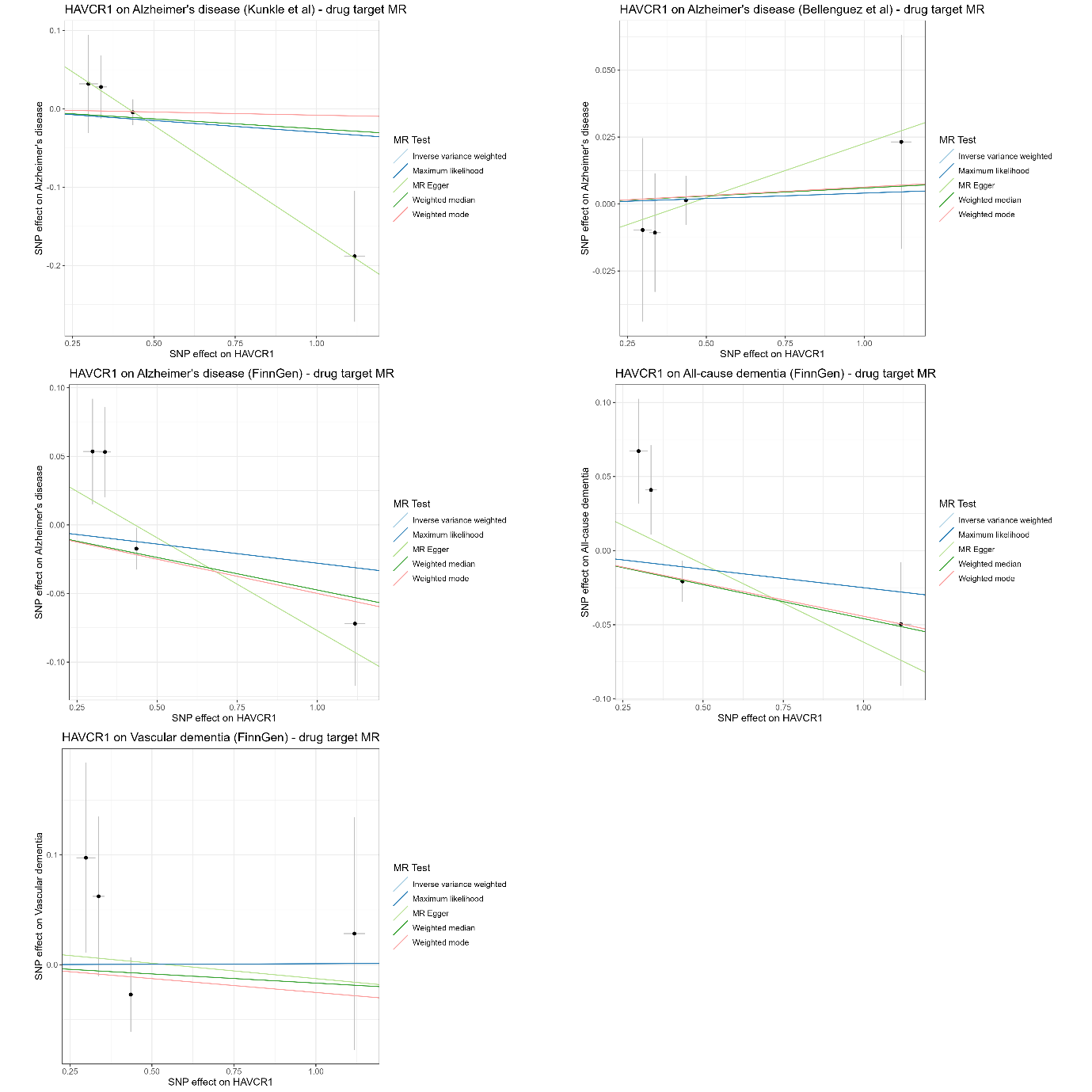


Analyses were conducted using the inverse variant weighted, maximum likelihood, MR-Egger, Weighted median, Weighted mode methods. The slope of each line corresponding to the estimated MR effect per method.

**Supplementary Figure 26. Two-sample drug target Mendelian randomization scatter plots for MMP12 in five GWAS for Alzheimer’s disease, all-cause dementia, and vascular dementia.**


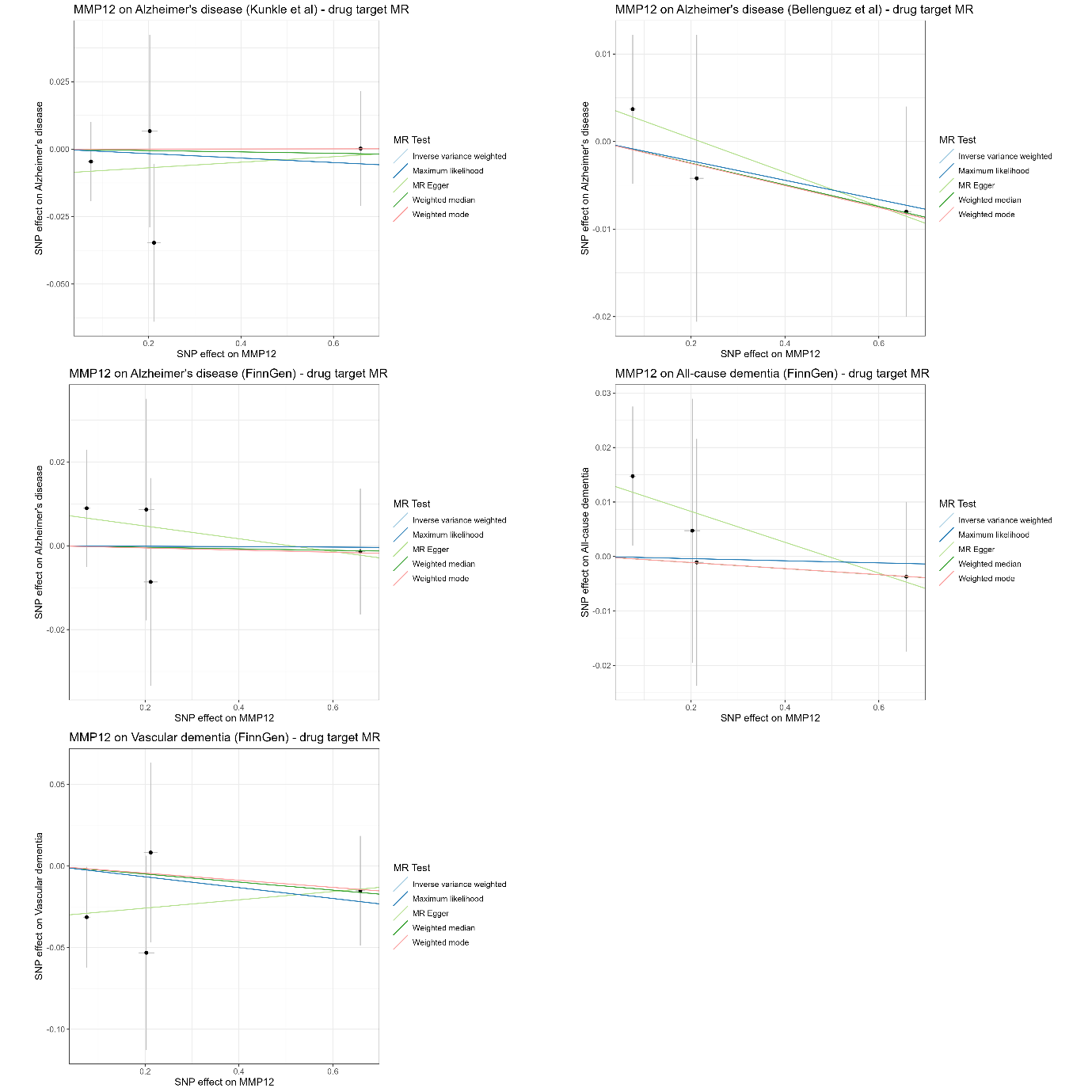


Analyses were conducted using the inverse variant weighted, maximum likelihood, MR-Egger, Weighted median, Weighted mode methods. The slope of each line corresponding to the estimated MR effect per method.
